# Bioportainer Workbench: a versatile and user-friendly system that integrates implementation, management, and use of bioinformatics resources in Docker environments

**DOI:** 10.1093/gigascience/giz041

**Published:** 2019-04-25

**Authors:** Fabiano B Menegidio, David Aciole Barbosa, Rafael dos S Gonçalves, Marcio M Nishime, Daniela L Jabes, Regina Costa de Oliveira, Luiz R Nunes

**Affiliations:** 1 Núcleo Integrado de Biotecnologia, Universidade de Mogi das Cruzes (UMC), Av. Dr. Cândido Xavier de Almeida Souza, 200, Mogi das Cruzes, SP - 08780-911, Brazil; 2 Centro de Ciências Naturais e Humanas, Universidade Federal do ABC (UFABC), Alameda da Universidade, s/n, São Bernardo do Campo, SP - 09606-045, Brazil

**Keywords:** Docker, bioinformatics, management user interface

## Abstract

**Background:**

The Docker project is providing a promising strategy for the development of virtualization systems in bioinformatics. However, implementation, management, and launching of Docker containers is not entirely trivial for users not fully familiarized with command line interfaces. This has prompted the development of graphical user interfaces to facilitate the interaction of inexperienced users with Docker environments.

**Results:**

We describe the BioPortainer Workbench, an integrated Docker system that assists inexperienced users in interacting with a bioinformatics-dedicated Docker environment at 3 main levels: (i) infrastructure, (ii) platform, and (iii) application.

**Conclusions:**

The BioPortainer Workbench represents a pioneering effort in developing a comprehensive and easy-to-use Docker platform focused on bioinformatics, which may greatly assist in the dissemination of Docker virtualization technology in this complex field of research.

## Background

The increasing use of computational methods for biological data analysis has revolutionized the study of biology in recent decades. However, demands for expensive high-performance hardware to run such analyses and the complexity associated with many software installations often represent major challenges to the widespread use of such resources among researches. Thus, server-based cloud computing and virtualization systems have been extensively used to minimize these problems. As a consequence, concepts of "platform as a service" (PaaS), provided by companies such as Google Genomics [1], Amazon AWS Genomics [2], or Microsoft Azure [3], as well as "software as a service" (SaaS), provided by initiatives such as the Galaxy Project [4] and Cloudman [5], are being increasingly adopted in research organizations with little or no bioinformatics capabilities, as well as in biotechnology and pharmaceutical companies worldwide, as a strategy to reduce costs and avoid the problems associated with installing and maintaining their own bioinformatics facilities [6]. Up to now, most of these bioinformatics-related PaaS/SaaS are based on virtual machines, which constitute a robust strategy to develop virtualization systems but have the drawback of consuming large amounts of disk space, display low scalability, and are difficult to implement in association with high-performance computing platforms. However, the emergence of the Docker Project [7] is providing a new and promising virtualization strategy that consumes considerably less disk space and provides the advantage of being platform-agnostic because it relies on the configuration of containers, which can be consistently interchanged and deployed on different computing environments, regardless of the specificities of their hardware and/or operating system, which helps to ensure replicability and reproducibility of data analyses across different research facilities.

Thus, several bioinformatics-related PaaS and SaaS initiatives based on Docker virtualization systems have been recently developed, such as BioShaDock [8], AlgoRUN [9], GUIdock [10], Dockstore [11], and BioContainers [12], among others. Even the Galaxy Project [4] has recently incorporated Docker technology to allow local installation of Galaxy containers [13] via Docker-based virtualization systems. More recently, this bioinformatics-as-a-service platform has been expanded by the development of Dugong [14], which introduced the concept of "desktop as a service" in bioinformatics analyses. Other projects, such as Snakemake [15], Common Workflow Language [16], and NextFlow [17] have provided frameworks for the implementation, administration, and execution of complex pipelines and workflows within Docker containers, allowing simultaneous management and coordinated launching of the various software involved in such analyses.

However, in spite of the advantages provided by Docker and other container-based virtualization systems, they are not easily implemented by inexperienced users, mostly as a result of poor familiarity with the Docker Engine computational environment, which is based on a command line interface (CLI); this difficulty is compounded by the lack of proper documentation for many Docker applications and the predominance of non-standardized images, which are built upon Dockerfiles containing obscure implementation steps. Moreover, the complexity of Docker systems tends to increase when environments are composed by different containers or involve the implementation of Swarm, a native Docker tool that allows the creation of clusters composed by different Docker hosts within the same resource pool [18,19].

To overcome such difficulties, different initiatives have sought the development of graphical user interfaces (GUIs) aimed at helping inexperienced users to interact with Docker environments at different levels. For example, projects such as Panamax [20], Shipyard [21], Rancher [22], and Portainer [23] have provided GUIs to assist users at the infrastructure level because they allow easy implementation and resource management of Docker environments.

The Galaxy Project [4], on the other hand, seeks to provide a platform based on a user-friendly web GUI for software launching, but its implementation is not a trivial action because a simple Galaxy installation requires considerable disk space and complex installation steps for its full operation. Finally, projects like AlgoRUN [9] tried to facilitate the launching of CLI-based applications by the development of dedicated and customizable GUIs, but the requirement of manual installation of individual software in their respective containers, without the help of a framework such as Conda [24] and repositories such as Bioconda [25], may have prevented its full adoption by the Bioinformatics community.

In this scenario, this article describes the BioPortainer Workbench [26], an integrated Docker system that seeks to assist inexperienced users to interact with a bioinformatics-dedicated Docker environment at 3 main levels: in the Infrastructure Layer, the BioPortainer Workbench [26] provides a GUI, based on the Portainer project [23], which allows rapid and simple implementation/management of a full Docker ecosystem; in the Platform Layer, the software provides a wide range of intuitive template forms that assist users in the installation and configuration of containers, carrying specific bioinformatics tools, from a variety of alternative platforms (based on CLIs, GUIs, or on a virtual desktop); finally, in the Application Layer, the BioPortainer Workbench [26] provides a series of CLI-based and GUI-based interfaces to assist users in launching jobs with varying degrees of complexity (from single application analyses to complex pipelines and workflows).

## BioPortainer Workbench Architecture

**Figure 1:**
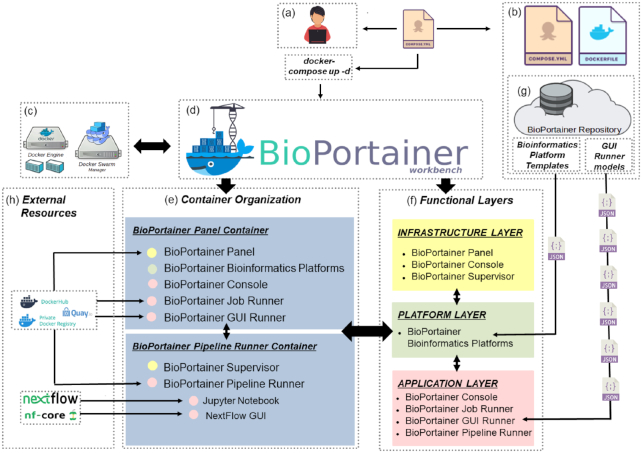
Overview of the BioPortainer Workbench Architecture: Users (a) can access the BioPortainer Workbench image (b) from the project’s web page and install the software either in single Docker engines or in Swarm clusters (c). Once Installed, the BioPortainer Workbench (d) consists of 2 containers (e): the BioPortainer Panel and the BioPortainer Pipeline Runner. These 2 containers provide access to a series of tools that operate in 3 distinct functional layers (f), allowing users to implement bioinformatics-related tools (using a variety of alternative platforms), manage resources from the Docker environment, and launch their analyses using both CLI-based and GUI-based interfaces (preconfigured through a series of JSON files that are available from the BioPortainer Workbench repository [g]), as well as commands/scripts/pipelines harnessed from external repositories (h). See text for details.

BioPortainer Workbench [26] (BioPortainer, RRID:SCR_017058) is an open-source software package developed under the MIT license and designed in a modular way, aimed at facilitating user interaction with Docker environments in 3 different computational layers: (i) infrastructure, (ii) platform, and (iii) application. Its basic structure is briefly described in Fig. 1. To deploy the software, the user must initially access the BioPortainer Workbench image (Fig. 1b) and install it, either in a single Docker engine or in a Swarm cluster (Fig. 1c). Once installed, BioPortainer Workbench [26] (Fig. 1d) consists of 2 basic containers: the BioPortainer Panel and the BioPortainer Pipeline Runner (Fig. 1e). From a functional point of view (Fig. 1f), the 2 containers offer a number of tools that allow users to perform a series of actions in the created Docker environment, such as (i) managing Docker resources associated with the BioPortainer Workbench [26]; (ii) installing bioinformatics applications based on several platforms described in the literature; and (iii) launching different types of analyses, using either CLIs or GUIs. Such analyses may be conducted with the help of BioPortainer Workbench’s own resources (Fig. 1g) or with resources harnessed from external repositories, which provide preconfigured images, files, commands, or scripts for the execution of bioinformatics software, with varying levels of complexity (Fig. 1h).

The BioPortainer Workbench image, as shown in Fig. 1b, consists of a Docker Compose file, which is responsible for building the Docker environment in the host machine, using Dockerfile files, which are associated with the 2 main containers of the software: the BioPortainer Panel and the BioPortainer Pipeline Runner. The third component of the image is a template repository, containing a series of JavaScript Object Notation (JSON) files, responsible for building the templates associated with platform installation, as well as for building the GUI forms used to launch bioinformatics tools, with the aid of the BioPortainer GUI Runner (see details below).

The Docker Compose file contains all the necessary settings for complete system operation, including the execution settings of the 2 main containers (BioPortainer Panel and BioPortainer Pipeline Runner). In addition to these 2 main modules, there is a third module (not shown in Fig. 1) called the BioPortainer Watchtower, which is designed to monitor the main containers during execution. This module also monitors the original BioPortainer Workbench images, updating the whole Docker environment whenever new versions of the software are made available. The Docker Compose file can be easily edited with the aid of any text editor, so as to expand its functionality by incorporating additional features, such as web proxies and/or tools for continuous integration and continuous delivery, e.g., Jenkins [27]. To assist in the incorporation of these new resources, a virtual network, called BioPortainer local, was created in order to guarantee efficient communication between the containers. Thus, if users wish to incorporate new features into the BioPortainer Workbench [26], it is necessary to insert such features in this network, through the networks parameter, to guarantee their efficient integration to the different modules of the software.

The second component of the image is the Dockerfile files, which contain instructions for building the Docker images of the BioPortainer Panel and BioPortainer Pipeline Runner modules. These Dockerfiles are available through GitHub and can be easily expanded to accommodate additional needs of any user. The Dockerfile of the BioPortainer Panel module was developed from the original source file of Portainer [23], which has been modified to incorporate specific features of the BioPortainer Workbench [26], such as the tools BioPortainer Job Runner and BioPortainer GUI Runner. The Dockerfile of the BioPortainer Pipeline Runner, on the other hand, has been developed independently and presents a greater level of complexity in its structure, as it carries all the software and library prerequisites necessary for the execution of NextFlow scripts [17] in a Docker-in-Docker environment. In addition, given the characteristics of this complex environment, the BioPortainer Supervisor tool (not shown in Fig. 1) has been added to this Dockerfile, providing users with a GUI (accessed through port 7,000) that enables full management of the BioPortainer Pipeline Runner tool, the Jupyter Notebook [28], and the Docker-in-Docker environment. It also allows users to analyze execution logs and control startup, shutdown, and restart of processes within containers.

The third component of the image is the BioPortainer Repository [26], consisting of a series of JSON files. The JSON [29] language allows storage of data structures in a standard interchange format, which can be used for transmitting data between a server and a graphical web interface application. One of these JSON files is responsible for generating the GUI templates that will assist users during the installation of the BioPortainer Bioinformatics Platforms (accessible through the BioPortainer Panel main menu). The BioPortainer Repository [26] also carries additional JSON files that provide users with GUIs containing the interactive forms that assist in the launching of bioinformatics tools through the BioPortainer GUI Runner (see below). All JSON files available at the BioPortainer Repository [26] were manually developed to ensure perfect adaptation to the specific environmental variables and parameter prerequisites of their specific platforms/tools. Moreover, all of them were individually tested by expert curators and further evaluated by continuous integration and continuous delivery, using the tools TravisCI [30] and CircleCI [31]. Users interested in modifying the BioPortainer Workbench [26] JSON files, in order to expand their resources and/or adapt them to new needs, can obtain the individual files from the BioPortainer Workbench project web page [26].

## Installing and Configuring the BioPortainer Workbench

Installation of the BioPortainer Workbench [26] is extremely simple and initially requires only Docker and Docker Compose. After both components are installed, only 2 steps are needed to start a BioPortainer Workbench environment. In the first step, the compose.yml file is downloaded from the server (GitHub or BioPortainer Workbench home page [26]) to the BioPortainer folder in the host machine. In the second step, the Docker Compose is executed, downloading the images for the standard BioPortainer Workbench modules, and the service is started. When Linux is the host machine’s operating system, the following commands must be run on the terminal:

$ wget https://goo.gl/bNecPA -O docker-compose.yml

$ docker-compose up -d

During the deployment process, some ports and disk volumes will be automatically configured in the host machine. Details on the ports and volumes created are available in the BioPortainer Workbench User Manual, which accompanies this article as a supplementary file. In a standard implementation, the BioPortainer Workbench [26] will use the localhost address (IP 0.0.0.0) as the default address for its internal links. If access is not performed through a local network, additional settings must be created in the Compose file, according to Docker’s official documentation [7].

To simulate and test the implementation, administration, and operation of the BioPortainer Workbench [26], we emulated its deployment in a test environment, using the Play-with-Docker [32] testing platform. The installation processes, as well as the results from this test, can be viewed through video files available at the BioPortainer Workbench project web page [26]. These videos demonstrate that, once installed, BioPortainer Workbench [26] is fully functional and can be readily used for container implementation/administration, through the BioPortainer Panel, or for the execution of a variety of (simple or complex) bioinformatics analyses, using any of its various implementation tools, which are described in the next section (see the BioPortainer Workbench User Manual for details).

## Features and Functionalities of the BioPortainer Workbench

As mentioned above, the BioPortainer Workbench [26] aims not only to provide Bioinformatics tools in a platform-agnostic environment, such as Docker, allowing its implementation in any type of computing ecosystem, but also to deliver a friendly interface that allows users to interact with this environment in different functional layers: (i) infrastructure, (ii) platform, and (iii) application. Thus, description of the features and functionalities of the BioPortainer Workbench [26] follows this same rationale during the next sections.

### The infrastructure layer: Implementing and managing a Docker environment with the aid of the BioPortainer Panel

The proper management of Docker resources is often a serious problem for inexperienced users because it is usually performed through a CLI. However, such management is essential for the operation of the host system because downloading images and creating volumes in a non-transparent way can lead, for example, to excessive consumption of disk space, leading to rapid degradation of the host machine’s environment. In addition, inadequate management of disk volumes can increase the number of orphan volumes in the system, which will continue to occupy physical space even after removal of the containers to which they were linked.

To overcome such obstacles, the BioPortainer Workbench [26] provides a graphic interface to assist users in the management and distribution of such resources, through the BioPortainer Panel module, which is composed of different submodules (see BioPortainer Workbench User Manual). For example, the BioPortainer Dashboard (Fig. S4 in the manual) provides quick and general information about the managed host (either a single Docker host or a Swarm cluster), such as (i) version of the installed Docker engine; (ii) amount of memory and central processing unit (CPU) available; (iii) number of containers, images, volumes, and networks available; and (iv) details on the use of such resources by each container, image, and network, allowing users to optimize their distribution among different applications. The Containers option (Fig. S5 in the manual), on the other hand, provides button interfaces that allow the user to start/stop/restart and kill jobs using the available containers, along with other specific commands for their full management and administration.

In addition to assisting in the optimal distribution of resources, the BioPortainer Panel Dashboard also allows the use of alternative runtimes. Among these runtimes, we highlight the possibility of using NVIDIA-Docker [33], a Docker Engine plug-in designed to facilitate the deployment of containers capable of using graphical processing units (GPUs) as their main processing units. The use of this runtime can be very helpful while running jobs that require high processing capacity, which can be achieved through the use of GPUs rather than the system’s CPUs (which are traditionally used by most computer applications). To accomplish that, the plug-in automatically recognizes GPU devices in the host, as well as their drivers and volume-mounting points, directing the execution of container services to GPU drives rather than CPUs. To further facilitate the use of GPU resources, the BioPortainer Workbench [26] provides a template platform, developed with the aid of CUDA [34] and CUDNN [35] libraries, ready to work with NVIDIA-Docker [33], enabling the creation, debugging, and performance optimization of NVIDIA-GPU–accelerated applications (see below). These templates enable complete integration between the Docker and GPU environments with both Conda [24] and PIP [36] package managers because they have a set of tools developed in Python.

### The platform layer: Implementing containers carrying fully functional bioinformatics tools with the aid of the BioPortainer bioinformatics platforms

The installation of several bioinformatics tools can be done through alternative platforms, many of them extensively described in the literature [4,9,10,12,14]. Some of these platforms provide access to large software repositories such as Bioconda [25], LinuxBrew [37], or BioConductor [38], for example, which allow the installation of thousands of generic bioinformatics tools. On the other hand, there are also platforms dedicated to repositories focused on more specific applications, enabling the installation of languages (such as R or Shiny) or accessories (such as the Jupyter Notebook [28]), for example, which can be integrated into different types of analyses (see below). Moreover, some of these platforms have specificities that differentiate them from one another, including, e.g., the use of interfaces based on CLI (BioContainers [12]), GUI (Galaxy [4]), or virtual desktop (Dugong [14]), providing users with a variety of alternatives for container creation.

The BioPortainer Bioinformatics Platforms option (Fig. S7 in the manual) provides access to all Bioinformatics Platform Templates available at the BioPortainer Repository. From this menu, users have access to intuitive template forms that assist in the installation and configuration of containers, carrying specific bioinformatics tools, using whichever platform they deem most convenient to their needs and/or expertise. As mentioned above, these templates are defined by a preconfigured JSON file that is available from the BioPortainer Repository [26]. Using these templates, users can implement and configure containers carrying single tools or a set of related tools (described in a single Compose file) in any local Docker engine. Although considered easy to build and implement when compared to other files with the same purpose, the JSON format can become extremely complex for inexperienced users because it is based on a subset of JavaScript programming language. Thus, the availability of preconfigured models minimizes problems associated with implementation and configuration of tools within containers and improves customization to the unique requirements of each platform/software to be implemented. Nonetheless, it is possible to add new templates and/or update/modify existing templates in an easy and intuitive way with the help of options such as add template and update template, which are available through the BioPortainer Panel main menu (see Fig. S7 in the manual).

Currently, the BioPortainer Bioinformatics Platforms option provides 11 Docker-based bioinformatics platforms (see Table S1 in the manual), which allow access to a wide variety of tools that can be accessed, installed, managed, and launched with the aid of the BioPortainer Workbench [26]. These platforms include the following:

- The BioPortainer CPU Platform provides pre-configured templates aimed at creating images/containers carrying bioinformatics tools combined with features that help to ensure reproducibility and replicability in data analysis. These include screen, GNU parallel, script/scriptreplay, and Jupyter Notebook [28], among others. In addition, they allow users to install the Miniconda2 and Miniconda3 packages, enabling the deployment and use of >2,000 bioinformatics tools developed in Python that are available through the Bioconda [25] repository.
- The BioPortainer GPU Platform is similar to the CPU Platform but provides pre-configured templates aimed at creating images/containers with the help of the CUDA [34] and CUDNN [35] library kits. As a result, such containers can be processed through GPU units using the NVIDIA-Docker [33] plug-in, as described above.
- The BioPortainer GUI Runner Platform allows users to implement containers to be launched with the aid of the BioPortainer GUI Runner. This tool (described in detail in the next section) allows users to launch >100 bioinformatics applications through a user-friendly GUI, dismissing the use of CLI-based interfaces.
- The Galaxy Platform provides templates that allow the implementation of 25 versions of galaxy images, carrying software for performing a variety of -omics analyses such as transcriptomics, phylogenenomics, proteomics, and metagenomics, among others [4,39,40]. All tools available on this platform are fully adapted to work through the Galaxy interface and, thus, do not require the use of CLI-based interfaces. (The BioPortainer Workbench User Manual provides detailed instructions on how to access the Galaxy Stable user interface after installing this tool in a Docker container).
- The Galaxy Tools Platform features templates for the development and implementation of additional software on the main Galaxy instance [4] with the help of Planemo [41], a command-line utility that helps to create and publish new Docker-based Galaxy tools [4].
- The BioContainers Platform contains a template for assisting users to deploy the main Docker image of BioContainers [12], a CLI-based platform that allows installation and distribution of >2,000 bioinformatics tools (available from the Bioconda [25] repository) within Docker containers.
- The Dugong Platform provides templates that assist in the implementation of different versions of Dugong [14], a Docker-based virtual desktop that helps users to deploy Docker containers carrying >3,500 bioinformatics software programs (available from the Bioconda [25], LinuxBrew [37], and BioLinux [42] repositories), directly integrated with the Jupyter Notebook [28].
- The GUIdock Platform contains templates to assist in the implementation of different versions of GUIdock, a Docker image dedicated to providing graphical analytical tools (particularly suitable for network analyses) inside containers [10].
- The Bioconductor Platform provides templates for the rapid implementation of images/containers carrying a variety of bioinformatics tools that can be used in the R language environment, available from the Bioconductor repository [38].
- The R and RStudio Platform [43,44] provide templates for the implementation of images/containers carrying the R and Shiny languages, along with the standard installation of Rstudio, providing a complete environment for running Bioconductor tools and/or performing statistical analyses of large datasets.
- The Jupyter Notebook Platform provides templates to assist in the implementation of standard Docker images/containers carrying the Jupyter Notebook [28], along with the main tools/languages currently used in the development of bioinformatics protocols (e.g., Python, R, Scala, Spark, Mesos, and Tensorflow, among others).

All templates offer custom options in their deployment forms and are specially designed to meet the needs of the different tools available, such as port/volume mapping and network configurations, among others. The BioPortainer Workbench User Manual provides detailed instructions for installing containers carrying bioinformatics tools from the BioPortainer Bioinformatics Platforms. Two examples are used to illustrate this process: (i) the installation of a container carrying the Galaxy Stable tool from the Galaxy Platform and (ii) the installation of a container carrying the Dugong Clean CMD from the Dugong Platform [14].

### The application layer: Launching bioinformatics analyses from Docker containers

Launching of bioinformatics analyses can be performed using 4 different tools that are available from the BioPortainer Workbench [26]: (i) BioPortainer Console, (ii) BioPortainer Job Runner, (iii) BioPortainer GUI Runner, and (iv) BioPortainer Pipeline Runner.

### Launching bioinformatics analyses with the aid of the BioPortainer Console

The BioPortainer Console represents the simplest alternative for launching simple analyses, normally involving a single bioinformatics tool. Once a container carrying a specific tool has been implemented, users only need to click the Console icon (>_), available from the Containers option of the BioPortainer Panel main menu (see Fig. S2 in the manual), which will cause a CLI-based interface to pop up. Next, users can simply enter the necessary commands to run the application at the Bash command prompt. This tool was ported directly from the Portainer project [23] and is a useful option for users well versed in Linux who wish to save time during analyses by avoiding the use of GUIs. The BioPortainer Console also provides a simple and fast interface for full interaction and administration of containers without the need to install any external or internal tools, such as servers and clients for the SSH protocol. A detailed demonstration on how to use the BioPortainer Console to launch an alignment of RNA sequencing (RNA-seq) data against a reference genome using the BWA aligner is shown in the BioPortainer Workbench User Manual.

### Launching bioinformatics analyses with the aid of the BioPortainer Job Runner

Like the BioPortainer Console, the BioPortainer Job Runner is intended for users familiar with Linux commands. However, while the former is more suited for running simple analyses using individual tools/containers, the latter is configured to perform more complex analyses involving various steps and software. This tool uses, as target, a preconfigured Docker image that contains all the tools necessary for the analysis in question. The BioPortainer Job Runner interface can be accessed through the BioPortainer Panel main menu and is shown in Fig. S12 of the manual. To trigger this tool, users must initially provide the target Docker image (which may be present either in the host machine or in external repositories such as DockerHub). Next, users must provide an execution script, which can be either typed into the web-editor interface or imported through the upload option (see Fig. S12 in the manual). Finally, by clicking on "execute," users will have the script executed within a container built from the selected image. At the end of the process, the job’s output will also be present within this container, which can then be converted into a Docker image to be shared through public or private repositories, such as DockerHub or Quay.io. Thus, the BioPortainer Job Runner allows users to encapsulate entire executions within a single exchangeable Docker image/container, contributing to promote replicability and reproducibility of data analyses across laboratories (the entire process can also be tracked through Job Runner’s own job history). A detailed demonstration on how to use the BioPortainer Job Runner to launch a full differential expression analysis with RNA-seq data, using the Tuxedo Suite, is shown in the BioPortainer Workbench User Manual.

### Launching bioinformatics analyses with the aid of the BioPortainer GUI Runner

The GUI Runner is a specific tool of the BioPortainer Workbench [26] and was developed in Python 3.4 (or higher), Tornado 4 (or higher), and Typing. It allows users not well versed in Linux commands to launch bioinformatics tools from their respective containers with the help of intuitive GUIs. These interfaces are configured from specific JSON files, each of them specifically developed for a particular tool. Altogether, the BioPortainer Repository [26] contains preconfigured JSON files that enable the launching of 109 bioinformatics tools from the GUI Runner, which can be used for a wide range of Bioinformatics analyses, such as quality control trimming of next-generation sequencing data, gene identification through hidden Markov models, computation of guanine-cytosine bias across genomes, and many others (a complete list of tools that can be currently launched with the aid of the BioPortainer GUI Runner can be seen in Table S2 in the BioPortainer Workbench User Manual).

The GUI Runner templates are arranged in 2 folders: (i) conf/runners/, which contains the JSON files used to build the GUIs; and (ii) conf/scripts/, which contains the execution scripts that receive the variables typed in these GUIs prior to execution. As mentioned above, the current version of the BioPortainer Repository [26] contains GUI templates that allow 109 bioinformatics tools to be launched by the BioPortainer GUI Runner, but our team is continually working to increase this number in further releases of the Repository. Users interested in developing new JSON files to extend the scope of GUI Runner to new analytical tools can find instructions on how to download a basic JSON model in the BioPortainer Workbench User Manual. It is important to note, however, that new JSON files will only become functional after being transferred to the conf/runners/ folder. Moreover, each JSON file must be unique and represent a particular script or tool. Thus, to launch a container containing 2 different tools, users must develop a specific JSON file for each tool.

Finally, access to the BioPortainer GUI Runner can be achieved in 2 ways: through the GUI Runner option, available from the BioPortainer Panel Bioinformatics Platforms menu (see Fig. S7 in the manual); or by clicking on port 5,000 of the container in question, through the Containers option (see manual for details). A detailed demonstration on how to use the BioPortainer GUI Runner to launch a FASTQC analysis from a next-generation sequencing dataset is shown in the BioPortainer Workbench User Manual.

### Launching bioinformatics analyses with the aid of the BioPortainer Pipeline Runner

The Bioportainer Pipeline Runner is dedicated to helping inexperienced users to conduct complex analyses involving multiple bioinformatics tools, connected through pipelines and/or workflows, in a Docker ecosystem. Although implemented in a separate container, all its features, including volumes and environment variables, are also managed and monitored through the Bioportainer Panel.

The BioPortainer Pipeline Runner is accessed through the BioPortainer Panel main menu (see Fig. S3 in the manual), providing users with a graphical interface that allows the download, through a GIT protocol, of bioinformatics pipelines available at the NextFlow and NF-Core repositories. Both repositories are developed by collaborative projects that use NextFlow [17] (a type of domain-specific language) to develop and adapt scalable and reproducible scientific workflows using software containers. Thus, several pipelines developed by these projects include scripts and images specifically developed for complex bioinformatics analyses, such as (i) 16S ribosomal RNA amplicon sequence analysis using QIIME, (ii) identification and quantification of peptides from mass spectrometry raw data, (iii) human leukocyte antigen typing from next-generation sequencing data, and (iv) chromatin immunoprecipitation analysis, among many others. Overall, >80 high-quality bioinformatics pipelines are available from the NextFlow and NF-Core repositories. Once activated, the BioPortainer Pipeline Runner GUI provides different user options through its menu, which allow users to download, execute, monitor, update, and delete NextFlow-based pipelines from the abovementioned repositories, or harnessed from alternative Github sources (details regarding the options to run the BioPortainer Pipeline Runner can be found in the BioPortainer Workbench User Manual).

The incorporation of NextFlow [17] as the main framework for the Bioportainer Pipeline Runner was facilitated by its easy integration with the Docker project. Moreover, the NextFlow [17] community is extremely active, which should help to expand the number and scope of available pipelines over the next years. In this sense, we understand that the BioPortainer Workbench [26] may contribute to fostering the utilization of such pipelines by inexperienced users.

Alternatively, users may choose to launch their analyses through the BioPortainer Pipeline Runner using a protocol previously implemented in the Jupyter Notebook [28]. To accommodate this possibility, the BioPortainer Workbench [26] provides a functional installation of the Jupyter Notebook [28], in conjunction with the Python3 kernel and the Bash kernel. By using such resources, the BioPortainer Pipeline Runner becomes an extremely flexible tool, allowing the construction and execution of complex pipelines using libraries and tools developed in both NextFlow [17] and Python languages. Moreover, notebooks created through this approach can be shared among laboratories, ensuring replicability of the entire computing environment used for data analysis. Implementation of the Jupyter Notebook [28] is linked to a token that is automatically generated during startup of the Docker engine, and detailed instructions for connecting it to the BioPortainer Pipeline Runner can be found in the BioPortainer Workbench User Manual.

To demonstrate the full functionality of the BioPortainer Pipeline Runner, we ran 2 pipelines obtained from the NextFlow [17] project home page. One of them (the "rnatoy" pipeline) has been designed to conduct a workflow for RNA-seq analysis using the Tuxedo Suite. The second pipeline (named "nmdp-flow") performs variant-calling analyses with RNA-Seq data and is based on BWA [45] and SAMtools mpileup [46]. Detailed demonstrations on how to launch these pipelines (using both the BioPortainer Pipeline Runner GUI and the Jupyter Notebook [28]) are shown in the BioPortainer Workbench User Manual.

Finally, it should be mentioned that, in spite of its wide flexibility, the BioPortainer Pipeline Runner was designed as a tool for performing workflow analyses based on Docker. Thus, NextFlow [17] executions based on the Singularity container system [47] cannot be performed through this version of the software.

## Discussion

Bioinformatics lies at the intersection of biology, computer science, and statistics and often attracts professionals with limited skills for the appropriate management of computational environments. Although several initiatives have recently demonstrated the viability of using Docker to provide bioinformatics tools to researchers, most of these Docker-based systems have been developed with little concern for inexperienced users, limiting their widespread implementation in research facilities. For example, Docker image repositories such as BioShaDock [8] and Dockstore [11] provide several bioinformatics software programs within Docker containers, but local installation of images still requires adjustments to ensure their full operation, such as the export of network service ports and configuration of data volumes, among other procedures, which are often unclear to the final user owing to lack of proper documentation in such repositories. Moreover, the absence of proper standards for image generation and lack of curatorship led to the accumulation of heterogeneous tools in these repositories. Some of these problems were addressed by the development of standardized Docker images by BioContainers [12], which allows access to >2,000 bioinformatics tools from the Bioconda [25] repository. However, BioContainers [12] operates exclusively through command lines, hampering its use among users not well versed in Linux commands. Although future development of BioContainers [12] may lead to its integration with the Galaxy graphical interface through Galaxy Interactive Environments [48], Galaxy Interactive Environment deployment is not a trivial operation because they have complex interactions with numerous services. Moreover, implementation of the Galaxy instance [4] requires a large amount of memory and disk space and additional Galaxy tools [4] have different requirements in computer memory, I/O speed, disk space, network bandwidth, density of computing cores, and parallel environment configurations, among other issues.

Thus, the development of specific tools capable of assisting inexperienced users is of paramount importance to ensure the widespread use of Docker-based bioinformatics resources. These tools may greatly contribute to improving the replicability and reproducibility of data analysis, given the platform-agnostic nature of Docker systems. In fact, the widespread use of Docker in various corporate business environments has been stimulated by such initiatives as Panamax [20], Shipyard [21], Rancher [22], and Portainer [23], which developed graphical interfaces to help in the implementation, administration, and management of Docker environments by less experienced users in many different organizations that deal with information technology, particularly for working with big data. Until now, however, the potential of such initiatives to assist in the assimilation of Docker technology by the bioinformatics community has never been considered. Currently, both the Panamax [20] and Shipyard [21] projects have been discontinued, rendering Rancher [22] and Portainer [23] the only alternatives available for the development of a bioinformatics-dedicated Docker management platform.

Rancher [22] is a robust software program for management of Docker systems and is widely used in datacenter environments and other complex computing ecosystems. It provides a platform for deployment of Docker infrastructures in an easy and controlled way by enabling the creation of a private platform for the implementation and administration of containers, using a web interface. However, Rancher [22] installation leads to creation of a series of parallel containers in the host machine because it employs Kubernetes as the major orchestrator of the Docker environment, consuming considerable amounts of computational resources. Portainer [23], on the other hand, requires only 1 container running in the host machine, reducing resource consumption, as well as the complexity inherent to its installation, maintenance, and use. In addition, Portainer [23] is used by Rancher as the default administration interface for Swarm cluster environments, adding yet another layer of complexity in using Rancher [22].

Thus, Portainer [23] was chosen as the basic platform for the development of the BioPortainer Workbench [26] because it provides a more suitable platform to accommodate the needs of the bioinformatics community, which includes a significant number of inexperienced users, sometimes working in research facilities with limited computational resources. However, the scope of the BioPortainer Workbench [26] surpasses the scope of Portainer [23] because it is not only focused on providing users with an easy-to-use graphic interface to assist in implementation and administration of Docker resources. In fact, the BioPortainer Workbench [26] provides GUIs that assist users in all steps of computational analyses, including (i) implementation of numerous bioinformatics software programs within Docker containers (which can be accomplished through a series of alternative platforms), (ii) management of computational resources made available to run such containers, and (iii) launching of bioinformatics applications, with various degrees of complexity, through a set of unique tools (not originally present in Portainer [23], such as the Job Runner, GUI Runner, and Pipeline Runner). Moreover, the BioPortainer Workbench [26] presents a series of unique computational resources, when compared to Portainer [23], such as the possibility of running GPU-accelerated applications (with the aid of the NVIDIA-Docker [33] plug-in) and the implementation of Docker-in-Docker environments, allowing additional containerization of processes, thus improving safety and management of resources. Finally, the BioPortainer Workbench [26] also offers unique resources that help to ensure replicability and reproducibility of data analysis (a major concern in bioinformatics research) by allowing the exchange of detailed protocols (with the aid of the Jupyter Notebook [28]) and executions (encapsulated in Docker images, with the aid of the BioPortainer Job Runner).

Thus, the BioPortainer Workbench [26] represents a pioneering effort in developing a highly comprehensive and easy-to-use Docker platform focused on bioinformatics, which may greatly assist in the dissemination of Docker virtualization technology among laboratories, contributing to improving the replicability and reproducibility of results in this complex field of research.

## Availability of supporting source code and requirements

- Project name: BioPortainer project
- Site: https://github.com/BioPortainer/BioPortainer
- Archive: http://doi.org/10.5281/zenodo.2377428
- RRID: SCR_017058
- Operating system(s): platform independent
- Programming language: Go, Python
- Other requirements: Docker, Docker Compose
- License: MIT

## Additional files

**Supplementary File 1:** The BioPortainer Workbench User Manual v1.0

## Abbreviations

CLI: command line interface; CPU: central processing unit; GPU: graphical processing unit; GUI: graphical user interface; JSON: JavaScript Object Notation; PaaS: platform as a service; RNA-seq: RNA sequencing; SaaS: software as a service.

## Competing interests

The authors declare that they have no competing interests.

## Funding

This work was supported by grants from Fundação de Amparo à Pesquisa do Estado de São Paulo (FAPESP) Nos. 17/13197-8 and 17/08112-3. F.B.M., D.A.B., and M.M.N. are recipients of scholarship grants from Coordenação de Aperfeiçoamento de Pessoal de Nível Superior (CAPES), while R.S.G. is the recipient of a scholarship grant from Conselho Nacional de Desenvolvimento Científico e Tecnológico (CNPq).

## Authors' contributions

F.B.M. conceived and developed the software; D.A.B., R.S.G., and M.M.N. developed and tested the JSON files for the BioPortainer GUI Runner and assisted in testing the software under different circumstances; D.L.J., R.C.O., and L.R.N. supervised the study and wrote the manuscript.

## Supplementary Material

GIGA-D-18-00229_Original_Submission.pdf

GIGA-D-18-00229_Revision_1.pdf

GIGA-D-18-00229_Revision_2.pdf

Response_to_Reviewer_Comments_Original_Submission.pdf

Response_to_Reviewer_Comments_Revision_1.pdf

Reviewer_1_Report_Original_Submission -- Konstantinos Krampis, PhD 7/29/2018 Reviewed

Reviewer_1_Report_Revision_1 -- Konstantinos Krampis, PhD 2/12/2019 Reviewed

Reviewer_2_Report_Original_Submission -- Gong Chen 7/29/2018 Reviewed

Reviewer_2_Report_Revision_1 -- Gong Chen 3/2/2019 Reviewed

Reviewer_3_Report_Original_Submission -- Ka Yee Yeung, Ph.D. 8/18/2018 Reviewed

Supplemental File
