# Supplementary material for: Bioportainer Workbench: a versatile and user-friendly system that integrates implementation, management, and use of bioinformatics resources in Docker environments
Source: Gigascience. 2019 Apr 25;8(4):giz041. doi: 10.1093/gigascience/giz041 (PMC6482343; doi:10.1093/gigascience/giz041)
Supplement: GIGA-D-18-00229_Original_Submission.pdf [file giz041_giga-d-18-00229_original_submission.pdf]

GigaScience

BioPortainer: a Portainer fork, carrying templates for lightweight graphic management of bioinformatics-related Docker environments

--Manuscript Draft--

**Manuscript Number:**	GIGA-D-18-00229
**Full Title:**	BioPortainer: a Portainer fork, carrying templates for lightweight graphic management of bioinformatics-related Docker environments
**Article Type:**	Technical Note
**Funding Information:**	FAPESP (#2017/13197-8) — Mr Luiz Roberto Nunes<br>FAPESP (#2017/08112-3) — Mrs Daniela Leite Jabes<br>CAPES — Mr Fabiano Bezerra Menegidio, Mr David Aciole Barbosa
**Abstract:**	Background: the Docker project is providing a promising strategy for the development of virtualization systems in bioinformatics. However, implementation of Docker containers is not entirely trivial for users not fully familiarized with Linux command lines, which has prompted the development of graphic interfaces to facilitate the installation and administration of Docker environments, such as the Portainer project. Results: this manuscript describes BioPortainer, a Portainer fork specifically designed for bioinformatics-related Docker applications. Bioportainer can be easily implemented, through deployment of a single Docker image, providing access to more than 60 pre-configured templates, available from BioPortainer´s unique repository. Conclusions: Bioportainer represents a pioneering effort to provide users with a graphic orchestrator of Docker environments, capable of assisting users through a straightforward and intuitive process for installation, configuration and management of a great variety of Docker-based bioinformatics tools, either in Docker Hosts or in Swarm Clusters.
**Corresponding Author:**	Daniela Leite Jabes, Ph.D<br>Universidade de Mogi das Cruzes<br>MOGI DAS CRUZES, São Paulo BRAZIL
**Corresponding Author Secondary Information:**	
**Corresponding Author's Institution:**	Universidade de Mogi das Cruzes
**Corresponding Author's Secondary Institution:**	
**First Author:**	Fabiano Bezerra Menegidio
**First Author Secondary Information:**	
**Order of Authors:**	Fabiano Bezerra Menegidio<br>David Aciole Barbosa<br>Daniela Leite Jabes<br>Regina Costa de Oliveira<br>Luiz Roberto Nunes
**Order of Authors Secondary Information:**	
**Additional Information:**	
**Question**	**Response**
Are you submitting this manuscript to a special series or article collection?	No
**Experimental design and statistics**	Yes

Powered by Editorial Manager® and ProduXion Manager® from Aries Systems Corporation

Full details of the experimental design and statistical methods used should be given in the Methods section, as detailed in our Minimum Standards Reporting Checklist. Information essential to interpreting the data presented should be made available in the figure legends.<br><br>Have you included all the information requested in your manuscript?	
**Resources**<br><br>A description of all resources used, including antibodies, cell lines, animals and software tools, with enough information to allow them to be uniquely identified, should be included in the Methods section. Authors are strongly encouraged to cite Research Resource Identifiers (RRIDs) for antibodies, model organisms and tools, where possible.<br><br>Have you included the information requested as detailed in our Minimum Standards Reporting Checklist?	Yes
**Availability of data and materials**<br><br>All datasets and code on which the conclusions of the paper rely must be either included in your submission or deposited in publicly available repositories (where available and ethically appropriate), referencing such data using a unique identifier in the references and in the "Availability of Data and Materials" section of your manuscript.<br><br>Have you have met the above requirement as detailed in our Minimum Standards Reporting Checklist?	Yes

Manuscript
Click here to download Manuscript Reference_PDF.pdf

GigaScience, 2018, 1–6

doi: xx.xxxx/xxxx
Manuscript in Preparation
Technical Note

TECHNICAL NOTE

BioPortainer: a Portainer fork, carrying templates for lightweight graphic management of bioinformatics-related Docker environments

Fabiano B. Menegidio[1,*], David A. Barbosa[1], Daniela L. Jabes[1], Regina Costa de Oliveira[1] and Luiz R. Nunes[2,*]

[1]Núcleo Integrado de Biotecnologia, Universidade de Mogi das Cruzes (UMC), Mogi das Cruzes, SP - 08780-911, Brazil. and [2]Centro de Ciências Naturais e Humanas, Universidade Federal do ABC (UFABC), Alameda da Universidade, s/n, São Bernardo do Campo, SP - 09606-045, Brazil.

*fabiano.menegidio@biology.bio.br; Luiz.nunes@ufabc.edu.br

Abstract

Background: the Docker project is providing a promising strategy for the development of virtualization systems in bioinformatics. However, implementation of Docker containers is not entirely trivial for users not fully familiarized with Linux command lines, which has prompted the development of graphic interfaces to facilitate the installation and administration of Docker environments, such as the Portainer project. **Results**: this manuscript describes BioPortainer, a Portainer fork specifically designed for bioinformatics-related Docker applications. BioPortainer can be easily implemented, through deployment of a single Docker image, providing access to more than 60 pre-configured templates, available from BioPortainers unique repository. **Conclusions**: BioPortainer represents a pioneering effort to provide users with a graphic orchestrator of Docker environments, capable of assisting users through a straightforward and intuitive process for installation, configuration and management of a great variety of Docker-based bioinformatics tools, either in Docker Hosts or in Swarm Clusters.

Key words: Docker; Bioinformatics; Management UI

Background

The increasing use of computational methods for biological data analysis has revolutionized the study of biology in recent decades. However, demands for expensive high-performance hardware to run such analyses and the complexity associated with many software installations often represent major challenges to the widespread use of such resources among researches. Thus, server-based cloud computing and virtualization systems have been extensively used to minimize these problems. As a consequence, concepts of Platform-as-a-Service (PaaS), provided by companies such as Google Genomics [1], or Amazon AWS Genomics [2], as well as Software-as-a-Service (SaaS), provided by initiatives, such as the Galaxy Project [3, 4, 5] and Cloudman [6], are being increasingly adopted in research organizations with little or no bioinformatics capabilities, as well as in biotech and pharmaceutical companies worldwide, as a strategy to reduce costs and avoid the problems associated with installing and maintaining their own bioinformatics facilities [7]. Up to now, most of these bioinformatics-related PaaS/SaaS are based on Virtual Machines (VM), which constitute a robust strategy to develop virtualization systems, but have the drawback of consuming large amounts of disk space and display low scalability, as well as difficulties pertaining their implementation in association with high-performance computing platforms. However, emergence of the Docker Project [8] is providing a new and promising virtualization strategy that consumes considerably less disk

Compiled on: June 20, 2018.
Draft manuscript prepared by the author.

space and provides the advantage of being platform-agnostic, since they rely on the configuration of containers, which can be consistently interchanged and deployed on different computing environments, regardless the specificities of their hardware and/or operating system, ensuring replicability and reproducibility of data analyses across different research facilities.

Thus, several bioinformatics-related PaaS and SaaS initiatives, based on Docker virtualization systems, have been recently developed, such as BioShaDock [9], AlgoRUN [10], GUIdock [11], Dockstore [12] and Biocontainers [13], among others. Even the Galaxy Project has recently incorporated Docker technology to allow local installation of Galaxy containers [14] via Docker-based virtualization systems. More recently, this Bioinformatics as a Service platform has been expanded by the development of Dugong, which introduced the concept of Desktop-as-a-Service (DaaS) in bioinformatics analyses [15]. However, in spite of the advantages provided by Docker and other container-based virtualization systems, they are not easily implemented by inexperienced users, mostly due to poor familiarity with the Docker Engine computational environment (based on a command-line interface), lack of proper documentation for many Docker applications and widespread availability of non-standardized images, built upon Dockerfiles containing obscure implementation steps. Moreover, the complexity of Docker systems tends to increase when environments are composed by different containers, or involve the implementation of Swarm, a native Docker tool that allows creation of clusters, composed by different Docker hosts within the same resource pool [16, 17].

To overcome such difficulties, different initiatives, such as Panamax [18], Shipyard [19], Rancher [20], and Portainer [21], sought the development of graphical interfaces aimed at assisting the implementation, administration and management of Docker environments by inexperienced users, under different computing ecosystems.. However, none of these systems has been employed, so far, to assist researchers to access and use the many Docker-based bioinformatics tools currently available. In this scenario, the present manuscript describes the development of BioPortainer [22], a fork of the original Portainer project that provides users with a series of preconfigured templates that allow fast and simple installation/administration of more than 60 Docker-based bioinformatics tools, which can be used in a comprehensive range of scientific applications.

Methods

BioPortainer Architecture

BioPortainer [22] is a free and open-source fork of Portainer, specifically targeted to the bioinformatics community. It was developed through a series of alterations in the original Portainer source code and conceived using a simple architecture scheme, based on three main components (Figure 1). The first structural component is a Docker Compose file (carrying all the necessary settings for complete operation of the Portainer management tool), while the second is a Dockerfile file (containing data for construction of Docker images). The third component is a JavaScript Object Notation (JSON) template file, which provides the interface between Docker images and the BioPortainer User Interface (UI). Although considered easy to build and implement, when compared to other files with the same objective and purpose, the JSON model [23] can become exceedingly complex for inexperienced users, since its format is based on a subset of the JavaScrip programming language. To overcome this difficulty, BioPortainer is based on a specifically designed JSON file that allows the utilization of preconfigured templates for a variety of bioinformatics-related Docker images through the Portainer UI, where they can be easily configured and deployed, for future use in a variety of experimental analyses.

BioPortainer installation and configuration

Installing BioPortainer is extremely simple and can be done in two ways, using either Docker, or Docker Compose.

If user has Docker installed in his/her operating system, only two steps are required to start a BioPortainer container, with direct access to its preconfigured template repository. In the first step, the BioPortainer image is downloaded from the Docker Hub server to the host machine, and, in the second, a container is created in the host, with the default installation of BioPortainer. When Linux is the OS of the host machine, the following commands must be executed on the terminal:

```
$ docker pull bioportainer/bioportainer
$ docker run -d -p 9000:9000 --name BioPortainer \
      -v /var/run/docker.sock:/var/run/docker.sock \
      -v ${PWD}/bioportainer_data:/data \
      bioportainer/bioportainer
```

Alternatively, BioPortainer can be installed directly through Docker Compose, by downloading Docker-Compose.yml and running Docker Compose:

```
$ wget http://bioportainer.ml/docker-compose.yml \
      -P bioportainer
$ cd bioportainer
$ docker-compose up -d
```

Currently, BioPortainer does not require any additional configurations for its full operation, thanks to alterations introduced in the Portainer original source code, resulting in direct installation of BioPortainer templates, in detriment of the templates available through the Portainer default configuration. However, if users already have a default installation of Portainer in their computing environment, the BioPortainer repository can also be deployed through the main menu, using the command: Settings → Application Templates → Use Custom Templates. Further details can be found in additional specific documentation available on the Portainer website [24].

After deployment, BioPortainer can be accessed through the host machine's address and port 9000 (if it's a local machine, use http://localhost:9000). Different modules that make up the BioPortainer graphical interface can be seen in the supplementary material that accompanies this manuscript. For example, the BioPortainer Dashboard (shown as Supplementary Figure 1) provides quick and general information about the managed host (either local host or Swarm Cluster), such as version of the installed Docker engine, amount of memory and CPU available and the number of containers, images, volumes and networks available; it also provides details on the use of resources by each container, image and network, allowing user to optimize the distribution of available resources among different applications. The BioPortainer App Templates option (shown as Supplementary Figure 2) displays a distinct format, when compared to the traditional Portainer installation, as it has been specifically reconfigured to provide access to the bioinformatics templates available at the BioPortainer repository (generated through BioPortainers unique JSON file). From this menu, users have access to intuitive forms that assist in installation and configuration of containers for specific bioinformatics tools, as shown in Supplementary Figure 3, which displays the options for installing and configuring the Dugong Clean CMD application from the BioPortainer repository. Finally, the Containers option (shown as Supplementary Figure 4) provides button interfaces that allow user to start/stop/restart and kill jobs using the available containers, along with other specific commands for their full management and administration.

Results

The BioPortainer template catalog

As mentioned above, BioPortainer (accessible via Github [22] and/or Bitbucket [25]) carries pre-configured templates for more than 60 Docker-based bioinformatics tools (see Supplementary Table 1), subdivided into 9 categories:

i. Galaxy Flavors: this category carries templates that enable installation of 25 versions of Galaxy images, carrying software for conducting a variety of Omics analyses, such as Transcriptomics, Phylogenenomics, Proteomics and Metagenomics, among others [3, 4, 5];
ii. Galaxy Tools: this category carries templates for the development and implementation of additional software into the main Galaxy instance, with the aid of Planemo [26], a command-line utility that helps to create and publish new Galaxy tools;
iii. BioContainers Flavors: this category contains a template to assist in the installation of the main Docker image of the BioContainers project, which allows installation and distribution of more than 2000 bioinformatics tools, within Docker containers, from the BioConda repository [13];
iv. Dugong Flavors: templates in this category assist in the implementation of the different versions of Dugong, a Docker implementation of the Desktop as a Service concept (DaaS), which provides a graphical user interface (GUI) that integrates access to Docker containers for more than 3500 bioinformatics-related software (available from BioConda, LinuxBrew and BioLinux repositories) and the Jupyter Notebook, to assist in reproducible exchange of data and protocols [15];
v. GUIdock Flavors: this category contains templates to assist in the installation of the different versions of GUIdock, a Docker image dedicated to providing graphic analytical tools (particularly suited for network analyses) within containers [11];
vi. Bioconductor Flavors: category containing templates for rapid implementation of bioinformatics tools for the R language environment, available from the Bioconductor repository [27];
vii. R Flavors [28] and (vii) RStudio [29]: categories containing templates for the installation of Docker images containing the R and Shiny languages, along with the standard installation Rstudio, providing a complete environment for statistical analysis of large datasets.
viii. Jupyter Notebook: a category containing templates to assist in the installation of standard Docker images for the Jupyter Notebook [30] within Docker containers. These images also allow implementation of the main tools/languages currently employed in bioinformatics protocols (such as Python, R, Scala, Spark, Mesos and Tensorflow, among others), which can be consistently exchanged among laboratories, thus contributing with replicability and reproducibility of data analyses.

All models provide custom options in their deployment forms and are specially designed to meet the needs of the different tools available, such as port mapping, volume mapping and network configurations, among others. An example of this can be seen in Supplementary Figure 5, which presents the implementation form of the Galaxy Stable template, containing configuration options for all the Magic Environment Variables required by Galaxy Stable.

Deploying and testing BioPortainer in alternative computing environments

BioPortainer can be configured to operate in a variety of computing environments. Supplementary File 1 provides detailed instructions on how to implement BioPortainer in both Docker engines and Swarm clusters and these instructions are complemented by step-by-step movie files, available at the BioPortainer Project webpage [22]. This website also provides testing environments for both types of installation, using three alternative software testing platforms: (i) Play-with-Docker [31], (ii) Katacoda Learning Platform [32] and (iii) Dply [33]. Once configured, BioPortainer can then be used to deploy containers to run a great variety of bioinformatics tools. Detailed instructions for container deployment are provided in the Supplementary file 2, using two examples: (i) a basic installation of Galaxy Stable and (ii) an installation of the DNA sequence aligner BWA, using Dugong [15]. These individual bioinformatics containers can also be tested with the aid of the software testing platforms available at the BioPortainer homepage (use of the Play-with-Docker platform is recommended, for simplicity).

Discussion

Bioinformatics lies in the intersection of biology, computer science and statistics and often attracts professionals with limited skills for the appropriate management of computational environments. Although several initiatives have recently demonstrated the viability of using Docker to provide bioinformatics tools to researchers, most of these Docker-based systems have been developed with little concern for inexperienced users, limiting their widespread implementation in research facilities. For example, Docker image repositories, like BioShaDock [9] and Dockstore [12] provide several bioinformatics software within Docker containers, but local installation of images still requires adjustments to ensure their full operation, such as the export of network service ports and configuration of data volumes, among other procedures, which are often unclear to the final user, due to lack of proper documentation in such repositories. Moreover, absence of proper standards for image generation and lack of curatorship led to the accumulation of heterogeneous tools in these repositories. Some of these problems were addressed by the development of standardized Docker images by BioContainers, which allows access to more than 2000 bioinformatics tools from the BioConda repository [13]. However, BioContainers operates exclusively through command lines, hampering its use amongst users not fully familiarized with Linux commands. Although future development of BioContainers may lead to its integration with the Galaxy graphical interface through Galaxy Interactive Environments (GIEs) [34], GIE deployment is not a trivial operation, since they have complex interactions with numerous services. Moreover, implementation of the Galaxy instance displays large requirements for memory and disk space and additional Galaxy tools have different requirements in computer memory, I/O speed, disk space, network bandwidth, density of computing cores, and parallel environment configurations, among other issues.

Thus, the development of specific tools, capable of assisting inexperienced users is of paramount importance to ensure the widespread use of Docker-based bioinformatics resources, which may greatly contribute to improve replicability and reproducibility of data analysis, given the platform-agnostic nature of Docker systems. In fact, the widespread use of Docker in different corporate business environments has been stimulated by initiatives, such as Panamax [18], Shipyard [19], Rancher [20], and Portainer [21], which developed graphical interfaces

to help in the implementation, administration and management of Docker environments by less experienced users in many different companies/organizations that deal with Information Technology (IT), particularly for working with Big Data. Until now, however, the potential of such initiatives to assist in the assimilation of Docker technology by the bioinformatics community has never been considered. Currently, both Panamax and Shipyard projects have been discontinued, rendering Rancher and Portainer as the only alternatives available for the development of a bioinformatics-dedicated Docker management platform.

Rancher is a robust software for management of Docker systems, widely employed in datacenter environments and other complex computing ecosystems. It provides a platform for deployment of Docker infrastructures in an easy and controlled way, by enabling the creation of a private platform for the implementation and administration of containers, using a web interface. However, Rancher installation leads to creation of a series of parallel containers in the host machine, since it employs Kubernetes as the major orchestrator of the Docker environment, consuming considerable amounts of computational resources. Portainer, on the other hand, requires only one container running on the host machine, reducing resource consumption, as well as the complexity inherent to its installation, maintenance and use. In addition, Portainer is used by Rancher as the default administration interface for Swarm cluster environments, adding yet another layer of complexity in using Rancher. Thus, by following the basic structure of the Portainer project, BioPortainer provides a more suitable platform to the less experienced bioinformatics community, representing a pioneering effort to the development of a bioinformatics-related Docker orchestrating platform, based on an easy-to-use graphical interface to assist in the configuration and use of some of the most comprehensive bioinformatics-related Docker systems. We expect that BioPortainer may greatly assist in the dissemination of Docker virtualization technology among bioinformatics laboratories, contributing to improve replicability and reproducibility of results in this complex field of research.

Availability of source code and requirements (optional, if code is present)

Lists the following:

- Project name: BioPortainer project
- Project home page: http://bioportainer.ml
- DOI: 10.5281/zenodo.1253032
- Operating system(s): Platform independent
- Programming language: Go
- Other requirements: Docker
- License: MIT

List of abbreviations

JSON, JavaScript Object Notation; PaaS, Platform-as-a-Service; SaaS, Software-as-a-Service; VM, Virtual Machine; DaaS, Desktop-as-a-Service; IT, Information Technology; GUI, Graphical user interface; CLI, Command Line Interface.

Consent for publication

Not applicable.

Competing Interests

The authors declare no competing interests.

Funding

This work was supported by grants from Fundação de Amparo à Pesquisa do Estado de São Paulo (FAPESP), grants #17/13197-8 and #17/08112-3. FBM and DAB are recipients of scholarship grants from the Brazilian Federal Agency CAPES.

Author's Contributions

FMB conceived and developed the software; DAB and DLJ assisted in testing the software under different circumstances; RCO and LRN supervised the study and wrote the manuscript.

Additional Files

- Supplementary Table 1: Preconfigured templates for Docker-based Bioinformatics tools available from the BioPortainer repository.
- Supplementary Figure 1: BioPortainer Dashboard.
- Supplementary Figure 2: BioPortainer App Templates menu.
- Supplementary Figure 3: Application form for the "Dugong CMD" template of the BioPortainer repository.
- Supplementary Figure 4: Option "Containers" from the BioPortainer menu.
- Supplementary Figure 5: configuration form for Galaxy Stable through BioPortainer.
- Supplementary File 1: Testing BioPortainer installation in different scenarios.
- Supplementary File 2: Deploying and testing Bioinformatics containers through BioPortainer.

References

1. Google Cloud, Google Genomics; 2018. https://cloud.google.com/genomics/, accessed 19 jun 2018.
2. Amazon, Amazon Web Services: Genomics in the Cloud; 2018. https://aws.amazon.com/health/genomics/, accessed 19 jun 2018.
3. Blankenberg D, Coraor N, Von Kuster G, Taylor J, Nekrutenko A. Integrating diverse databases into an unified analysis framework: a Galaxy approach. Database 2011;2011:1–9.
4. Afgan E, Baker D, van den Beek M, Blankenberg D, Bouvier D, Čech M, et al. The Galaxy platform for accessible, reproducible and collaborative biomedical analyses. Nucleic acids research 2016;44(w1):w3–w10.
5. Afgan E, Baker D, Batut B, vandenBeek M, Bouvier D, Čech M, et al. The Galaxy platform for accessible, reproducible and collaborative biomedical analyses: 2018 update. Nucleic Acids Research 2018;p. gky379.
6. Afgan E, Chapman B, Taylor J. CloudMan as a platform for tool, data, and analysis distribution. BMC Bioinformatics 2012 Nov;13(1):315.
7. AbdelBaky M, Parashar M, Kim H, Jordan KE, Sachdeva V, Sexton J, et al. Enabling High-Performance Computing as a Service. Computer 2012;45(10):72–80.
8. Docker Project, Docker; 2018. https://www.docker.com, accessed 19 jun 2018.
9. Moreews F, Sallou O, Ménager H. BioShaDock: a commu-

nity driven bioinformatics shared Docker-based tools registry. F1000Research 2015;4.
10. Hosny A, Vera-Licona P, Laubenbacher R, Favre T. AlgoRun: a Docker-based packaging system for platform-agnostic implemented algorithms. Bioinformatics 2016;32(15):2396–2398.
11. Hung LH, Kristiyanto D, Lee SB, Yeung KY. Guidock: using docker containers with a common graphics user interface to address the reproducibility of research. PloS one 2016;11(4):e0152686.
12. O'Connor BD, Yuen D, Chung V, Duncan AG, Liu XK, Patricia J, et al. The Dockstore: enabling modular, community-focused sharing of Docker-based genomics tools and workflows. F1000Research 2017;6.
13. da Veiga Leprevost F, Grüning B, Alves Aflitos S, Röst H, Uszkoreit J, Barsnes H, et al. BioContainers: an open-source and community-driven framework for software standardization. Bioinformatics 2017;33(16):580–2582.
14. Galaxy Project, Galaxy Containers; 2018. https://docs.galaxyproject.org/en/master/admin/special_topics/mulled_containers.html, accessed 19 jun 2018.
15. Menegidio FB, Jabes DL, Costa de Oliveira R, Nunes LR. Dugong: a Docker image, based on Ubuntu Linux, focused on reproducibility and replicability for bioinformatics analyses. Bioinformatics 2018;34(3):514–515.
16. Naik N. Applying Computational Intelligence for enhancing the dependability of multi-cloud systems using Docker Swarm. Computational Intelligence (SSCI), 2016 IEEE Symposium Series on 2016;p. 1–7.
17. Huang CH, Lee CR. Enhancing the Availability of Docker Swarm Using Checkpoint-and-Restore. Pervasive Systems, Algorithms and Networks, 2017 11th International Conference on Frontier of Computer Science and Technology, 2017 Third International Symposium of Creative Computing (ISPAN-FCST-ISCC), 2017 14th International Symposium on 2017;p. 357–362.
18. CenturyLink Labs, Panamax-UI; 2018. https://github.com/CenturyLinkLabs/panamax-ui, accessed 19 jun 2018.
19. Shipyard Project, Shipyard: Composable Docker Management; 2018. https://github.com/shipyard/shipyard, accessed 19 jun 2018.
20. Rancher Labs, Rancher Labs: Your Enterprise Kubernetes Platform; 2018. https://rancher.com/, accessed 19 jun 2018.
21. Portainer Project, Portainer: Simple management UI for Docker; 2018. https://github.com/portainer/portainer, accessed 19 jun 2018.
22. Menegidio FB, BioPortainer; 2018. https://github.com/LaBiOS/BioPortainer, accessed 19 jun 2018.
23. JSON ORG, JSON Manual; 2018. https://www.json.org/, accessed 19 jun 2018.
24. Portainer Project, Portainer Documentation; 2018. https://portainer.readthedocs.io/en/stable/, accessed 19 jun 2018.
25. Menegidio FB, BioPortainer Bitbucket; 2018. https://bitbucket.org/labios/bioportainer, accessed 19 jun 2018.
26. Galaxy Project, Planemo: Command-line utilities to assist in developing tools for the Galaxy Project; 2018. https://github.com/galaxyproject/planemo, accessed 19 jun 2018.
27. Gentleman RC, Carey VJ, Bates DM, Bolstad B, Dettling M, Dudoit S, et al. Bioconductor: open software development for computational biology and bioinformatics. Genome Biology (Online Edition) 2004;5:R80.
28. R Development Core Team, R: A Language and Environment for Statistical Computing; 2012. http://www.R-project.org, accessed 19 jun 2018.
29. RStudio Team, RStudio: Integrated Development Environment for R. Boston, MA; 2015. http://www.rstudio.com/, accessed 19 jun 2018.
30. Jupyter Project, Jupyter Project; 2018. http://jupyter.org, accessed 19 jun 2018.
31. Play with Docker, Play-with-Docker; 2018. https://labs.play-with-docker.com, accessed 19 jun 2018.
32. Ocelot Uproar Ltd, Katacoda - Interactive Learning Platform for Software Engineers; 2018. https://www.katacoda.com/menegidio, accessed 19 jun 2018.
33. Dply, Dply Server; 2018. https://dply.co, accessed 19 jun 2018.
34. Grüning BA, Rasche E, Rebolledo-Jaramillo B, Eberhard C, Houwaart T, Chilton J, et al. Jupyter and Galaxy: Easing entry barriers into complex data analyses for biomedical researchers. PLOS Computational Biology 2017 05;13:1–10.

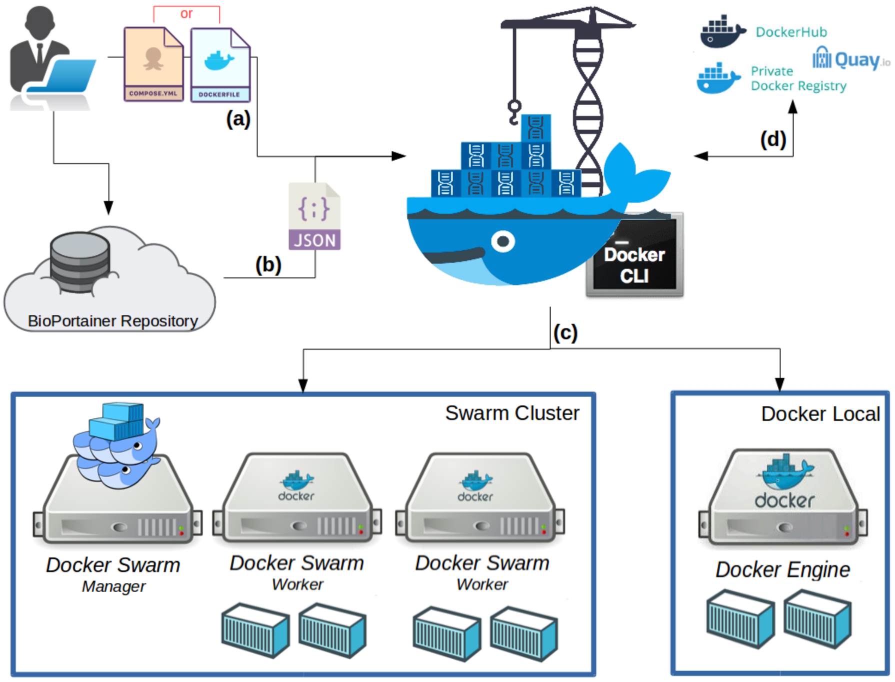

Figure 1. Overview of the BioPortainer Architecture: Users (a) can deploy more than 60 preconfigured bioinformatics templates available in the BioPortainer repository (b), as well as being able to administer and deploy Docker containers using images from the DockerHub, Quay.io or private repositories (d). BioPortainer provides a graphical interface to the Docker CLI (c), allowing full administration of Swarm clusters (including manager and workers), as well as local environments with only the Docker Engine and Docker Daemon. BioPortainer Dockerfile, as well as the JSON file of the BioPortainer repository, are freely available and users are encouraged to participate in its further development, by submitting pull requests, or by contributing new software to the repository.

Figure 1: Overview of the BioPortainer Architecture

Click here to download Figure Figure1.png

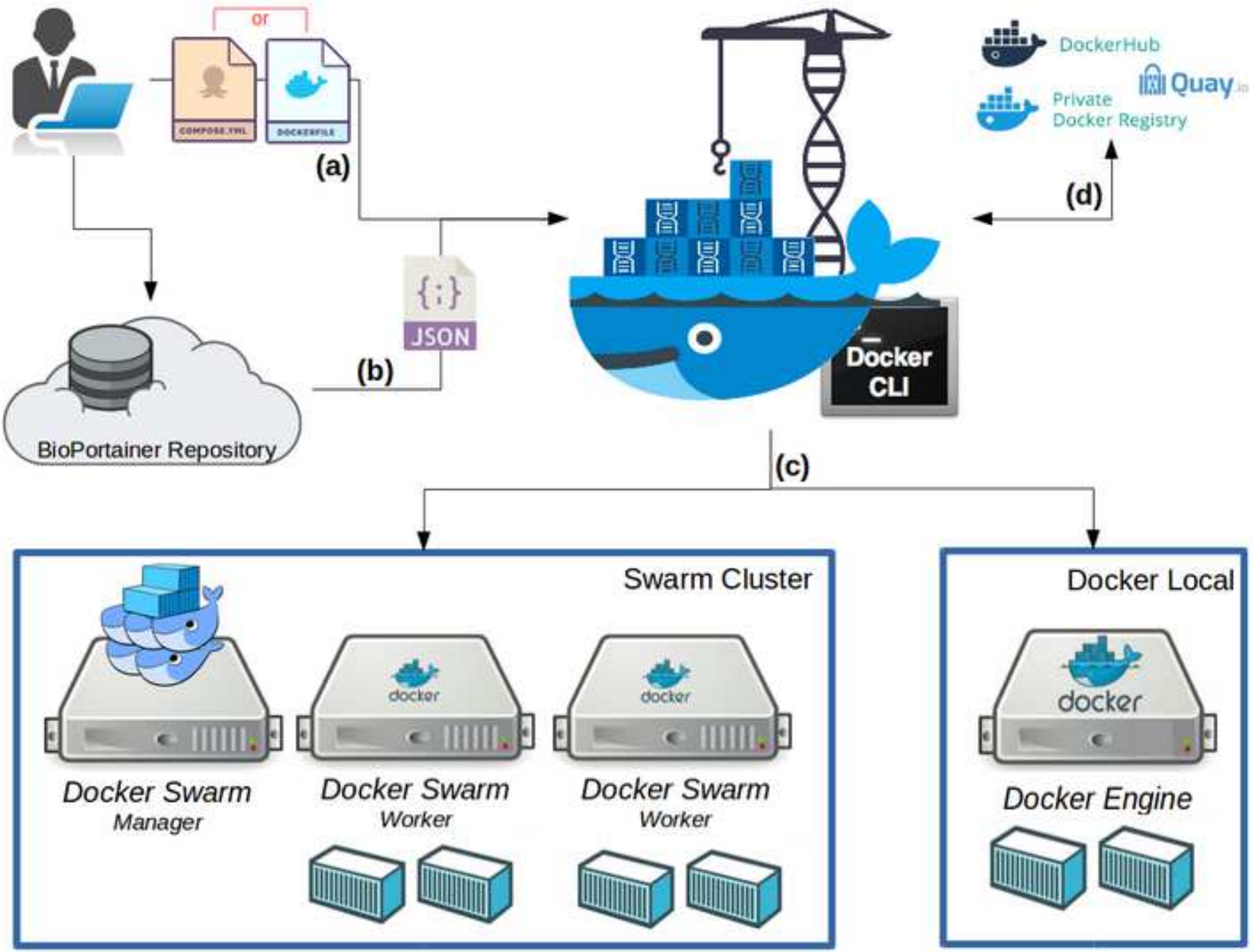

Click here to access/download
Supplementary Material
Supplementary Table 1.pdf

Supplementary Figure 1: BioPortainer Dashboar

Click here to access/download
Supplementary Material
Supplementary figure 1.pdf

Supplementary Figure 2: BioPortainer

Click here to access/download
Supplementary Material
Supplementary figure 2.pdf

Supplementary Figure 3: Application form for the Dugong CMD template of the BioPortainer repository

Click here to access/download
Supplementary Material
Supplementary figure 3.pdf

Supplementary Figure 4: Option "Container" from the BioPortainer menu

Click here to access/download
Supplementary Material
Supplementary figure 4.pdf

Supplementary Figure 5: Configuration form for Galaxy Stable through BioPortainer

Click here to access/download
Supplementary Material
Supplementary figure 5.pdf

Supplementary File 1: Testing BioPortainer installation in different scenarios

Click here to access/download
Supplementary Material
Supplementary File 1.pdf

Supplementary File 2: Deploying and testing Bioinformatics containers through BioPortainer

Click here to access/download
Supplementary Material
Supplementary File 2.pdf
