# Supplementary material for: Bioportainer Workbench: a versatile and user-friendly system that integrates implementation, management, and use of bioinformatics resources in Docker environments
Source: Gigascience. 2019 Apr 25;8(4):giz041. doi: 10.1093/gigascience/giz041 (PMC6482343; doi:10.1093/gigascience/giz041)
Supplement: GIGA-D-18-00229_Revision_2.pdf [file giz041_giga-d-18-00229_revision_2.pdf]

GigaScience

BIOPORTAINER WORKBENCH: A VERSATILE AND USER-FRIENDLY SYSTEM THAT INTEGRATES IMPLEMENTATION, MANAGEMENT AND USE OF BIOINFORMATICS RESOURCES IN DOCKER ENVIRONMENTS

--Manuscript Draft--

Manuscript Number:	GIGA-D-18-00229R2
Full Title:	BIOPORTAINER WORKBENCH: A VERSATILE AND USER-FRIENDLY SYSTEM THAT INTEGRATES IMPLEMENTATION, MANAGEMENT AND USE OF BIOINFORMATICS RESOURCES IN DOCKER ENVIRONMENTS
Article Type:	Technical Note
Funding Information:	FAPESP (#2017/08112-3) — Mrs Daniela Leite Jabes<br>FAPESP (#2017/13197-8) — Mr Luiz Roberto Nunes<br>CAPES — Mr Fabiano Bezerra Menegidio, Mr David Aciole Barbosa, Mr Marcio Morio Nishime<br>CNPq — Mr Rafael dos Santos Gonçalves
Abstract:	Background: the Docker project is providing a promising strategy for the development of virtualization systems in bioinformatics. However, implementation, management and launching of Docker containers is not entirely trivial for users not fully familiarized with command line interfaces (CLIs), which has prompted the development of graphic interfaces (GUIs) to facilitate the interaction of inexperienced users with Docker environments. Results: this manuscript describes the BioPortainer Workbench, an integrated Docker system that assists inexperienced users to interact with a bioinformatics-dedicated Docker environment at three main levels: (i) infrastructure, (ii) platform and (iii) application. Conclusions: the BioPortainer Workbench represents a pioneering effort in developing a highly comprehensive and easy-to use Docker platform focused on bioinformatics, which may greatly assist in the dissemination of Docker virtualization technology in this complex field of research.<br><br>Availability and implementation: Source code and instructions for local installation are available at https://github.com/BioPortainer/BioPortainer .
Corresponding Author:	Daniela Leite Jabes, Ph.D<br>Universidade de Mogi das Cruzes<br>MOGI DAS CRUZES, São Paulo BRAZIL
Corresponding Author Secondary Information:	
Corresponding Author's Institution:	Universidade de Mogi das Cruzes
Corresponding Author's Secondary Institution:	
First Author:	Fabiano Bezerra Menegidio
First Author Secondary Information:	
Order of Authors:	Fabiano Bezerra Menegidio
	David Aciole Barbosa
	Rafael dos Santos Gonçalves
	Marcio Morio Nishime
	Daniela Leite Jabes
	Regina Costa de Oliveira

Powered by Editorial Manager® and ProduXion Manager® from Aries Systems Corporation

	Luiz Roberto Nunes
**Order of Authors Secondary Information:**	
**Response to Reviewers:**	Response to Reviewers<br>Reviewer reports:<br>Reviewer #1: The authors have addressed all the comments by the reviewer satisfactorily, and the manuscript is recommended for acceptance publication.<br><br>Answer: Dear Reviewer. Thank you for your comments and suggestions, during the first round of peer review, which helped us to significantly improve the manuscript.<br><br><br><br>Reviewer #2: By trying out the new version of BioPortainer (now called BioPortainer Workbench),it is achieve these goals that easy to use Docker platform focused on bioinformatics, and allowing running program in any type of computing ecosystem,that help to ensure replicability and reproducibility of data analysis to researchers.Moreover, the BioPortainer Workbench also presents a series of unique computational resources,such as the possibility of running GPU-accelerated applications, the function of Docker-in-Docker environments and allowing the exchange of detailed protocols and executions with the aid of the Juyter Notebook, or unique BioPortainer Job Runner tool.<br>It's better to be consistent installation method of instructions document of https://hub.docker.com/r/bioportainer/bioportainer-pipeline-runner，https://github.com/BioPortainer/BioPortaine and BioPortainer Workbench User Manual。<br><br>Answer: Dear Reviewer. We standardized the instructions for software installation described both in the main manuscript, as well as in the current version of the BioPortainer Workbench User Manual (which accompanies the manuscript as supplementary material).<br>Installation instructions contained at the following URLs have also been corrected and formatted to the same standard:<br>https://hub.docker.com/r/bioportainer/bioportainer-pipeline-runner<br>https://github.com/BioPortainer/BioPortainer<br>https://bioportainer.github.io/BioPortainer<br>We appreciate your careful and thorough revision.
**Additional Information:**	
**Question**	**Response**
Are you submitting this manuscript to a special series or article collection?	No
**Experimental design and statistics**<br><br>Full details of the experimental design and statistical methods used should be given in the Methods section, as detailed in our Minimum Standards Reporting Checklist. Information essential to interpreting the data presented should be made available in the figure legends.<br><br>Have you included all the information requested in your manuscript?	Yes
**Resources**	Yes

Powered by Editorial Manager® and ProduXion Manager® from Aries Systems Corporation

A description of all resources used, including antibodies, cell lines, animals and software tools, with enough information to allow them to be uniquely identified, should be included in the Methods section. Authors are strongly encouraged to cite Research Resource Identifiers (RRIDs) for antibodies, model organisms and tools, where possible.<br><br>Have you included the information requested as detailed in our Minimum Standards Reporting Checklist?	
**Availability of data and materials**<br><br>All datasets and code on which the conclusions of the paper rely must be either included in your submission or deposited in publicly available repositories (where available and ethically appropriate), referencing such data using a unique identifier in the references and in the "Availability of Data and Materials" section of your manuscript.<br><br>Have you have met the above requirement as detailed in our Minimum Standards Reporting Checklist?	Yes

Manuscript

Click here to view linked References

Click here to access/download;Manuscript;BioPortainer_Workbench_New

OXFORD (GIGA)^n SCIENCE

GigaScience, 2017, 1–9

doi: xx.xxxx/xxxx
Manuscript in Preparation
Technical Note

TECHNICAL NOTE

Bioportainer Workbench: a versatile and user-friendly system that integrates implementation, management and use of Bioinformatics resources in Docker environments

Fabiano B. Menegidio[1,*], David A. Barbosa[1], Rafael dos S. Gonçalves[1], Marcio M. Nishime[1], Daniela L. Jabes[1], Regina Costa de Oliveira[1] and Luiz R. Nunes[2,*]

[1]Núcleo Integrado de Biotecnologia, Universidade de Mogi das Cruzes (UMC), Mogi das Cruzes, SP - 08780-911, Brazil. and [2]Centro de Ciências Naturais e Humanas, Universidade Federal do ABC (UFABC), São Bernardo do Campo, SP - 09606-045, Brazil.

[*]**Correspondence address**: Centro de Ciências Naturais e Humanas, Universidade Federal do ABC (UFABC), Alameda da Universidade, s/n, São Bernardo do Campo, SP - 09606-045, Brazil, phone: (55)(11)4996-7960. Fabiano Menegidio, E-mail: fabiano.menegidio@biology.bio.br ORCID ID: https://orcid.org/0000-0002-4705-8352, Luiz Nunes, E-mail: luiz.nunes@ufabc.edu.br ORCID ID: https://orcid.org/0000-0001-9619-269X.
ORCID IDs:

Abstract

Background: the Docker project is providing a promising strategy for the development of virtualization systems in bioinformatics. However, implementation, management and launching of Docker containers is not entirely trivial for users not fully familiarized with *command line interfaces* (CLIs), which has prompted the development of *graphic user interfaces* (GUIs) to facilitate the interaction of inexperienced users with Docker environments. **Results:** this manuscript describes the BioPortainer Workbench, an integrated Docker system that assists inexperienced users to interact with a bioinformatics-dedicated Docker environment at three main levels: (i) *infrastructure*, (ii) *platform* and (iii) *application*. **Conclusions:** the BioPortainer Workbench represents a pioneering effort in developing a highly comprehensive and easy-to use Docker platform focused on bioinformatics, which may greatly assist in the dissemination of Docker virtualization technology in this complex field of research.

Key words: Docker; Bioinformatics; Management UI

Background

The increasing use of computational methods for biological data analysis has revolutionized the study of biology in recent decades. However, demands for expensive high-performance hardware to run such analyses and the complexity associated with many software installations often represent major challenges to the widespread use of such resources among researches. Thus, server-based cloud computing and virtualization systems have been extensively used to minimize these problems. As a consequence, concepts of *Platform-as-a-Service* (PaaS), provided by companies such as Google Genomics [1], Amazon AWS Genomics [2], or Microsoft Azure [3], as well as *Software-as-a-Service* (SaaS), provided by initiatives, such as the Galaxy Project [4] and Cloudman [5], are being increasingly adopted in research organizations with little or no bioin-

Compiled on: March 19, 2019.
Draft manuscript prepared by the author.

formatics capabilities, as well as in biotech and pharmaceutical companies worldwide, as a strategy to reduce costs and avoid the problems associated with installing and maintaining their own bioinformatics facilities [6]. Up to now, most of these bioinformatics-related PaaS/SaaS are based on *Virtual Machines* (VM), which constitute a robust strategy to develop virtualization systems, but have the drawback of consuming large amounts of disk space, display low scalability, and are difficult to implement in association with high-performance computing platforms. However, emergence of the Docker Project [7] is providing a new and promising virtualization strategy that consumes considerably less disk space and provides the advantage of being platform-agnostic, since they rely on the configuration of containers, which can be consistently interchanged and deployed on different computing environments, regardless the specificities of their hardware and/or operating system, which helps to ensure replicability and reproducibility of data analyses across different research facilities.

Thus, several bioinformatics-related PaaS and SaaS initiatives, based on Docker virtualization systems, have been recently developed, such as BioShaDock [8], AlgoRUN [9], GUIdock [10], Dockstore [11] and BioContainers [12], among others. Even the Galaxy Project [4] has recently incorporated Docker technology to allow local installation of Galaxy containers [13] via Docker-based virtualization systems. More recently, this *Bioinformatics-as-a-Service* platform has been expanded by the development of Dugong [14], which introduced the concept of *Desktop-as-a-Service* (DaaS) in bioinformatics analyses. Other projects, such as Snakemake [15], Common Workflow Language [16] and NextFlow [17] have provided frameworks for the implementation, administration and execution of complex pipelines and workflows within Docker containers, allowing simultaneous management and coordinated launching of the various software involved in such analyses.

However, in spite of the advantages provided by Docker and other container-based virtualization systems, they are not easily implemented by inexperienced users, mostly due to poor familiarity with the Docker Engine computational environment, based on a *command-line interface* (CLI), lack of proper documentation for many Docker applications and widespread availability of non-standardized images, built upon Dockerfiles containing obscure implementation steps. Moreover, the complexity of Docker systems tends to increase when environments are composed by different containers, or involve the implementation of Swarm, a native Docker tool that allows creation of clusters, composed by different Docker hosts within the same resource pool [18, 19].

To overcome such difficulties, different initiatives sought the development of *graphical user interfaces* (GUIs), aimed at helping inexperienced users to interact with Docker-environments, at different levels. For example, projects such as Panamax [20], Shipyard [21], Rancher [22], and Portainer [23] have provided GUIs to assist users at the infrastructure level, as they allow easy implementation and resource management of Docker environments.

The Galaxy Project [4], on the other hand, seeks to provide a platform based on a user-friendly web GUI for software launching, but its implementation is not a trivial action, since a simple Galaxy installation requires considerable disk space and complex installation steps for its full operation. Finally, projects like AlgoRUN [9] tried to facilitate the launching of CLI-based applications by the development of dedicated and customizable GUIs, but manual installation of individual software in their respective containers, without the help of a framework, such as Conda [24], and repositories such as Bioconda [25], may have impacted on its full adoption by the Bioinformatics community.

In this scenario, this manuscript describes the BioPortainer Workbench [26], an integrated Docker system that seeks to assist inexperienced users to interact with a bioinformatics-dedicated Docker environment at three main levels: in the *Infrastructure Layer*, the BioPortainer Workbench [26] provides a GUI, based on the Portainer project [23], which allows rapid and simple implementation/management of a full Docker ecosystem; in the *Platform Layer*, the software provides a wide range of intuitive template forms that assist users in the installation and configuration of containers, carrying specific bioinformatics tools, from a variety of alternative platforms (based on CLIs, GUIs, or on a virtual desktop); finally, in the *Application Layer*, the BioPortainer Workbench [26] provides a series of CLI-based and GUI-based interfaces to assist users in launching jobs with varying degrees of complexity (from single application analyses to complex pipelines and workflows).

BioPortainer Workbench Architecture

BioPortainer Workbench [26] (BioPortainer, RRID:SCR_017058) is an open-source software developed under the MIT license and designed in a modular way, aimed at facilitating user interaction with Docker environments in three different computational layers: (i) *infrastructure*, (ii) *platform* and (iii) *application*. Its basic structure is briefly described in Figure 1. To deploy the software, user must initially access the BioPortainer Workbench image (Figure 1b) and install it, either in a single Docker engine, or in a Swarm cluster (Figure 1c). Once installed, BioPortainer Workbench [26] (Figure 1d) consists of two basic containers: the *BioPortainer Panel* and the *BioPortainer Pipeline Runner* (Figure 1e). From a functional point of view (Figure 1f), the two containers offer a number of tools that allow users to perform a series of actions in the created Docker environment, such as: (i) managing Docker resources associated with the BioPortainer Workbench [26]; (ii) installing bioinformatics applications based on several platforms described in the literature and (iii) launching different types of analyses, using either command-line (CLI), or graphic-based interfaces (GUI). Such analyses may be conducted with the help of BioPortainer Workbench's own resources (Figure 1g), or with resources harnessed from external repositories, which provide preconfigured images, files, commands or scripts for the execution of bioinformatics software, with varying levels complexity (Figure 1h).

The BioPortainer Workbench image, as shown in Figure 1 (b), consists of a Docker Compose file, which is responsible for building the Docker environment in the host machine, using Dockerfile files, which are associated with the two main containers of the software: the *BioPortainer Panel* and the *BioPortainer Pipeline Runner*. The third component of the image is a template repository, containing a series of JSON files, responsible for building the templates associated with platform installation, as well as for building the GUI forms used to launch bioinformatics tools, with the aid of the *BioPortainer GUI Runner* (see details below).

The Docker Compose file contains all the necessary settings for complete system operation, including the execution settings of the two main containers (*BioPortainer Panel* and *BioPortainer Pipeline Runner*). In addition to these two main modules, there is a third module (not shown in Figure 1) called the *BioPortainer Watchtower*, designed to monitor the main containers during execution. This module also monitors the original BioPortainer Workbench images, updating the whole Docker environment, whenever new versions of the software are made available. The Docker Compose file can be easily edited with the aid of any text editor, so as to expand its functionality by incorporating additional features, such as web proxies and/or tools for Continuous Integration and Continuous Delivery (CI/CD),

such as Jenkins [27], for example. To assist in the incorporation of these new resources, a virtual network, called BioPortainer local, was created, in order to guarantee efficient communication between the containers. Thus, if users wish to incorporate new features into the BioPortainer Workbench [26], it is necessary to insert such features in this network, through the networks parameter, to guarantee their efficient integration to the different modules of the software.

The second component of the image are the Dockerfile files, which contain instructions for building the Docker images of the *BioPortainer Panel* and *BioPortainer Pipeline Runner* modules. These Dockerfiles are available through GitHub and can be easily expanded to accommodate additional needs of any user. The Dockerfile of the *BioPortainer Panel* module was developed from the original source file of Portainer [23], which has been modified to incorporate specific features of the BioPortainer Workbench [26], such as the tools *BioPortainer Job Runner* and *BioPortainer GUI Runner*. The Dockerfile of the *BioPortainer Pipeline Runner*, on the other hand, has been developed independently and presents a greater level of complexity in its structure, as it carries all the software and library prerequisites necessary for the execution of NextFlow scripts [17] in a *Docker-in-Docker* environment (DinD). In addition, given the characteristics of this complex environment, the *BioPortainer Supervisor* tool (not shown in Figure 1) has been added to this Dockerfile, providing users with a GUI (accessed through port 7000) that enables full management of the *BioPortainer Pipeline Runner* tool, the Jupyter Notebook [28] and the *Docker-in-Docker* environment. It also allows users to analyze execution logs and control startup, shutdown and restart of processes within containers.

The third component of the image is the BioPortainer Repository [26], consisting of a series of JSON files. The JSON (JavaScript Object Notation) [29] language allows storage of data structures in a standard interchange format, which can be used for transmitting data between a server and a graphical web interface application. One of these JSON files is responsible for generating the GUI templates that will assist users during the installation of the *BioPortainer Bioinformatics Platforms* (accessible through the *BioPortainer Panel* main menu). The BioPortainer Repository [26] also carries additional JSON files that provide users with GUIs containing the interactive forms that assist in the launching of bioinformatics tools through the *BioPortainer GUI Runner* (see below). All JSON files available at the BioPortainer Repository [26] were manually developed to ensure perfect adaptation to the specific environmental variables and parameter prerequisites of their specific platforms/-tools. Moreover, all of them were individually tested by expert curators and further evaluated by Continuous Integration and Continuous Delivery (CI/CD), using the tools TravisCI [30] and CircleCI [31]. Users interested in modifying the BioPortainer Workbench [26] JSON files, in order to expand their resources and/or adapt them to new needs, can obtain the individual files from the BioPortainer Workbench project webpage [26].

Installing and Configuring the BioPortainer Workbench

Installation of the BioPortainer Workbench [26] is extremely simple and requires only Docker and Docker Compose as initial requirements. After both components are installed, only two steps are needed to start a BioPortainer Workbench environment. In the first step, the compose.yml file is downloaded from the server (GitHub, or BioPortainer Workbench homepage [26]) to the BioPortainer folder, in the host machine. In the second step, the Docker Compose is executed, downloading the images for the standard BioPortainer Workbench modules and the service is started. When Linux is the host machine's operating system, the following commands must be run on the terminal:

```
$ wget https://goo.gl/bNecPA -O docker-compose.yml
$ docker-compose up -d
```

During the deployment process, some ports and disk volumes will be automatically configured in the host machine. Details on the ports and volumes created are available in the BioPortainer Workbench User Manual, which accompanies this manuscript as a supplementary file. In a standard implementation, the BioPortainer Workbench [26] will use the localhost address (IP 0.0.0.0) as the default address for its internal links. If access is not performed through a local network, additional settings must be made in the Compose file, according to Docker's official documentation [7].

In order to simulate and test the implementation, administration and operation of the BioPortainer Workbench [26], we emulated its deployment in a test environment, using the Play-with-Docker [32] testing platform. The installation processes, as well as the results from this test, can be viewed through video files, available at the BioPortainer Workbench project webpage [26]. These videos demonstrate that, once installed, BioPortainer Workbench [26] is fully functional and can be readily used for container implementation/administration, through the *BioPortainer Panel*, or for the execution of a variety of (simple or complex) bioinformatics analyses, using any of its various implementation tools, which will be described in the next section (see the BioPortainer Workbench User Manual for details).

Features and Functionalities of the BioPortainer Workbench

As mentioned above, the BioPortainer Workbench [26] aims not only at providing Bioinformatics tools in a platform-agnostic environment, such as Docker, allowing its implementation in any type of computing ecosystem, but also at delivering a friendly interface that allows users to interact with this environment in different functional layers: (i) *infrastructure*, (ii) *platform* and (iii) *application*. Thus, description of the features and functionalities of the BioPortainer Workbench [26] will follow this same rationale, during the next sections.

The Infrastructure Layer: Implementing and Managing a Docker Environment with the Aid of the BioPortainer Panel

The proper management of Docker resources is often a serious problem for inexperienced users, as it is usually performed through a *command-line-based interface* (CLI). However, such management is essential for the operation of the host system, since downloading images and creating volumes in a non-transparent way can lead, for example, to excessive consumption of disk space, leading to rapid degradation of the host machine's environment. In addition, inadequate management of disk volumes can increase the number of orphan volumes in the system, which will continue to occupy physical space even after removal of the containers to which they were linked.

To overcome such obstacles, the BioPortainer Workbench [26] provides a graphic interface to assist users in the management and distribution of such resources, through the *BioPortainer Panel* module, composed of different submodules, which can be seen in the BioPortainer Workbench User Manual. For example, the *BioPortainer Dashboard* (shown as Figure S4 in the manual) provides quick and general information about the managed host (either a single Docker host, or a Swarm cluster),

such as: (i) version of the installed Docker engine; (ii) amount of memory and CPU available; (iii) number of containers, images, volumes and networks available and (iv) details on the use of such resources by each container, image and network, allowing users to optimize their distribution among different applications. The *Containers* option (Figure S5 in the manual), on the other hand, provides button interfaces that allow user to start/stop/restart and kill jobs using the available containers, along with other specific commands for their full management and administration.

In addition to assisting in the optimal distribution of resources, the *BioPortainer Panel Dashboard* also allows the use of alternative runtimes. Among these runtimes, we highlight the possibility of using NVIDIA-Docker [33], a Docker Engine plug-in designed to facilitate the deployment of containers capable of using Graphical Processing Units (GPUs) as their main processing units. The use of this runtime can be very helpful while running jobs that require high processing capacity, which can be achieved through the use of GPUs, rather than the system's CPUs (which are traditionally used by most computer applications). To accomplish that, the plug-in automatically recognizes GPU devices in the host, as well as their drivers and volume-mounting points, directing the execution of container services to GPU drives, rather than CPUs. To further facilitate the use of GPU resources, the BioPortainer Workbench [26] provides a template platform, developed with the aid of CUDA [34] and CUDNN [35] libraries, ready to work with NVIDIA-Docker [33], enabling the creation, debugging, and performance optimization of NVIDIA-GPU-accelerated applications (see below). These templates enable complete integration between the Docker and GPU environments with both Conda [24] and PIP [36] package managers, as they have a set of tools developed in Python.

The Platform Layer: Implementing Containers Carrying Fully Functional Bioinformatics Tools with the Aid of the BioPortainer Bioinformatics Platforms

The installation of several bioinformatics tools can be done through alternative platforms, many of them extensively described in the literature [4, 9, 10, 12, 14]. Some of these platforms provide access to large software repositories such as Bioconda [25], LinuxBrew [37], or BioConductor [38], for example, which allow the installation of thousands of generic bioinformatics tools. On the other hand, there are also platforms dedicated to repositories focused on more specific applications, enabling the installation of languages (such as R, or Shiny), or accessories (such as the Jupyter Notebook [28]), for example, which can be integrated into different types of analyses (see below). Moreover, some of these platforms have specificities that differentiate them from one another, including for example, the use of interfaces based on CLI (BioContainers [12]), GUI (Galaxy [4]), or Virtual Desktop (Dugong [14]), providing users with a variety of alternatives for container creation.

The *BioPortainer Bioinformatics Platforms* option (Figure S7 in the manual) provides access to all *Bioinformatics Platform Templates* available at the *BioPortainer repository*. From this menu, users have access to intuitive template forms that assist in the installation and configuration of containers, carrying specific bioinformatics tools, using whichever platform they deem most convenient to their needs and/or expertise. As mentioned above, these templates are defined by a preconfigured JSON file, available from the BioPortainer Repository [26]. Using these templates, users can implement and configure containers carrying single tools, or a set of related tools (described in a single Compose file) in any local Docker engine. Although considered easy to build and implement, when compared to other files with the same purpose, the JSON format can become extremely complex for inexperienced users, as it is based on a subset of JavaScript programming language. Thus, the availability of preconfigured models minimizes problems associated with implementation and configuration of tools within containers and improve customization to the unique requirements of each platform/software to be implemented. Nonetheless, it is possible to add new templates and/or update/modify existing templates in an easy and intuitive way with the help of options such as add template and update template, available through the *BioPortainer Panel* main menu (see Figure S7 in the manual).

Currently, the *BioPortainer Bioinformatics Platforms* option provides 11 Docker-based bioinformatics platforms (see Table S1 in the manual), which allow access to a wide variety of tools that can be accessed, installed, managed and launched, with the aid of the BioPortainer Workbench [26]. These platforms include:

i. The **BioPortainer CPU Platform** provides pre-configured templates aimed at creating images/containers carrying bioinformatics tools combined with features that help to ensure reproducibility and replicability in data analysis. These include screen, GNU parallel, script/scriptreplay and Jupyter Notebook [28], among others. In addition, they allow users to install the Miniconda2 and Miniconda3 packages, enabling the deployment and use of more than 2000 bioinformatics tools, developed in Python, available through the Bioconda [25] repository.

ii. The **BioPortainer GPU Platform** is similar to the CPU Platform, but provides pre-configured templates aimed at creating images/containers with the help of the CUDA [34] and CUDNN [35] library kits. As a result, such containers can be processed through GPU units, using the NVIDIA-Docker [33] plug-in, as described above.

iii. The **BioPortainer GUI Runner Platform** allows users to implement containers to be launched with the aid of the BioPortainer GUI Runner. This tool (described in detail in the next section), allows users to launch over 100 bioinformatics applications through a user-friendly GUI, dismissing the use of CLI-based interfaces.

iv. The **Galaxy Platform** provides templates that allow the implementation of 25 versions of galaxy images, carrying software for performing a variety of Omics analyses such as transcriptomics, phylogenomics, proteomics and metagenomics, among others [4, 39, 40]. All tools available on this platform are fully adapted to work through the Galaxy interface and, thus, do not require the use of CLI-based interfaces. (the BioPortainer Workbench User Manual provides detailed instructions on how to access the Galaxy Stable user interface, after installing this tool in a Docker container).

v. The **Galaxy Tools Platform** features templates for the development and implementation of additional software on the main Galaxy instance [4], with the help of Planemo [41], a command-line utility that helps to create and publish new Docker-based Galaxy tools [4];

vi. The **BioContainers Platform** contains a template for assisting users to deploy the main Docker image of BioContainers [12], a CLI-based platform that allows installation and distribution of over 2000 bioinformatics tools (available from the Bioconda [25] repository), within Docker containers.

vii. The **Dugong Platform** provides templates that assist in the implementation of different versions of Dugong [14], a Docker-based virtual desktop that helps users to deploy Docker containers carrying over 3500 bioinformatics software (available from the Bioconda [25], LinuxBrew [37] and BioLinux [42] repositories), directly integrated with the Jupyter Notebook [28];

viii. The **GUIdock Platform** contains templates to assist in the

implementation of different versions of GUIdock, a Docker image dedicated to providing graphical analytical tools (particularly suitable for network analyses) inside containers [10];

ix. The **Bioconductor Platform** provides templates for the rapid implementation of images/containers carrying a variety of bioinformatics tools that can be used in the R language environment, available from the Bioconductor repository [38];

x. The **R and RStudio Platform** [43, 44] provide templates for the implementation of images/containers carrying the R and Shiny languages, along with the standard installation of Rstudio, providing a complete environment for running Bioconductor tools and/or performing statistical analyses of large datasets.

xi. The **Jupyter Notebook Platform** provides templates to assist in the implementation of standard Docker images/containers carrying the Jupyter Notebook [28], along with the main tools/languages currently employed in the development of bioinformatics protocols (such as Python, R, Scala, Spark, Mesos and Tensorflow, among others).

All templates offer custom options in their deployment forms and are specially designed to meet the needs of the different tools available, such as port/volume mapping and network configurations, among others. The BioPortainer Workbench User Manual provides detailed instructions for installing containers carrying bioinformatics tools from the *BioPortainer Bioinformatics Platforms*. Two examples are used to illustrate this process: (i) the installation of a container carrying the Galaxy Stable tool from the Galaxy Platform and (ii) the installation of a container carrying the Dugong Clean CMD from the Dugong Platform [14].

The Application Layer: Launching Bioinformatics Analyses from Docker Containers

Launching of Bioinformatics Analyses can be performed using 4 different tools, available from the BioPortainer Workbench [26]: (i) *BioPortainer Console*; (ii) *BioPortainer Job Runner*; (iii) *BioPortainer GUI Runner* and (iv) *BioPortainer Pipeline Runner*.

Launching Bioinformatics analyses with the aid of the BioPortainer Console

The *BioPortainer Console* represents the simplest alternative for launching simple analyses, normally involving a single bioinformatics tool. Once a container carrying a specific tool has been implemented, users only need to click the Console icon (>_), available from the *Containers* option of the *BioPortainer Panel* main menu (see Figure S2 in the manual), which will cause a CLI-based interface to pop-up. Next, users can simply enter the necessary commands to run the application at the bash of this CLI. This tool was ported directly from the Portainer project [23], and should be preferably used by users fully familiarized with Linux commands, who wish to save time during analyses, by avoiding the use of GUIs. The *BioPortainer Console* also provides a simple and fast interface for full interaction and administration of containers, without the need to install any external or internal tools, such as servers and clients for the SSH protocol. A detailed demonstration on how to use the *BioPortainer Console* to launch an alignment of RNA-seq data against a reference genome, using the BWA aligner is shown in the BioPortainer Workbench User Manual.

Launching Bioinformatics analyses with the aid of the BioPortainer Job Runner

Like the *BioPortainer Console*, the *BioPortainer Job Runner* is intended for users familiarized with Linux commands. However, while the former is more suited for running simple analyses, using individual tools/containers, the latter is configured to perform more complex analyses, involving various steps and software. This tool uses, as target, a preconfigured Docker image, containing all the tools necessary for the analysis in question. The *BioPortainer Job Runner* interface can be accessed through the *BioPortainer Panel* main menu and is shown in Figure S12 of the manual. To trigger this tool, users must initially provide the target Docker image (which may be present either in the host machine, or in external repositories, such as DockerHub). Next, users must provide an execution script, which can either be typed into the web-editor interface, or imported through the upload option (see Figure S12 in the manual). Finally, by clicking on *execute*, users will have the script executed within a container built from the selected image. At the end of the process, the job's output will also be present within this container, which can then be converted into a Docker image to be shared through public or private repositories, such as DockerHub or Quay.io. Thus, the BioPortainer Job Runner allows users to encapsulate entire executions within a single exchangeable Docker image/container, contributing to promote replicability and reproducibility of data analyses across laboratories (the entire process can also be tracked through *Job Runner's* own *Job History*). A detailed demonstration on how to use the *BioPortainer Job Runner* to launch a full differential expression analysis with RNA-seq data, using the Tuxedo Suite, is shown in the BioPortainer Workbench User Manual.

Launching Bioinformatics analyses with the aid of the BioPortainer GUI Runner

The *GUI Runner* is a specific tool of the BioPortainer Workbench [26] and was developed in Python 3.4 (or higher), Tornado 4 (or higher) and Typing. It allows users not fully familiarized with Linux commands to launch bioinformatics tools from their respective containers, with the help of intuitive GUIs. These interfaces are configured from specific JSON files, each of them specifically developed for a particular tool. Altogether, the BioPortainer Repository [26] contains preconfigured JSON files that enable the launching of 109 bioinformatics tools from the *GUI Runner*, which can be used for a wide range of Bioinformatics analyses, such as: quality control trimming of NGS sequence data, gene identification through Hidden Markov Models (HMM), computation of GC bias across genomes and many others (a complete list of tools that can be currently launched with the aid of the *BioPortainer GUI Runner* can be seen in Table S2, available in the BioPortainer Workbench User Manual).

The GUI Runner templates are arranged in two folders: (i) conf/runners/, which contains the JSON files used to build the GUIs, and (ii) conf/scripts/, which contains the execution scripts, which receive the variables typed in these GUIs prior to execution. As mentioned above, the current version of the BioPortainer Repository [26] contains GUI templates that allow 109 bioinformatics tools to be launched by the *BioPortainer GUI Runner*, but our team is continually working to increase this number in further releases of the Repository. Users interested in developing new JSON files to extend the scope of *GUI Runner* to new analytical tools can find instructions on how to download a basic JSON model in the BioPortainer Workbench User Manual. It is important to note, however, that new JSON files will only become functional after being transferred to the conf/runners/ folder. Moreover, each JSON file must be unique and

represent a particular script or tool. Thus, to launch a container containing two different tools, users must develop a specific JSON file for each tool.

Finally, access to the *BioPortainer GUI Runner* can be achieved in two ways: (i) through the *GUI Runner* option, available from the *BioPortainer Panel Bioinformatics Platforms* menu (see Figure S7 in the manual), or by clicking on port 5000 of the container in question, through the *Containers* option (see manual, for details). A detailed demonstration on how to use the *BioPortainer GUI Runner* to launch a FASTQC analysis from an NGS dataset is shown in the BioPortainer Workbench User Manual.

Launching Bioinformatics analyses with the aid of the BioPortainer Pipeline Runner

The *Bioportainer Pipeline Runner* is dedicated to helping inexperienced users to conduct complex analyses, involving multiple bioinformatics tools, connected through pipelines and/or workflows, in a Docker ecosystem. Although implemented in a separate container, all its features, including volumes and environment variables, are also managed and monitored through the *Bioportainer Panel*.

The *BioPortainer Pipeline Runner* is accessed through the *BioPortainer Panel* main menu (see Figure S3 in the manual), providing users with a graphical interface that allows the download, through a GIT protocol, of bioinformatics pipelines available at the NextFlow and NF-Core repositories. Both repositories are developed by collaborative projects that employ NextFlow [17] (a type of Domain-Specific Language) to develop and adapt scalable and reproducible scientific workflows, using software containers. Thus, several pipelines developed by these projects include scripts and images specifically developed for complex bioinformatics analyses, such as: (i) 16S rRNA amplicon sequence analysis using QIIME, (ii) identification and quantification of peptides from mass spectrometry raw data, (iii) HLA typing from next-generation sequencing data and (iv) Chromatin Immunoprecipitation (ChIP-seq) analysis, among many others. Overall, more than 80 high-quality bioinformatics pipelines are available from the NextFlow and NF-Core repositories. Once activated, the *BioPortainer Pipeline Runner GUI* provides different user options through its menu, which allow users to download, execute, monitor, update and delete NextFlow-based pipelines from the abovementioned repositories, or harnessed from alternative Github sources (details regarding the options to run the *BioPortainer Pipeline Runner* can be seen in the BioPortainer Workbench User Manual).

The incorporation of NextFlow [17] as the main framework for the *Bioportainer Pipeline Runner* was facilitated by its easy integration with the Docker project. Moreover, the NextFlow [17] community is extremely active, which should help to expand the number and scope of available pipelines over the next years. In this sense, we understand that the BioPortainer Workbench [26] may contribute to foster the utilization of such pipelines by inexperienced users.

Alternatively, users may choose to launch their analyses through the *BioPortainer Pipeline Runner* using a protocol previously implemented in the Jupyter Notebook [28]. To accommodate this possibility, the BioPortainer Workbench [26] provides a functional installation of the Jupyter Notebook [28], in conjunction with the Python3 kernel and the Bash kernel. By using such resources, the *BioPortainer Pipeline Runner* becomes an extremely flexible tool, allowing the construction an execution of complex pipelines, using libraries and tools developed in both NextFlow [17] and Python languages. Moreover, notebooks created through this approach can be shared among laboratories, ensuring replicability of the entire computing environment used for data analysis. Implementation of the Jupyter Notebook [28] is linked to a token that is automatically generated during startup of the Docker engine and detailed instructions for connecting it to the *BioPortainer Pipeline Runner* can be seen in the BioPortainer Workbench User Manual.

To demonstrate the full functionality of the *BioPortainer Pipeline Runner*, we ran two pipelines obtained from the NextFlow [17] project homepage. One of them (the *rnatoy* pipeline) has been designed to conduct a workflow for RNA-seq analysis using Tuxedo Suite. The second pipeline (named *nmdp-flow*) performs Variant Calling analyses with RNA-Seq data and is based on the GATK (Genome Analisys Toolkit) best practices, developed by the Broad Institute (click here for details). Once again, detailed demonstrations on how to launch these pipelines (using both the *BioPortainer Pipeline Runner GUI* and the Jupyter Notebook [28]) are shown in the BioPortainer Workbench User Manual.

Finally, it should be mentioned that, in spite of its wide flexibility, the *BioPortainer Pipeline Runner* was designed as a tool for performing workflow analyses based on Docker. Thus, NextFlow [17] executions based on the Singularity container system [45] cannot be performed through this version of the software.

Discussion

Bioinformatics lies at the intersection of biology, computer science and statistics and often attracts professionals with limited skills for the appropriate management of computational environments. Although several initiatives have recently demonstrated the viability of using Docker to provide bioinformatics tools to researchers, most of these Docker-based systems have been developed with little concern for inexperienced users, limiting their widespread implementation in research facilities. For example, Docker image repositories, like BioShaDock [8] and Dockstore [11] provide several bioinformatics software within Docker containers, but local installation of images still requires adjustments to ensure their full operation, such as the export of network service ports and configuration of data volumes, among other procedures, which are often unclear to the final user, due to lack of proper documentation in such repositories. Moreover, absence of proper standards for image generation and lack of curatorship led to the accumulation of heterogeneous tools in these repositories. Some of these problems were addressed by the development of standardized Docker images by BioContainers [12], which allows access to more than 2000 bioinformatics tools from the Bioconda [25] repository. However, BioContainers [12] operates exclusively through command lines, hampering its use amongst users not fully familiarized with Linux commands. Although future development of BioContainers [12] may lead to its integration with the Galaxy graphical interface through Galaxy Interactive Environments (GIEs) [46], GIE deployment is not a trivial operation, since they have complex interactions with numerous services. Moreover, implementation of the Galaxy instance [4] displays large requirements for memory and disk space and additional Galaxy tools [4] have different requirements in computer memory, I/O speed, disk space, network bandwidth, density of computing cores, and parallel environment configurations, among other issues.

Thus, the development of specific tools, capable of assisting inexperienced users is of paramount importance to ensure the widespread use of Docker-based bioinformatics resources, which may greatly contribute to improve replicability and reproducibility of data analysis, given the platform-agnostic nature of Docker systems. In fact, the widespread use of Docker in different corporate business environments has been stimulated by initiatives, such as Panamax [20], Shipyard [21],

Rancher [22], and Portainer [23], which developed graphical interfaces to help in the implementation, administration and management of Docker environments by less experienced users in many different companies/organizations that deal with Information Technology (IT), particularly for working with Big Data. Until now, however, the potential of such initiatives to assist in the assimilation of Docker technology by the bioinformatics community has never been considered. Currently, both Panamax [20] and Shipyard [21] projects have been discontinued, rendering Rancher [22] and Portainer [23] as the only alternatives available for the development of a bioinformatics-dedicated Docker management platform.

Rancher [22] is a robust software for management of Docker systems, widely employed in datacenter environments and other complex computing ecosystems. It provides a platform for deployment of Docker infrastructures in an easy and controlled way, by enabling the creation of a private platform for the implementation and administration of containers, using a web interface. However, Rancher [22] installation leads to creation of a series of parallel containers in the host machine, since it employs Kubernetes as the major orchestrator of the Docker environment, consuming considerable amounts of computational resources. Portainer [23], on the other hand, requires only one container running in the host machine, reducing resource consumption, as well as the complexity inherent to its installation, maintenance and use. In addition, Portainer [23] is used by Rancher as the default administration interface for Swarm cluster environments, adding yet another layer of complexity in using Rancher [22].

Thus, Portainer [23] was chosen as the basic platform for the development of the BioPortainer Workbench [26], as it provides a more suitable platform to accommodate the needs of the bioinformatics community, composed by a significant number of inexperienced users, sometimes working in research facilities with limited computational resources. However, the scope of the BioPortainer Workbench [26] surpasses the scope of Portainer [23], since it is not only focused on providing users with an easy-to-use graphic interface to assist in implementation and administration of Docker resources. In fact, the BioPortainer Workbench [26] provides GUIs that assist users in all steps of computational analyses, including: (i) implementation of numerous bioinformatics software within Docker containers (which can be accomplished through a series of alternative platforms) (ii) management of computational resources made available to run such containers and (iii) launching of bioinformatics applications, with various degrees of complexity, through a set of unique tools (not originally present in Portainer [23], such as the *Job Runner*, *GUI Runner* and *Pipeline Runner*). Moreover, the BioPortainer Workbench [26] presents a series of unique computational resources, when compared to Portainer [23], such as the possibility of running GPU-accelerated applications (with the aid of the NVIDIA-Docker [33] plug-in) and the implementation of *Docker-in-Docker* (DinD) environments, allowing additional containerization of processes, thus improving safety and management of resources. Finally, the BioPortainer Workbench [26] also offers unique resources that help to ensure replicability and reproducibility of data analysis (a major concern in bioinformatics research), by allowing the exchange of detailed protocols (with the aid of the Jupyter Notebook [28]) and executions (encapsulated in Docker images, with the aid of the *BioPortainer Job Runner*).

Thus, the BioPortainer Workbench [26] represents a pioneering effort in developing a highly comprehensive and easy-to use Docker platform focused on bioinformatics, which may greatly assist in the dissemination of Docker virtualization technology among laboratories, contributing to improve replicability and reproducibility of results in this complex field of research.

Availability of source code and requirements

Lists the following:

- Project name: BioPortainer project
- Site: https://github.com/BioPortainer/BioPortainer
- Archive: http://doi.org/10.5281/zenodo.2377428
- RRID: SCR_017058
- Operating system(s): Platform independent
- Programming language: Go, Python
- Other requirements: Docker, Docker Compose
- License: MIT

List of abbreviations

JSON, JavaScript Object Notation; NGS, Next Generation Sequencing; PaaS, Platform-as-a-Service; RNA-seq, RNA sequencing; SaaS, Software-as-a-Service; VM, Virtual Machine; DaaS, Desktop-as-a-Service; IT, Information Technology; GUI, Graphical user interface; CLI, Command Line Interface.

Consent for publication

Not applicable.

Competing Interests

The authors declare no competing interests.

Funding

This work was supported by grants from Fundação de Amparo à Pesquisa do Estado de São Paulo (FAPESP), grants #17/13197-8 and #17/08112-3. FBM, DAB and MMN are recipients of scholarship grants from Coordenação de Aperfeiçoamento de Pessoal de Nível Superior (CAPES), while RSG is the recipient of a scholarship grant from Conselho Nacional de Desenvolvimento Científico e Tecnológico (CNPq).

Author's Contributions

FBM conceived and developed the software; DAB, RSG and MMN developed and tested the JSON files for the BioPortainer GUI Runner and assisted in testing the software under different circumstances; DLJ, RCO and LRN supervised the study and wrote the manuscript.

Additional Files

- Supplementary File 1: The BioPortainer Workbench User Manual v1.0

References

1. Google Cloud, Google Genomics; 2018. https://cloud.google.com/genomics/, accessed 19 jun 2018.
2. Amazon, Amazon Web Services: Genomics in the Cloud; 2018. https://aws.amazon.com/health/genomics/, accessed 19 jun 2018.
3. Microsoft Azure, Microsoft Azure: Cloud Computing; 2018. https://azure.microsoft.com/, accessed 19 jun 2018.
4. Blankenberg D, Coraor N, Von Kuster G, Taylor J, Nekrutenko A. Integrating diverse databases into an uni-

fied analysis framework: a Galaxy approach. Database 2011;2011:1–9.
5. Afgan E, Chapman B, Taylor J. CloudMan as a platform for tool, data, and analysis distribution. BMC Bioinformatics 2012 Nov;13(1):315.
6. AbdelBaky M, Parashar M, Kim H, Jordan KE, Sachdeva V, Sexton J, et al. Enabling High-Performance Computing as a Service. Computer 2012;45(10):72–80.
7. Docker Project, Docker; 2018. https://www.docker.com, accessed 19 jun 2018.
8. Moreews F, Sallou O, Ménager H. BioShaDock: a community driven bioinformatics shared Docker-based tools registry. F1000Research 2015;4.
9. Hosny A, Vera-Licona P, Laubenbacher R, Favre T. AlgoRun: a Docker-based packaging system for platform-agnostic implemented algorithms. Bioinformatics 2016;32(15):2396–2398.
10. Hung LH, Kristiyanto D, Lee SB, Yeung KY. Guidock: using docker containers with a common graphics user interface to address the reproducibility of research. PloS one 2016;11(4):e0152686.
11. O'Connor BD, Yuen D, Chung V, Duncan AG, Liu XK, Patricia J, et al. The Dockstore: enabling modular, community-focused sharing of Docker-based genomics tools and workflows. F1000Research 2017;6.
12. da Veiga Leprevost F, Grüning B, Alves Aflitos S, Röst H, Uszkoreit J, Barsnes H, et al. BioContainers: an open-source and community-driven framework for software standardization. Bioinformatics 2017;33(16):580–2582.
13. Galaxy Project, Galaxy Containers; 2018. https://docs.galaxyproject.org/en/master/admin/special_topics/mulled_containers.html, accessed 19 jun 2018.
14. Menegidio FB, Jabes DL, Costa de Oliveira R, Nunes LR. Dugong: a Docker image, based on Ubuntu Linux, focused on reproducibility and replicability for bioinformatics analyses. Bioinformatics 2018;34(3):514–515.
15. Köster J, Rahmann S. Snakemake—a scalable bioinformatics workflow engine. Bioinformatics 2012;28(19):2520–2522.
16. Amstutz P, Crusoe M, Tijanić N, Chapman B, Chilton J, Heuer M, et al. Common Workflow Language, v1.0 2016;.
17. Di Tommaso P, Chatzou M, Floden EW, Barja PP, Palumbo E, Notredame C. Nextflow enables reproducible computational workflows. Nature Biotechnology 2017 Apr;35(4):316–319.
18. Naik N. Applying Computational Intelligence for enhancing the dependability of multi-cloud systems using Docker Swarm. Computational Intelligence (SSCI), 2016 IEEE Symposium Series on 2016;p. 1–7.
19. Huang CH, Lee CR. Enhancing the Availability of Docker Swarm Using Checkpoint-and-Restore. Pervasive Systems, Algorithms and Networks, 2017 11th International Conference on Frontier of Computer Science and Technology, 2017 Third International Symposium of Creative Computing (ISPAN-FCST-ISCC), 2017 14th International Symposium on 2017;p. 357–362.
20. CenturyLink Labs, Panamax-UI; 2018. https://github.com/CenturyLinkLabs/panamax-ui, accessed 19 jun 2018.
21. Shipyard Project, Shipyard: Composable Docker Management; 2018. https://github.com/shipyard/shipyard, accessed 19 jun 2018.
22. Rancher Labs, Rancher Labs: Your Enterprise Kubernetes Platform; 2018. https://rancher.com/, accessed 19 jun 2018.
23. Portainer Project, Portainer: Simple management UI for Docker; 2018. https://github.com/portainer/portainer, accessed 19 jun 2018.
24. Conda Project, Conda: open source package management system and environment management system; 2017. https://conda.io/docs/, accessed 19 jun 2018.
25. Grüning B, Dale R, Sjödin A, Chapman BA, Rowe J, Tomkins-Tinch CH, et al. Bioconda: sustainable and comprehensive software distribution for the life sciences. Nature methods 2018;15(7):475.
26. Menegidio FB, BioPortainer; 2018. https://github.com/LaBiOS/BioPortainer, accessed 19 jun 2018.
27. Jenkins Project, Jenkins: build great things at any scale; 2006. https://jenkins.io, accessed 19 jun 2018.
28. Jupyter Project, Jupyter Project; 2018. http://jupyter.org, accessed 19 jun 2018.
29. JSON ORG, JSON Manual; 2018. https://www.json.org/, accessed 19 jun 2018.
30. Travis CI, Travis CI: Test and Deploy with Confidence; 2018. https://travis-ci.org/, accessed 19 jun 2018.
31. Cicle CI, Cicle CI: Continuous Integration and Delivery; 2018. https://circleci.com, accessed 19 jun 2018.
32. Play with Docker, Play-with-Docker; 2018. https://labs.play-with-docker.com, accessed 19 jun 2018.
33. NVIDIA-Docker, NVIDIA-Docker: Build and run Docker containers leveraging NVIDIA GPUs; 2018. https://github.com/NVIDIA/nvidia-docker, accessed 19 jun 2018.
34. NVIDIA CUDA Toolkit, NVIDIA CUDA Toolkit: Develop, Optimize and Deploy GPU-accelerated Apps; 2018. https://developer.nvidia.com/cuda-toolkit, accessed 19 jun 2018.
35. NVIDIA cuDNN, NVIDIA cuDNN: GPU Accelerated Deep Learning; 2018. https://developer.nvidia.com/cuda-toolkit, accessed 19 jun 2018.
36. PIP Project, PIP - Python Install Package; 2018. https://pypi.org/project/pip/, accessed 19 jun 2018.
37. Jackman S, Birol I. Linuxbrew and Homebrew for cross-platform package management. F1000Research 2016;5:1795.
38. Gentleman RC, Carey VJ, Bates DM, Bolstad B, Dettling M, Dudoit S, et al. Bioconductor: open software development for computational biology and bioinformatics. Genome Biology (Online Edition) 2004;5:R80.
39. Afgan E, Baker D, van den Beek M, Blankenberg D, Bouvier D, Čech M, et al. The Galaxy platform for accessible, reproducible and collaborative biomedical analyses. Nucleic acids research 2016;44(w1):w3–w10.
40. Afgan E, Baker D, Batut B, vandenBeek M, Bouvier D, Čech M, et al. The Galaxy platform for accessible, reproducible and collaborative biomedical analyses: 2018 update. Nucleic Acids Research 2018;p. gky379.
41. Galaxy Project, Planemo: Command-line utilities to assist in developing tools for the Galaxy Project; 2018. https://github.com/galaxyproject/planemo, accessed 19 jun 2018.
42. Dawn F, Tiwari B, Booth T, Houten S, Swan D, Bertrand N, et al. Open Software for Biologists: from famine to feast. Nature biotechnology 2006 08;24:801–3.
43. R Development Core Team, R: A Language and Environment for Statistical Computing; 2012. http://www.R-project.org, accessed 19 jun 2018.
44. RStudio Team, RStudio: Integrated Development Environment for R. Boston, MA; 2015. http://www.rstudio.com/, accessed 19 jun 2018.
45. Kurtzer GM, Sochat V, Bauer MW. Singularity: Scientific containers for mobility of compute. PLOS ONE 2017 05;12(5):1–20.
46. Grüning BA, Rasche E, Rebolledo-Jaramillo B, Eberhard C, Houwaart T, Chilton J, et al. Jupyter and Galaxy: Easing entry barriers into complex data analyses for biomedical researchers. PLOS Computational Biology 2017 05;13:1–10.

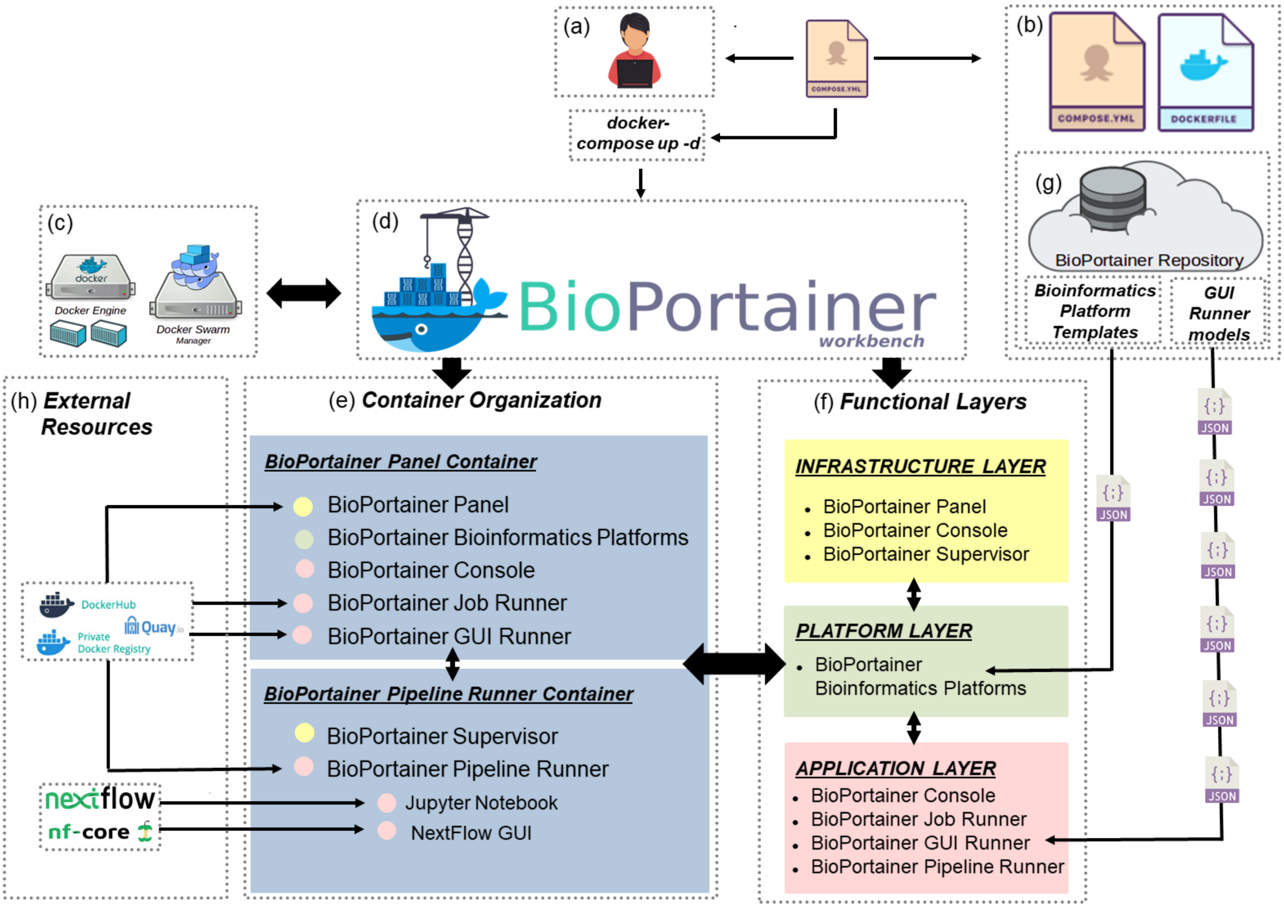

Figure 1. Overview of the BioPortainer Workbench Architecture: Users (a) can access the BioPortainer Workbench image (b) from the projects webpage and install the software either in single Docker engines, or in Swarm clusters (c). Once Installed, the BioPortainer Workbench (d) consists of two containers (e): the BioPortainer Panel and the BioPortainer Pipeline Runner. These two containers provide access to a series of tools that operate in three distinct functional layers (f), allowing users to implement bioinformatics-related tools (using a variety of alternative platforms), manage resources from the Docker environment and launch their analyses using both CLI-based and GUI-based interfaces (preconfigured through a series of JSON files, available from the BioPortainer Workbench repository [g]), as well as commands/scripts/pipelines harnessed from external repositories (h). See text for details.

Click here to access/download
Supplementary Material
BioPortainer Workbench User Manual New.pdf

Personal Cover

Click here to access/download;Personal Cover;Letter Bioportainer- GigaScience - Luiz - Revision 3.docx

São Bernardo do Campo, March 19th, 2019

Dr. Scott Edmunds
Executive Editor
GigaScience

Dear Dr. Edmunds.

In response to your communication dated March, 19th, 2019, we have edited the last revision of manuscript GIGA-D-18-00229, entitled "**BioPortainer Workbench: a versatile and user-friendly system that integrates implementation, management and use of Bioinformatics resources in Docker environments**".

According to your instructions, we edited the Correspondence Address, including a telephone number to contact the corresponding authors, as well as their respective ORCID IDs (see page 1 of the attached pdf file, containing the manuscript). We also added the RRID number of the BioPortainer Workbench project to the Methods section (see section entitled *BioPortainer Workbench Architecture*, on Page 2) and included a complete link to the Zenodo DOI in the section *Availability of Source Code and Requirements*, on page 7).

Finally, the Zenodo landing page has been updated and now contains a thorough description of the BioPortainer Workbench project. I confirm that this page contains the same files as the ones available from GitHub.

Please, let me know if any other changes are necessary.

Sincerely.

Luiz R. Nunes, Ph.D.
Centro de Ciências Naturais e Humanas – Universidade Federal do ABC (UFABC)
Alameda da Universidade, s/n, São Bernardo do Campo, SP - CEP: 09606-045, Brazil.
Phone/Fax: +55 (11) 4996-8371/4996-3166.
e-mail: Luiz.Nunes@ufabc.edu.br

Response Reviewers

Click here to access/download;Personal Cover;Response to reviewers - GigaScience - Revision 2.docx

Response to Reviewers

Reviewer reports:

Reviewer #1: The authors have addressed all the comments by the reviewer satisfactorily, and the manuscript is recommended for acceptance publication.

Answer: Dear Reviewer. Thank you for your comments and suggestions, during the first round of peer review, which helped us to significantly improve the manuscript.

Reviewer #2: By trying out the new version of BioPortainer (now called BioPortainer Workbench),it is achieve these goals that easy to use Docker platform focused on bioinformatics, and allowing running program in any type of computing ecosystem,that help to ensure replicability and reproducibility of data analysis to researchers.Moreover, the BioPortainer Workbench also presents a series of unique computational resources,such as the possibility of running GPU-accelerated applications, the function of Docker-in-Docker environments and allowing the exchange of detailed protocols and executions with the aid of the Juyter Notebook, or unique BioPortainer Job Runner tool.

It's better to be consistent installation method of instructions document of https://hub.docker.com/r/bioportainer/bioportainer-pipeline-runner, https://github.com/BioPortainer/BioPortaine and BioPortainer Workbench User Manual。

Answer: Dear Reviewer. We standardized the instructions for software installation described both in the main manuscript, as well as in the current version of the BioPortainer Workbench User Manual (which accompanies the manuscript as supplementary material).

Installation instructions contained at the following URLs have also been corrected and formatted to the same standard:

https://hub.docker.com/r/bioportainer/bioportainer-pipeline-runner

https://github.com/BioPortainer/BioPortainer

https://bioportainer.github.io/BioPortainer

We appreciate your careful and thorough revision.
