# Supplementary material for: Bioportainer Workbench: a versatile and user-friendly system that integrates implementation, management, and use of bioinformatics resources in Docker environments
Source: Gigascience. 2019 Apr 25;8(4):giz041. doi: 10.1093/gigascience/giz041 (PMC6482343; doi:10.1093/gigascience/giz041)
Supplement: Supplemental File [file giz041_supplemental_file.pdf]

BIOPORTAINER WORKBENCH USER MANUAL v1.0

Index

Page

Preface ——— 2

Part 1 – Deploying and configuring the BioPortainer Workbench (BPWB) — 3

Scenario 1 – Installing BPWB in a Docker Engine in a local computer ——— 3

1.1 – Deploying BPWB through the Docker Compose ——— 3
1.2 - Configuring BPWB ——— 4
1.3 - Deploying BPWB manually ——— 4

Scenario 2 – Emulating the installation of BPWB in a Docker Engine with the aid of Play-with-Docker ——— 11

Part 2 – Deploying Containers with the BPWB Platforms ——— 13

Scenario 1 – Deploying the Dugong Clean CMD ——— 13

Scenario 2 – Deploying Galaxy Stable ——— 18

Part 3 – Launching Bioinformatics applications and analyses with alternative BPWB tools ——— 20

Scenario 1 – Launching Bioinformatics applications with the BioPortainer Console ——— 20

Scenario 2 – Launching Bioinformatics applications with the BioPortainer Job Runner ——— 23

Scenario 3 – Launching Bioinformatics applications with the BioPortainer GUI Runner ——— 26

Scenario 4 – Launching Bioinformatics applications with the BioPortainer Pipeline Runner ——— 29

4.1 – Running NextFlow Pipelines with the aid of the BioPortainer Pipeline Runner GUI ——— 29
4.2 – Running NextFlow Pipelines with the aid of the Jupyter Notebook ——— 31
4.3 – Analyzing Proprietary data with NextFlow Pipelines ——— 33

Table S1 - Platforms and Basic tools available through the BioPortainer Workbench platform options ——— 38

Table S2 - Tools available for launching bioinformatics applications with the aid of the BioPortainer GUI Runner ——— 42

PREFACE

The BioPortainer Workbench (BPWB) is an open-source software, developed under the MIT license and designed to assist users to interact with a comprehensive computational environment, dedicated to Bioinformatics analyses. BPWB is developed in Docker, meaning that it is platform-agnostic and can be consistently deployed and used in a wide variety of computational ecosystem, regardless the specificities of their hardware and/or operating system, which helps to ensure replicability and reproducibility of data analyses across different research facilities.

The modular structure of BPWB allows users to interact with the Docker environment at three main levels: in the *Infrastructure Layer*, the BioPortainer Workbench provides a GUI, based on the Portainer project, which allows rapid and simple implementation/management of a full Docker ecosystem; in the *Platform Layer*, the software provides a wide range of intuitive template forms that assist users in the installation and configuration of containers, carrying specific bioinformatics tools, from a variety of alternative platforms (based on CLIs, GUIs, or on a virtual desktop); finally, in the *Application Layer*, the BioPortainer Workbench provides a series of CLI-based and GUI-based interfaces to assist users in launching jobs with varying degrees of complexity (from single application analyses to complex pipelines and workflows).

This manual provides detailed instructions and demonstrations regarding installation of the software, as well as user interaction at these three functional layers. Additional information about BPWB can also be found at the BPWB Project webpage (https://bioportainer.github.io/BioPortainer).

PART 1 – DEPLOYING AND CONFIGURING THE BIOPORTAINER WORKBENCH (BPWB)

The following sections of this manual will describe all steps necessary for installing and configuring BPWB in a local host, along with the results obtained from an emulated installation, performed with the aid of the learning platform Play-with-Docker.

Scenario 1: Installing BPWB in a Docker Engine in a local computer

In the first scenario, we use a standard Docker Engine installation in an I7 notebook, containing 8GB of memory and the Ubuntu Linux operating system 1(v8.04). The installations of Docker and Docker Compose were done according to the official documentation of the Docker project. Details regarding the entire execution process are provided in the next section, along with a corresponding video file, available here. The exact time at which each action is displayed in the video is also provided (in parentheses) next to the instructions below.

1.1: Deploying BPWB through the Docker Compose:

BPWB is built to run on Docker and is very simple to deploy. Deployment scenarios can be executed on any platform, unless specified otherwise.

After installing Docker and Docker Compose, the following steps must be performed for the default **BPWB** deployment:

Test if Docker is installed and running, by typing *[00:01]*:

$ docker info

Check if there are Docker containers being executed, by typing *[00:11]*:

$ docker ps

Download **BioPortainer Workbench** compose from the repository, by typing *[00:18]*:

$ wget https://goo.gl/bNecPA -O docker-compose.yml

Create **BioPortainer Workbench** containers, by typing *[00:35]*:

$ docker-compose up -d

Confirm creation of the **BioPortainer Workbench** containers, by typing *[00:45]*:

$ docker ps

After deployment, users can use the **BioPortainer Panel** and the **BioPortainer Pipeline Runner** by accessing their service ports (9000, in the case of the **BioPortainer Panel** and 5000, 8888, and 7000, in the case of the **BioPortainer Pipeline Runner**) on the server where the **BioPortainer Workbench** is running. *[00:55]*

1.2: Configuring BPWB:

After deploying the service, some settings are required for full operation of the **BioPortainer Panel**:

- Access the address http://localhost:9000 (if the installation is local) or the IP/Host address, in case of remote installation. *[01:28]*

- Choose an administrator username and password (Figure S1). *[01:31]*

- Define the type of **Endpoint** that will be administered by the **BioPortainer Panel** (**LOCAL** in our example) (Figure S2). *[01:40]*

- Reset permissioning to default volumes (**$USERNAME_bioportainer_data** and **$USERNAME_bioportainer_workdir**) via the **VOLUMES** option in the main menu. Select **Change ownership** on each volume and change from **Administrators** to **Publics** (Figure S3). *[03:09]*

Additional optional settings can be made in the BioPortainer Panel, such as: (1) creating new users, (2) creating user teams, (3) setting new Endpoints, (4) creating groups and tags for Endpoints and (5) configurations of new Registries, among others.

Once BPWB is installed, users can have access to several aspects of the Docker environment, which can be viewed and controlled by the program´s main *Dashboard* (Figure S4), as well as to all containers present in the system (see the *Containers* option, in Figure S5).

1.3: Deploying BPWB manually

BPWB can also be implemented manually. For this, each container must be started manually by the user. This scenario is not recommended for users not fully familiarized with the Docker environment, who should prefer installation via Docker Compose, as described above.

After installing Docker, the following steps must be performed for the default **BioPortainer Workbench** deploy:

Test if Docker is installed and running, by typing:

$ docker info

Check if there are Docker containers being executed, by typing:

$ docker ps

Download **BioPortainer Panel** and **BioPortainer Pipeline Runner** images from the Docker Hub, by typing:

```
$ docker pull bioportainer/bioportainer-panel
$ docker pull bioportainer/bioportainer-pipeline-runner
```

Confirm download the BioPortainer images, by typing:

```
$ docker images
```

Create **BioPortainer Workbench** containers, by typing:

```
$ docker run -d \
	--name BioPortainer-Panel \
	--restart always \
	-p 9000:9000 \
	-v /var/run/docker.sock:/var/run/docker.sock \
	-v ${PWD}/bioportainer_data:/data \
	bioportainer/bioportainer-panel

$ docker run -di \
	--name BioPortainer-Pipeline-Runner \
	--privileged \
	--restart always \
	-p 5000:5000 -p 8888:8888 -p 7000:7000 \
	-v ${PWD}/bioportainer_workdir:/root/workdir/data \
	bioportainer/bioportainer-pipeline-runner
```

Confirm creation of the **BioPortainer Workbench** containers, by typing:

```
$ docker ps
```

After deployment, users can use the **BioPortainer Panel** and the **BioPortainer Pipeline Runner** by accessing their service ports (9000, in the case of the **BioPortainer Panel** and 5000, 8888, and 7000, in the case of the **BioPortainer Pipeline Runner**) in the server where the **BioPortainer Workbench** is running. *[00:55]*

NOTE: Once implemented, the steps in **section 1.2** must be followed to complete the **BioPortainer Panel** configuration.

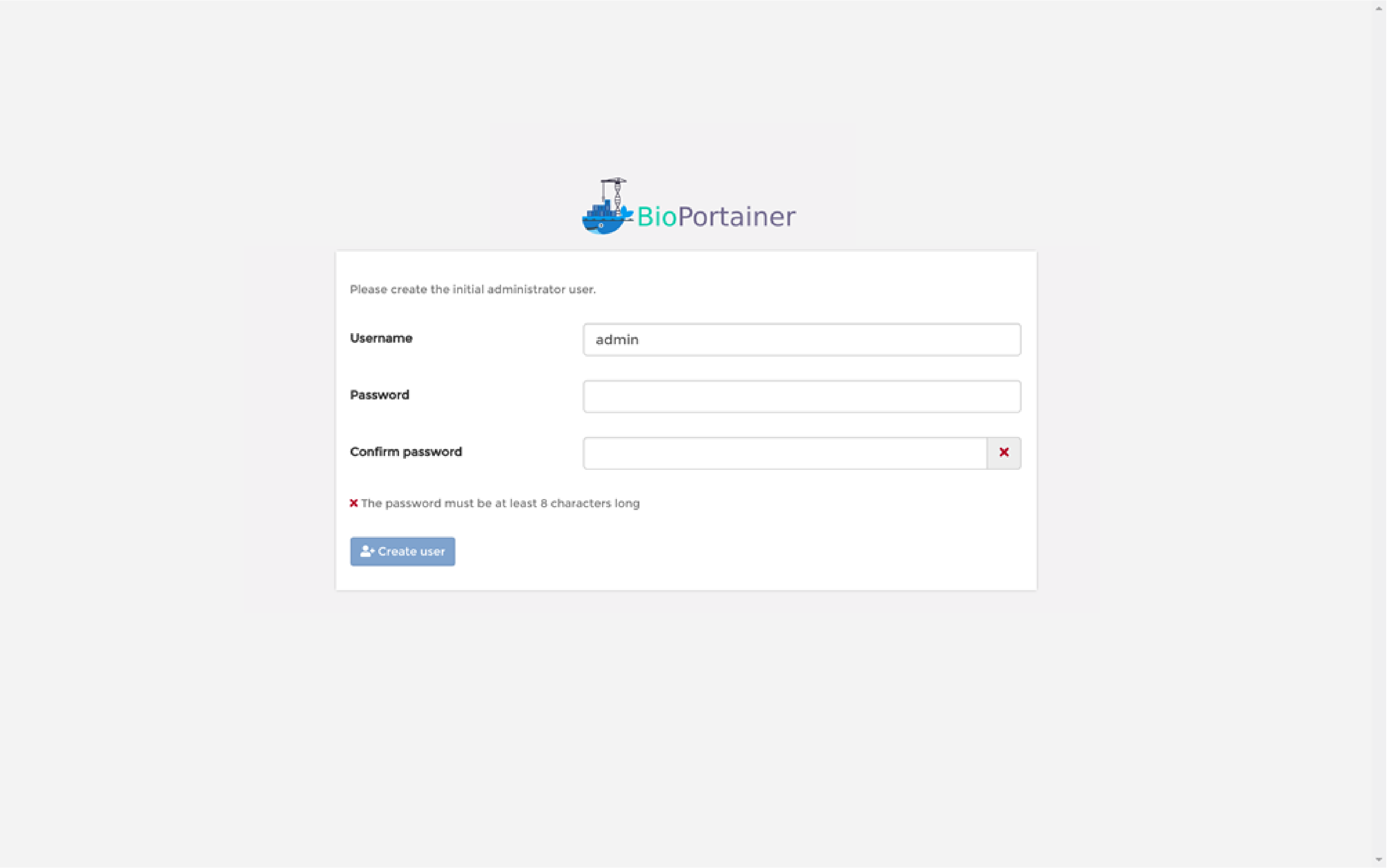

Figure S1. Administrative user configuration screen, which allows setting of username and password for administration of the BioPortainer Panel.

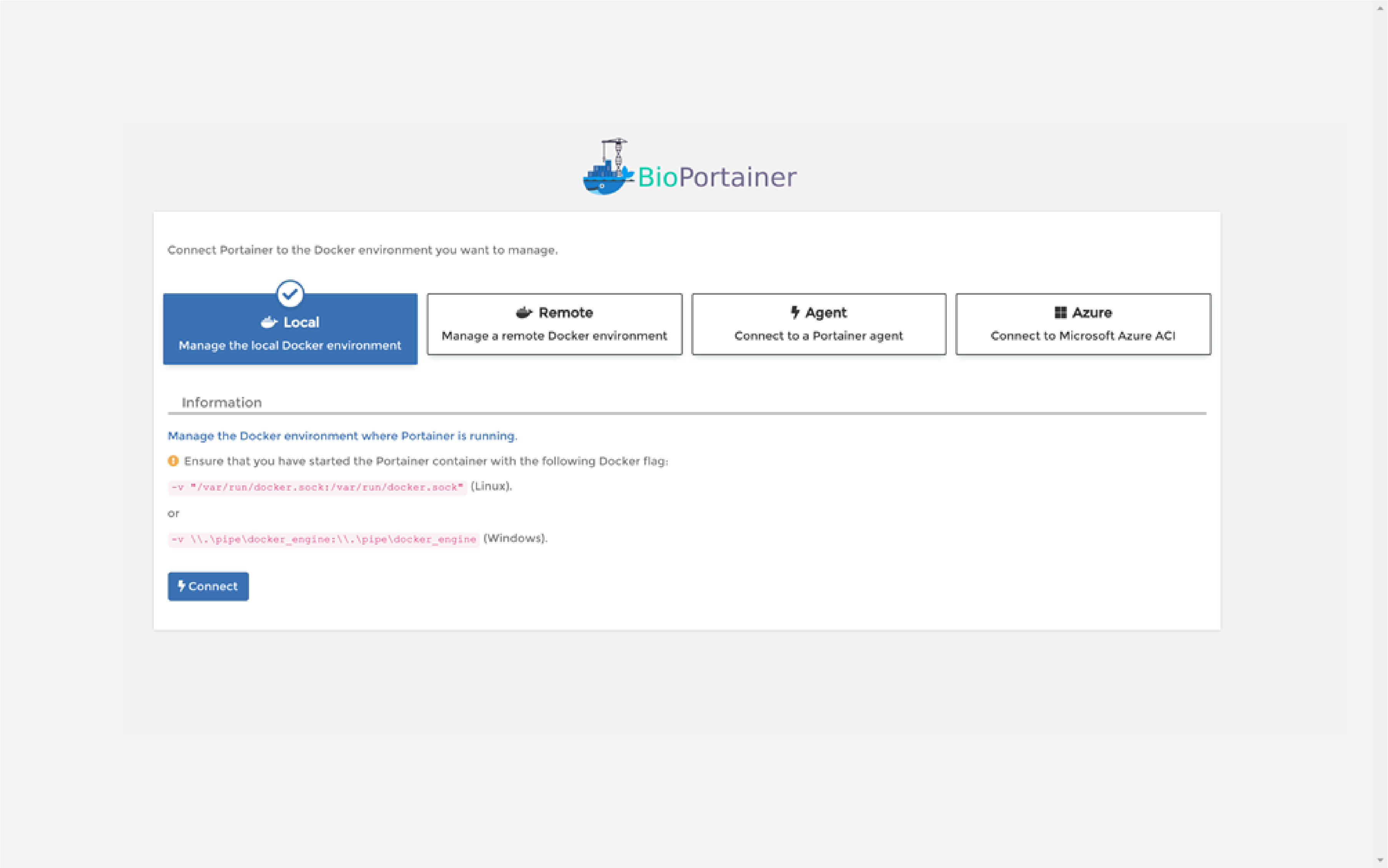

Figure S2. Setting up the Docker Environment. Selection of the Endpoint that will be administered by the BioPortainer Workbench. In the example presented above, the Endpoint will be local.

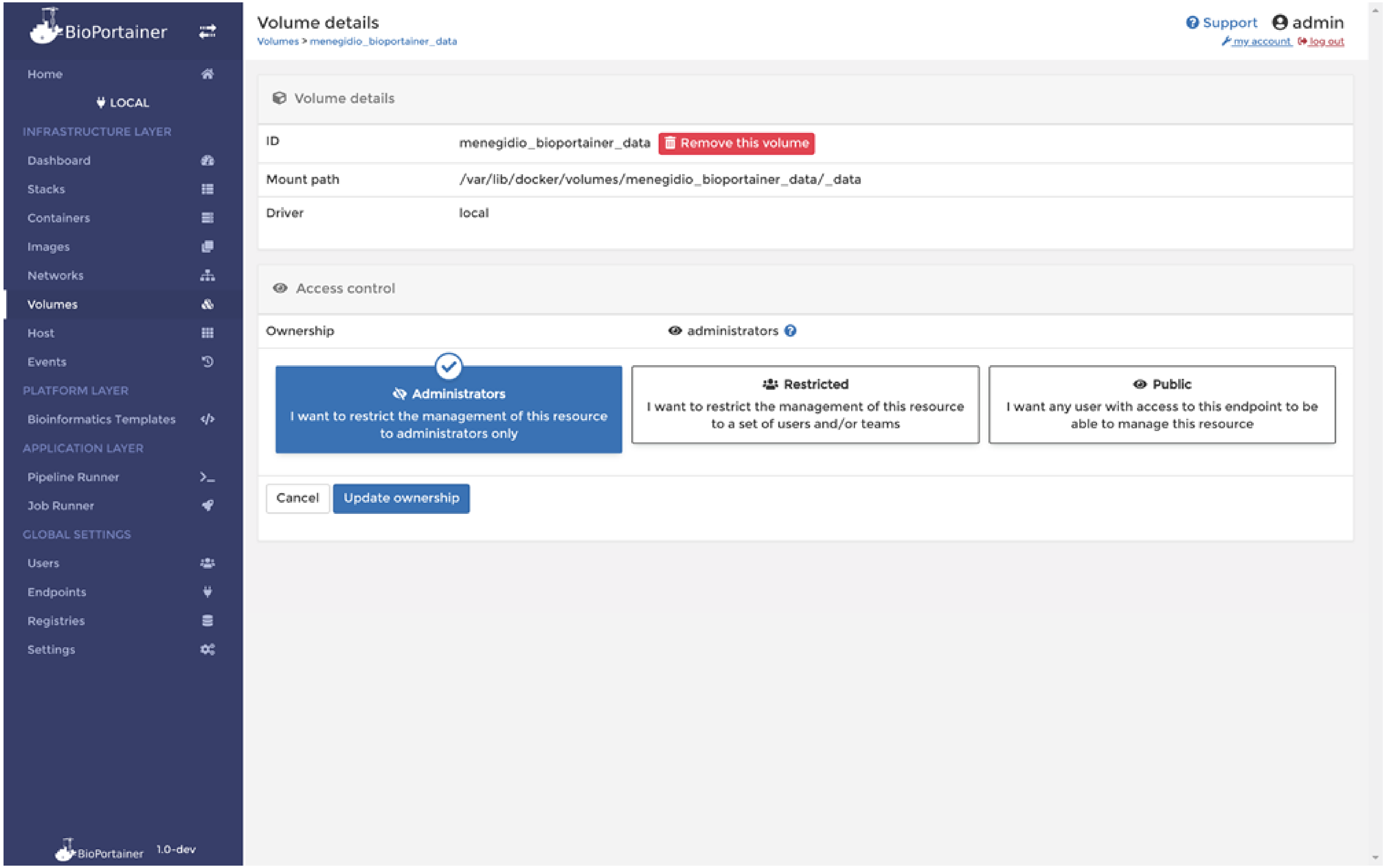

Figure S3. Volume permission setting. BioPortainer Workbench volumes (bioportainer_data and bioportainer_workdir) are configured for manipulation by the administrative user only. The configuration for public manipulation of volumes is necessary so that all users can manipulate the files generated by the BioPortainer Pipeline Runner.

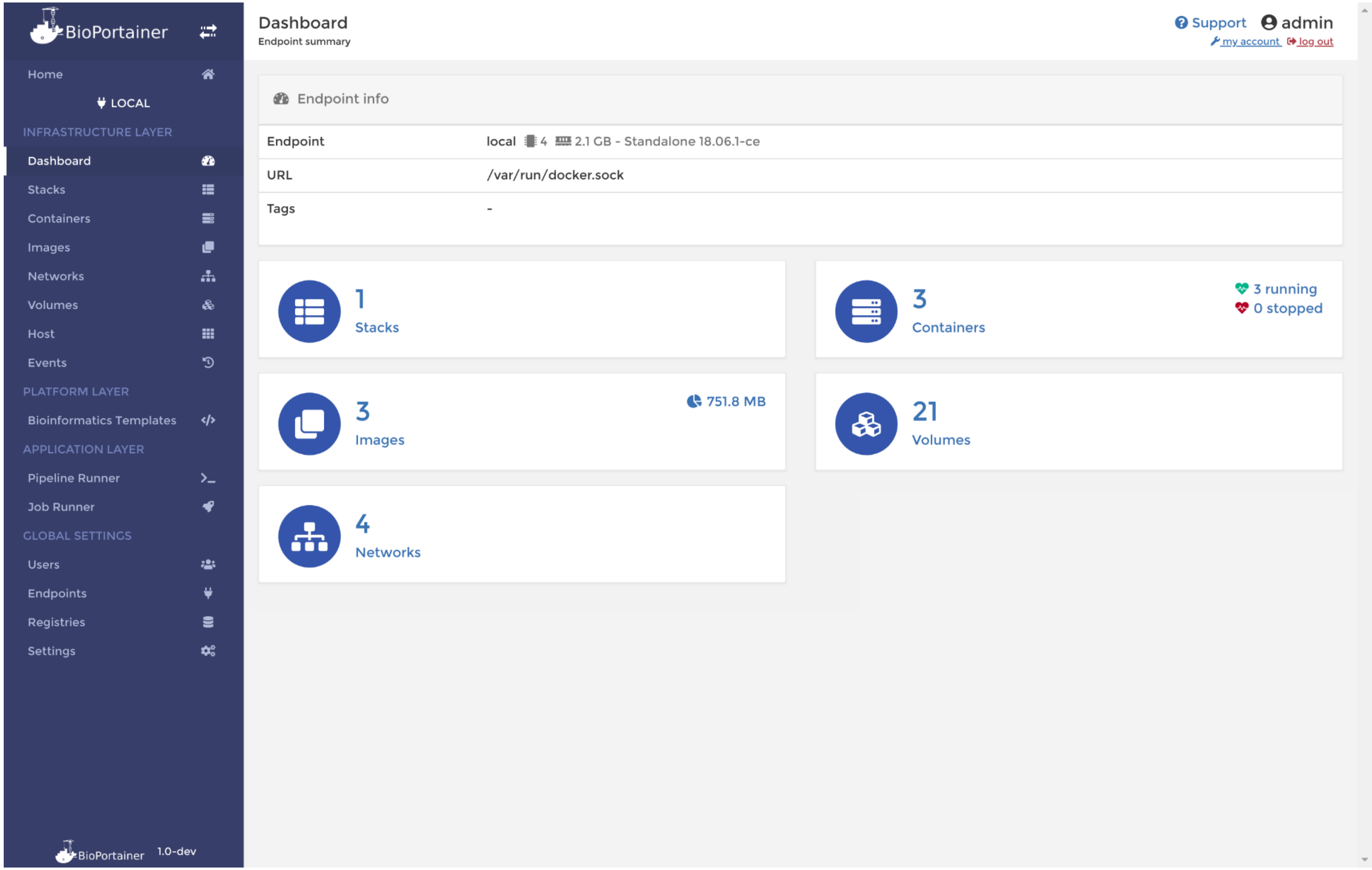

Figure S4 – Option *Dashboard*, from the BioPortainer Panel main menu, provides users with general information about the managed host, including: (i) stacks (the collection of services that make up an application in a specific environment); (ii) number of containers created (running or stopped); (iii) number of volumes and networks created; (iv) number of images available on the host (and space consumed by them); (v) hostname of the machine and (vi) details regarding the amount of CPU and memory available in the host machine.

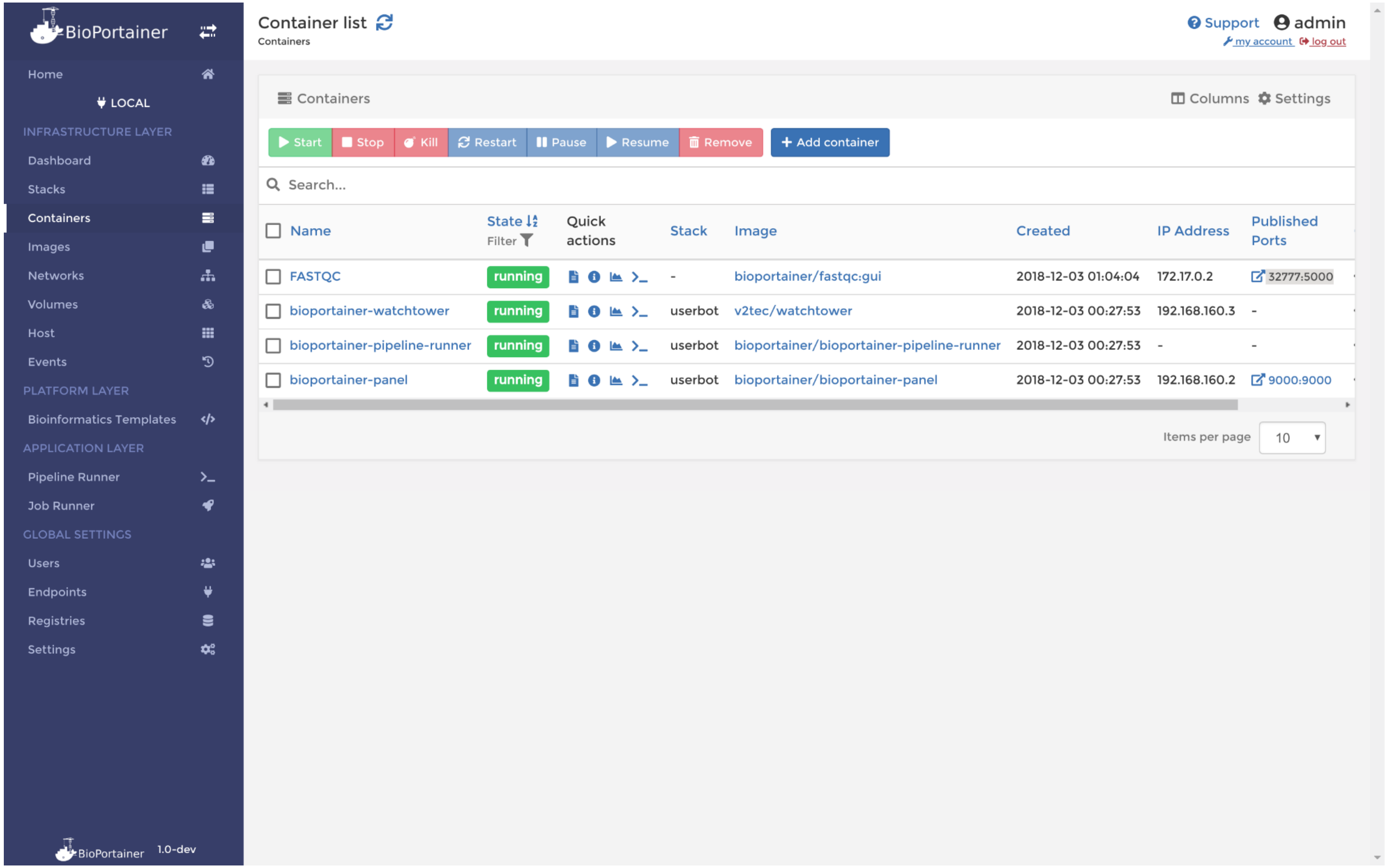

Figure S5 - Option *Containers*, from the BioPortainer Panel main menu, allows direct and simple administration of the containers installed in the host machine. Options for start, stop, console launching, restart, kill, among others, can be accessed, as well as the published ports for each container deployed in the Docker environment.

Scenario 2: Emulating the installation of BPWB in a Docker Engine with the aid of Play-with-Docker

In this scenario, we use the learning platform Play-with-Docker to configure a virtual machine containing the Docker Engine installed. Next, use the **ADD NEW INSTANCE** option to create the virtual machine test *[00:06]* and the following commands to deploy **BPWB** (Figure S6). Details regarding the entire execution process are provided in the next section, along with a corresponding video file, available here. The exact time at which each action is displayed in the video is also provided (in parentheses) next to the instructions below.

After deploying the Docker Instance, the following steps must be performed for the default installation of **BPWB**:

Check if there are Docker containers being executed, by typing *[00:16]*:

$ docker ps

Download **BioPortainer Workbench** compose from the repository, by typing *[00:23]*:

$ wget
https://raw.githubusercontent.com/BioPortainer/Repository/master/Compose/docker-compose.yml

Create **BioPortainer Workbench** containers, by typing *[00:50]*:

$ docker-compose up -d

Confirm creation of the **BioPortainer Workbench** containers, by typing *[01:10]*:

$ docker ps

NOTE: After installation of the **BioPortainer Workbench** containers, the ports of the started services will be automatically available in the Play-with-Docker interface, facilitating access to each tool (Figure S6). Click on each of the ports to access the services. *[01:14]*

After deployment, users can use the **BioPortainer Panel** and the **BioPortainer Pipeline Runner** by accessing their service ports (9000, in the case of the **BioPortainer Panel** and 5000, 8888, and 7000, in the case of the **BioPortainer Pipeline Runner**) in the server where the **BioPortainer Workbench** is running. *[01:24]*

NOTE: Once implemented, the steps in **section 1.2** must be followed to complete the **BioPortainer Panel** configuration.

WARNING: We used **Play-with-Docker** to make **BioPortainer** test labs quickly and easily available BPWB users, without the need for dedicated infrastructure. However, while such tests have proven to be fully functional on the dates in which our videos were recorded, we would like to remind users that maintenance of the **Play-with-Docker Project** is the sole responsibility of its developers. Thus, alterations made in the platform may affect the final outcome of such simulations, using the conditions described herein.

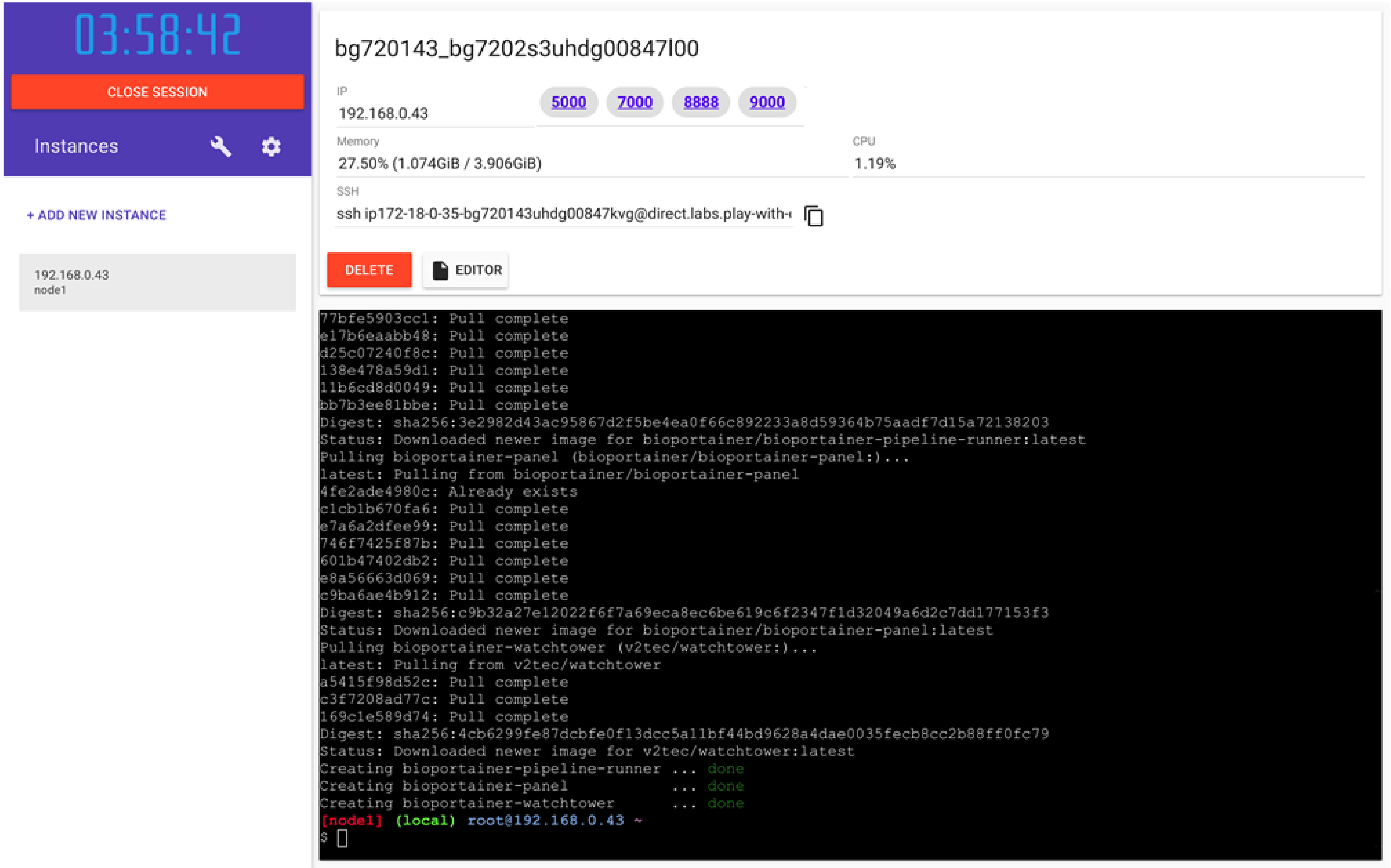

Figure S6. Play-with-Docker screen containing a virtual instance with a Docker Engine installed. After installation of the BioPortainer Workbench containers and service ports will be automatically available in the Play-with-Docker interface, providing easier access to each tool. By clicking on each of the ports, the users will be directed to the web interface of each of the services.

PART 2 – DEPLOYING CONTAINERS WITH THE BPWB PLATFORMS

Once installed, BPWB can be used to deploy containers to run a great variety of bioinformatics tools. This document provides detailed instructions for container deployment using two examples: (i) a basic installation of DugongClean CMD, a comprehensive DaaS system that allows users to install and deploy more than 3000 bioinformatics tools (Menegidio et al., 2018), and (ii) a basic installation of Galaxy Stable.

Scenario 1: Deploying Dugong Clean CMD

In this scenario, we implemented the DugongClean CMD version through the **BioPortainer Panel** using the template available from the **Bioinformatics Templates** menu. Details regarding the entire execution process are provided in the next section, along with a corresponding video file, available here. The exact time at which each action is displayed in the video is also provided (in parentheses) next to the instructions below.

After deploying and configuring **BPWB**, the following steps must be performed, through the **BioPortainer Panel** interface, to achieve a default installation of **Dugong Clean CMD**:

1) Visit the **Bioinformatics Templates** option in the **BioPortainer Panel** main menu to see the available templates (Figure S7). *[00:01]*

2) Select the **Show container templates** option.

3) Select **Dugong Platform.** *[00:07]*

4) Select **DugongClean CMD** to configure the platform installation form (Figure S8). *[00:10]*

5) Disable the **Activate Access Control** option. *[00:14]*

6) Enter a name for the container (for example: dugongclean-cmd). *[00:16]*

7) Select **Show Advanced Options.** *[00:21]*

8) Map container volume to a folder created on the host. *[00:23]*

9) Create the container by clicking the **Deploy Container** button. [*00:41*]

After proceeding with image download and container deployment, user can fully administer the dugongclean-cmd container through the **BioPortainer Panel** interface, performing functions such as:

1) View and manage container settings by implementing the **Containers** option from the main menu. *[00:45]*

2) Access the mapped service ports between the container and the host. An example is the DugongClean CMD Web Terminal port. *[00:52]*

3) Access the container configuration information, such as the mapping of volumes between the installed container and the host. *[01:32]*

4) Selecting the Console icon (**> _**) and clicking on Connect, user will gain access to the command bash of the installed container, being able to execute commands directly in the container (Figure S9). *[01:43]*

NOTE: Additional functions are presented in the **BioPortainer Workbench** presentation videos, available **here** and **here**.

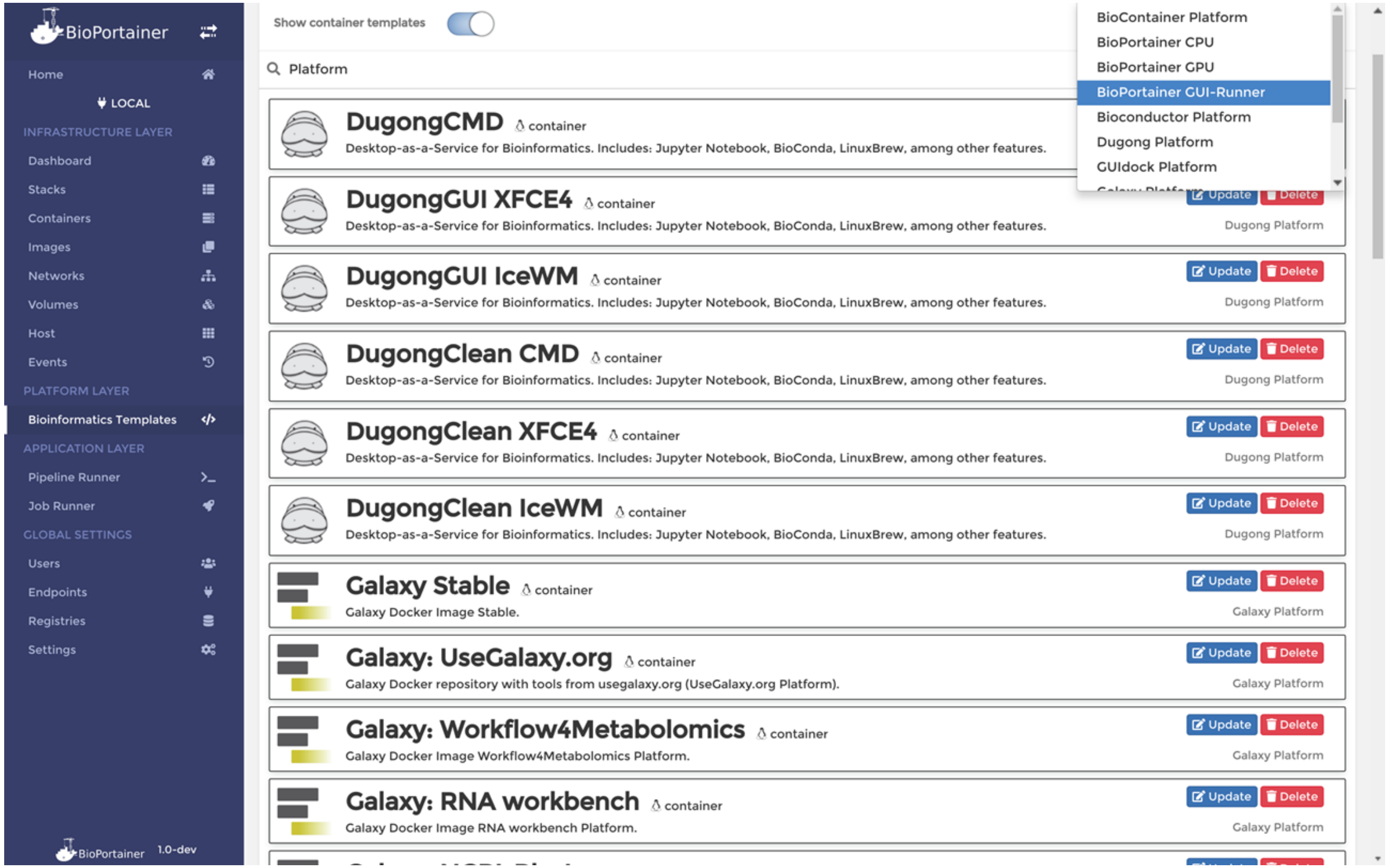

Figure S7 - Option *Bioinformatics Templates*, from the BioPortainer Panel main menu, provides access to the preconfigured templates for all Bioinformatics Platforms of the BioPortainer Workbench. Each platform can be selected by a drop-down menu, at the upper right corner of the figure and the basic tools available from each platform are shown at the center of the panel. It is also possible to add new templates and/or update/modify existing templates with the help of the *add template* and *update template* buttons (located at the top part of the panel and next to each tool, respectively). Details on all available platforms and their basic tools can be found in Supplementary Table 1.

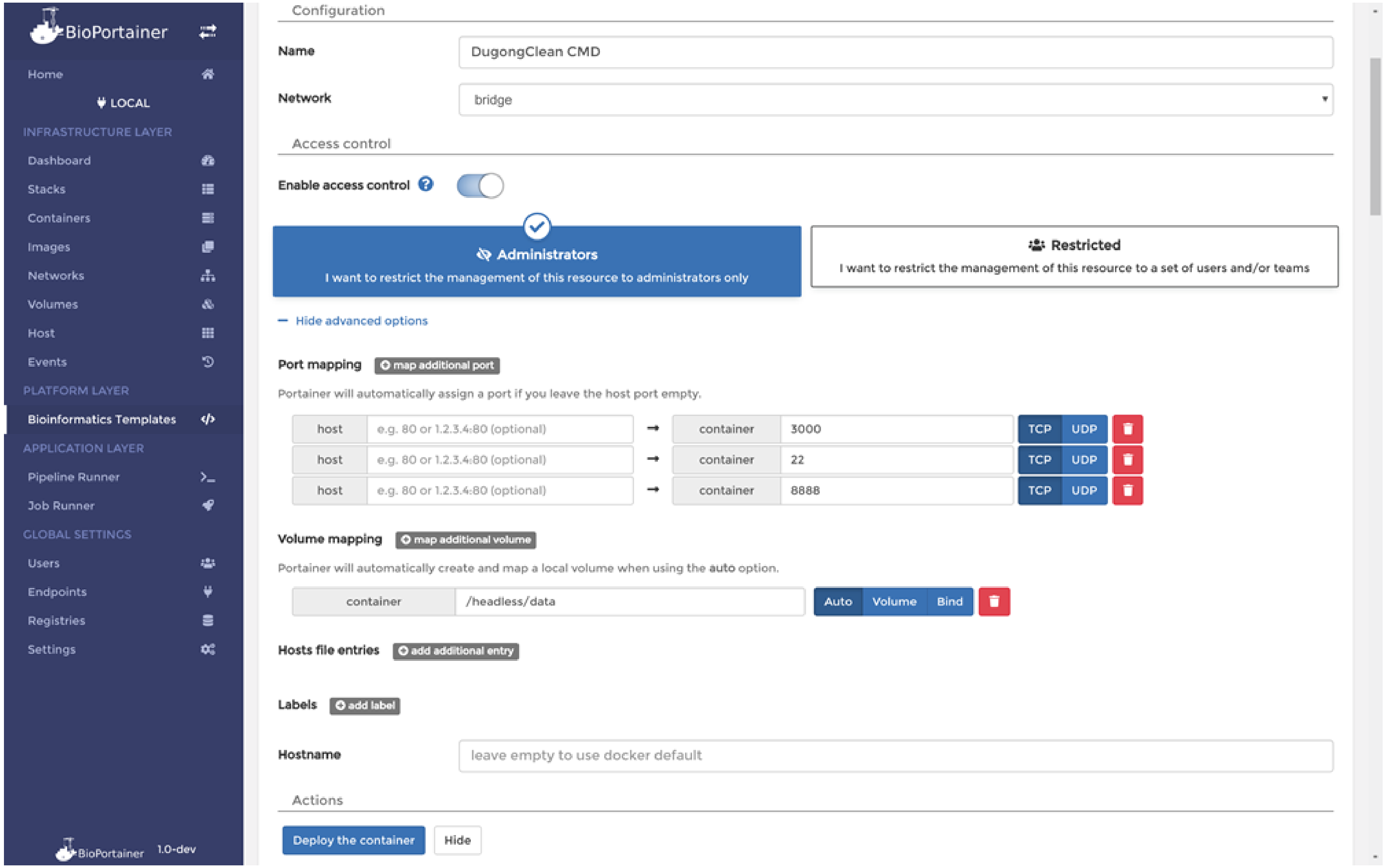

Figure S8. Options for installing and configuring the Dugong Clean CMD application from the BioPortainer Workbench repository. Through a simple and intuitive form, users can deploy a local or remote instance of DugongClean CMD from the Bioinformatics Templates menu and then use it to implement thousands of bioinformatics tools from BioConda, BioLinux and LinuxBrew repositories. A total of 6 alternative templates are provided for different versions of Dugong.

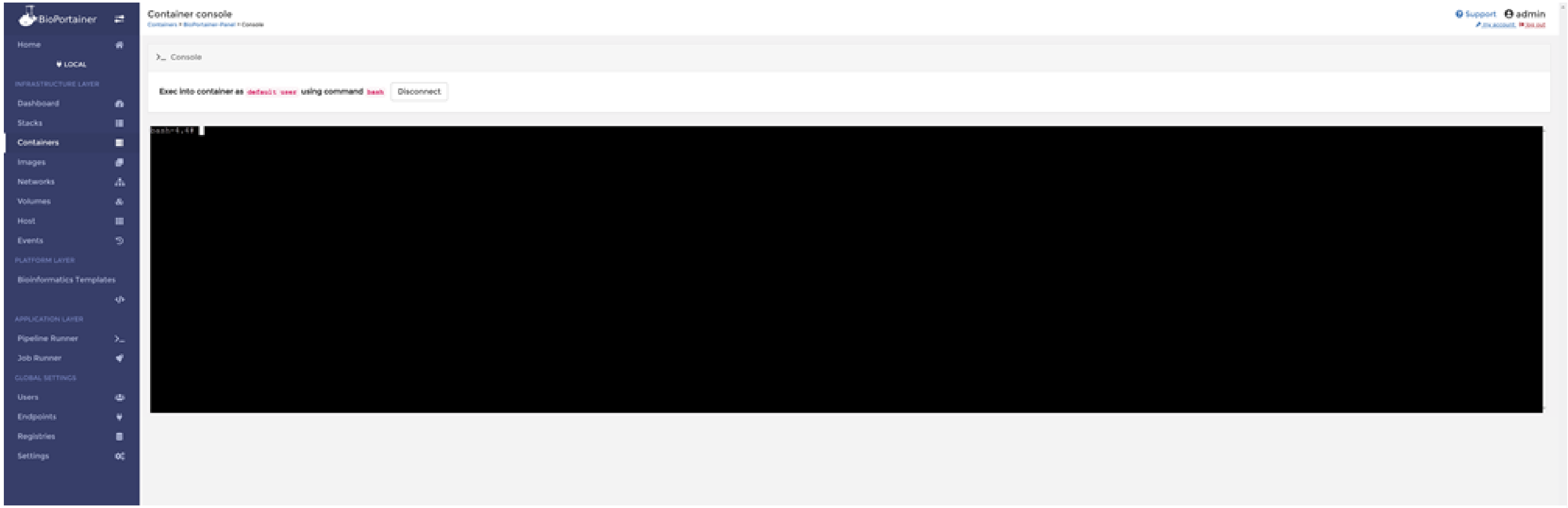

Figure S9. The BioPortainer Console. The BioPortainer Panel allows full administration of all installed containers through an interactive console, available in its interface through the **>_** icon. The only requirement for its operation is the existence of a shell installed in the Docker image, such as bash.

Scenario 2: Deploying Galaxy Stable

In this scenario, we implemented the Galaxy Stable version through the **BioPortainer Panel** using the template available in the **Bioinformatics Templates** menu. Details regarding the entire execution process are provided in the next section, along with a corresponding video file, available here. The exact time at which each action is displayed in the video is also provided (in parentheses) next to the instructions below.

The Galaxy Stable interface is one of the most widely employed bioinformatics tools available and can be intuitively installed through a pre-configured BioPortainer template, which defines forms for the main Galaxy Magic Environmental Variables available, allowing users to deploy their own personalized versions of Galaxy tools.

After deploying and configuring **BPWB**, the following steps must be performed, through the **BioPortainer Panel** interface, to achieve a default installation of **Galaxy Stable**:

1) Visit the **Bioinformatics Templates** option in the **BioPortainer Panel** main menu to see the available templates (Figure S7). *[00:01]*

2) Select the **Show container templates** option.

3) Select **Galaxy Platform.** *[00:05]*

4) Select **Galaxy Stable** to configure the platform installation form (Figure S10). *[00:06]*

5) Enter a name for the container (for example: galaxy-stable). *[00:10]*

5) Disable the **Activate Access Control** option. *[00:14]*

7) Fill in the fields for **Magic Environment Variables** (optional).

8) Create the container by clicking the **Deploy Container** button. *[00:17]*

Upon container creation, users can access the Galaxy interface through any available web browser *[02:00]*. The Galaxy login (admin) and password (admin) must be provided at this point, to grant users with administrative status, necessary for further container configuration. The entire administration of Galaxy containers can be performed through the **BioPortainer Panel** interface.

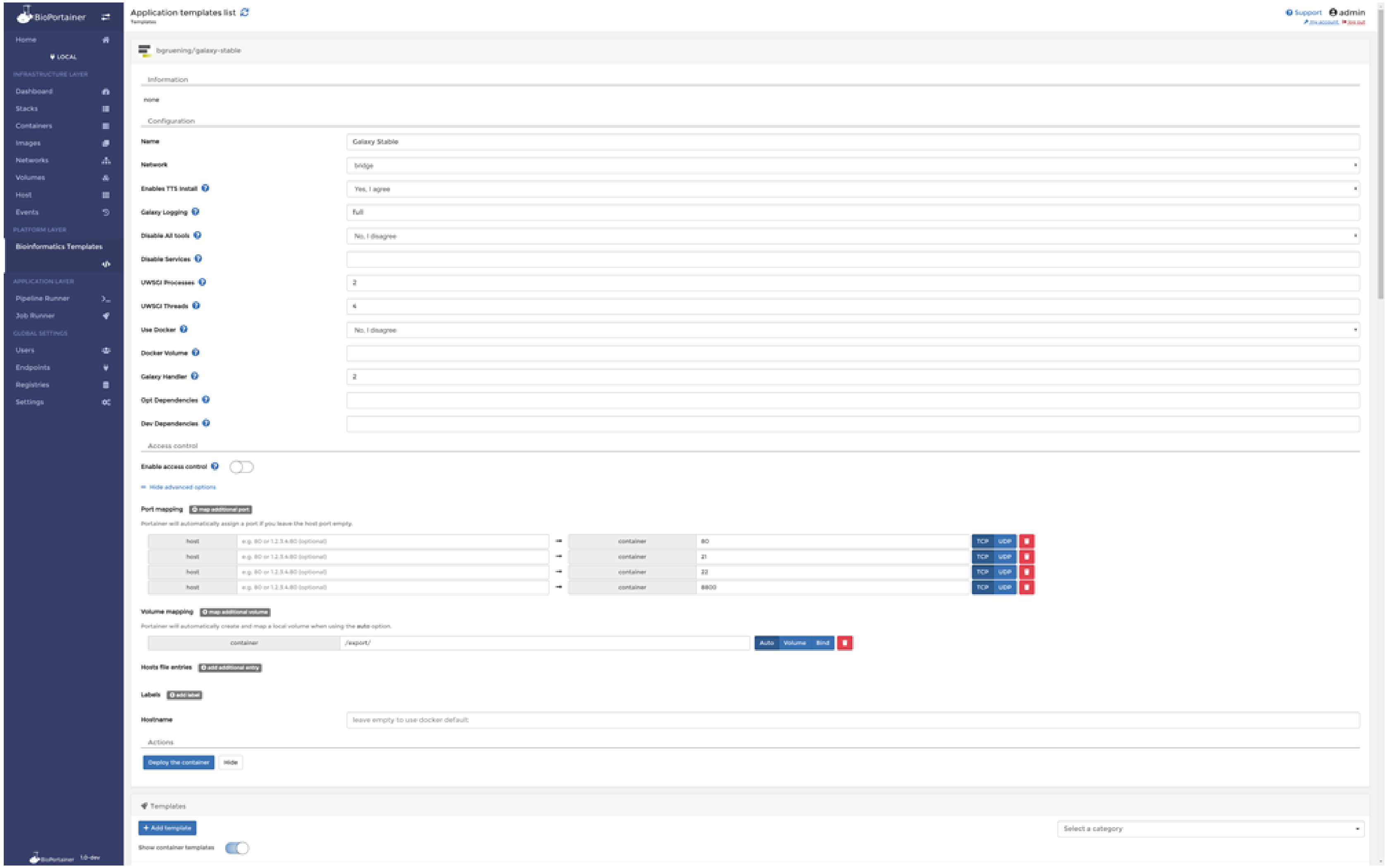

Figure S10. Galaxy Stable implementation form for Bioinformatics Templates menu. Using a simple and intuitive form, users can implement a local or remote instance of Galaxy, by configuring its main environment variables and advanced options. A total of 25 templates are provided for different versions of Galaxy tools.

PART 3 – LAUNCHING BIOINFORMATICS APPLICATIONS AND ANALYSES WITH ALTERNATIVA BPWB TOOLS

Once installed, BPWB can be used to launch bioinformatics analyses using tools readily available through the **BioPortainer Panel** main menu. The following sections will describe how each tool is launched, using four distinct case scenarios:

(1): An alignment of short Illumina reads against a reference genome, using the BWA aligner, launched with the aid of the **BioPortainer Console**.

(2): A full RNA-seq analysis, using the Tuxedo suite, launched with the aid of the **BioPortainer Job Runner**.

(3): A quality control analysis of Next Generation Sequencing (NGS) data, using the software FASTQC, launched with the aid of the **BioPortainer GUI-Runner**.

(4): Execution of two complex NextFlow pipelines (one designed to conduct a full RNA-seq analysis and other designed to conduct a Variant Calling analysis), launched with the aid of the **BioPortainer Pipeline Runner**. In these two case scenarios, launching has been performed both using the using the **BioPortainer Pipeline Runner GUI** and **Jupyter Notebook**. Finally, information regarding adaptation of NextFlow pipelines to evaluate user´s proprietary data is also provided (see details below).

Scenario 1: Launching Bioinformatics Applications with the BioPortainer Console

In this scenario, we demonstrate how the **BioPortainer Console** tool can be used to bioinformatics data analysis (using the console bash) to perform an alignment of short Illumina reads against a reference genome, using the BWA aligner. Details regarding the entire execution process are provided in the next section, along with a corresponding video file, available here. The exact time at which each action is displayed in the video is also provided (in parentheses) next to the instructions below.

For this analysis we will use the container generated in the **scenario 1 of Part 2**, which describes the deployment of a container carrying the **DugongClean CMD** platform. After deploying **DugongClean CMD**, the following steps must be performed to install BWA and launch the analysis:

1) Access the **Containers** option from the main menu. *[00:45]*

2) Selecting the **Console icon (> _)** (Figure S5) and clicking on **Connect**, user will gain access to the command bash of the installed container, being able to execute commands directly in the container (Figure S9). *[01:43]*

3) At the console, type the following command (Lines beginning with # are comments and do not need to be typed):

Install BWA through Conda *[01:51]* :

$ conda install bwa -y

Download the first RNA-Seq test library *[04:28]* :

$ wget
https://raw.githubusercontent.com/BioPortainer/Test/master/JobRunner/Example_Condition1.fastq

Download the second RNA-Seq test library *[04:38]* :

$ wget
https://raw.githubusercontent.com/BioPortainer/Test/master/JobRunner/Example_Condition2.fastq

Download the reference genome *[05:18]* :

$ wget
https://raw.githubusercontent.com/BioPortainer/Test/master/JobRunner/Mycoplasma_genitalium.fa

Create a BWA index in the genomic reference *[05:29]* :

$ bwa index Mycoplasma_genitalium.fa

Align the reads in the input file against the genomic reference *[05:40]* :

$ bwa aln -I -t 4 Mycoplasma_genitalium.fa Example_Condition1.fastq > out1_bwa.sai
$ bwa aln -I -t 4 Mycoplasma_genitalium.fa Example_Condition2.fastq > out2_bwa.sai

Convert the alignment into a .sam file *[05:58]* :

$ bwa samse Mycoplasma_genitalium.fa out1_bwa.sai Example_Condition1.fastq > out1_bwa.sam
$ bwa samse Mycoplasma_genitalium.fa out2_bwa.sai Example_Condition2.fastq > out2_bwa.sam

After the protocol runs (Figure S11), the output files are available in the working directory. The user can interact with the files through the console itself or through a file manager by navigating to the folder that was mapped in **step 8** in **the scenario 1 of Part 2** *[06:18]*.

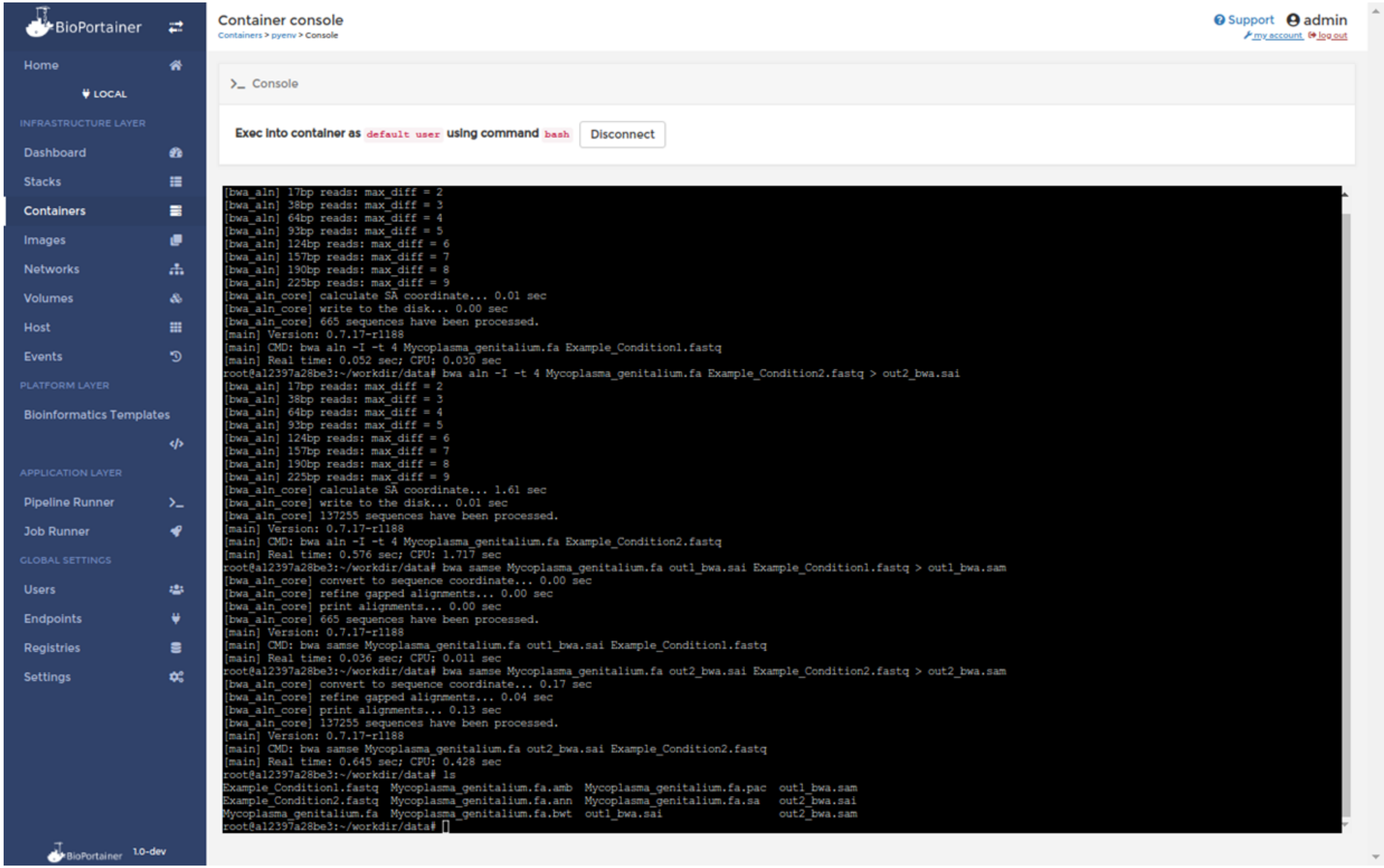

Figure S11. Alignment of short Illumina reads against a reference genome using BWA, launched with the aid of the BioPortainer Console. The BioPortainer Console represents the simplest alternative for launching analyses, normally involving a single bioinformatics tool. Once a container carrying a specific tool has been implemented, users only need to click the Console icon (>_).

Scenario 2: Launching Bioinformatics Applications with the BioPortainer Job Runner

In this scenario, we demonstrate how the **Job Runner** tool can be used in bioinformatics data analysis, by performing a full differential expression analysis using RNA-seq data, using the Tuxedo software suite. Details regarding the entire execution process are provided in the next section, along with a corresponding video file, available here. The exact time at which each action is displayed in the video is also provided (in parentheses) next to the instructions below.

After deploying and configuring **BPWB**, the following steps must be performed, through the **BioPortainer Panel** interface, to launch the analysis:

1) Access the **Job Runner** option from the main menu. *[00:02]*

2) Access the **Execute Job** option. *[00:03]*

3) Fill in the **image** field with the desired Docker image. For example, the Docker image **bioportainer/tuxedo:cpu** will be used, containing an installation of BWA, Bowtie2, Tophat and Cufflinks. *[00:04]*

4) Define the content of the script that will run during job through the **Web Editor** (Figure S12) or upload the file through the form available *[00:11]*. For the present execution, we used the following test script:

https://raw.githubusercontent.com/BioPortainer/Test/master/JobRunner/script.sh

5) Click in **Execute** button to run the analysis. *[00:37]*

NOTE: During the creation and execution of the analysis container, the **/home** folder of the host machine is automatically mapped to a folder named **/host** in the container. In addition, the supplied script will be copied to the **/tmp** folder which will also be configured as working directory.

6) Running the job can be tracked through the history available in the **Job Runner** option in the main menu. *[00:43]*

7) Clicking on *Job ID* will increase the number of lines that will be displayed in the log file during execution. *[00:47]*

NOTE: During Job execution, log entries will also be inserted into the **Events** option of the main menu, containing additional information regarding Docker images and container creation, among other processes. *[01:54]*

8) At the end of the Job a container with all inputs, outputs and analysis script will be available in the **Containers** option of the main menu. *[02:01]*

NOTE: Since the generated container does not have the **Interactive & TTY** parameters configured, it will be necessary to create a Docker image and a new container with these parameters to proceed with further analyses.

9) Click on the name of the generated container and in the **Create Image** option, to define the name of the backup image for the executed Job. *[02:43]*

10) After the backup Docker image is created, access the **Images** option from the menu and confirm its existence. *[03:08]*

NOTE: If necessary, the Docker image can be exported to a compressed file. *[03:30]*

11) Click the **Containers** option from the main menu and select **Add container** to configure a new container with the backup Docker image created. *[03:45]*

12) Fill in the container creation form with a name, name of the backup Docker image, and disable the options: **Always pull the images** and **Enable access control**. Enable the **Publish all exposed ports** option. *[03:47]*

13) On the **Commands & logging** tab, enable the **Interactive & TTY** option. *[04:07]*

14) On the **Volumes** tab, click on **Map additional volume** and add a mapping of type **Bind** to the **/root/workdir/data** directory *[04:12]*. Create a local directory that will be mapped to the container and enable the **Writable** option *[04:27]*.

15) Click the **Deploy the containe**r button to create the new container.

16) The new container will be available in the **Containers** option of the main menu *[04:51]* and can be accessed through the **Container Console** *[05:17]*.

NOTE: Users can manipulate the container through the console and direct the desired files between the container and host using the folder mapped in **step 14** *[05:24]*.

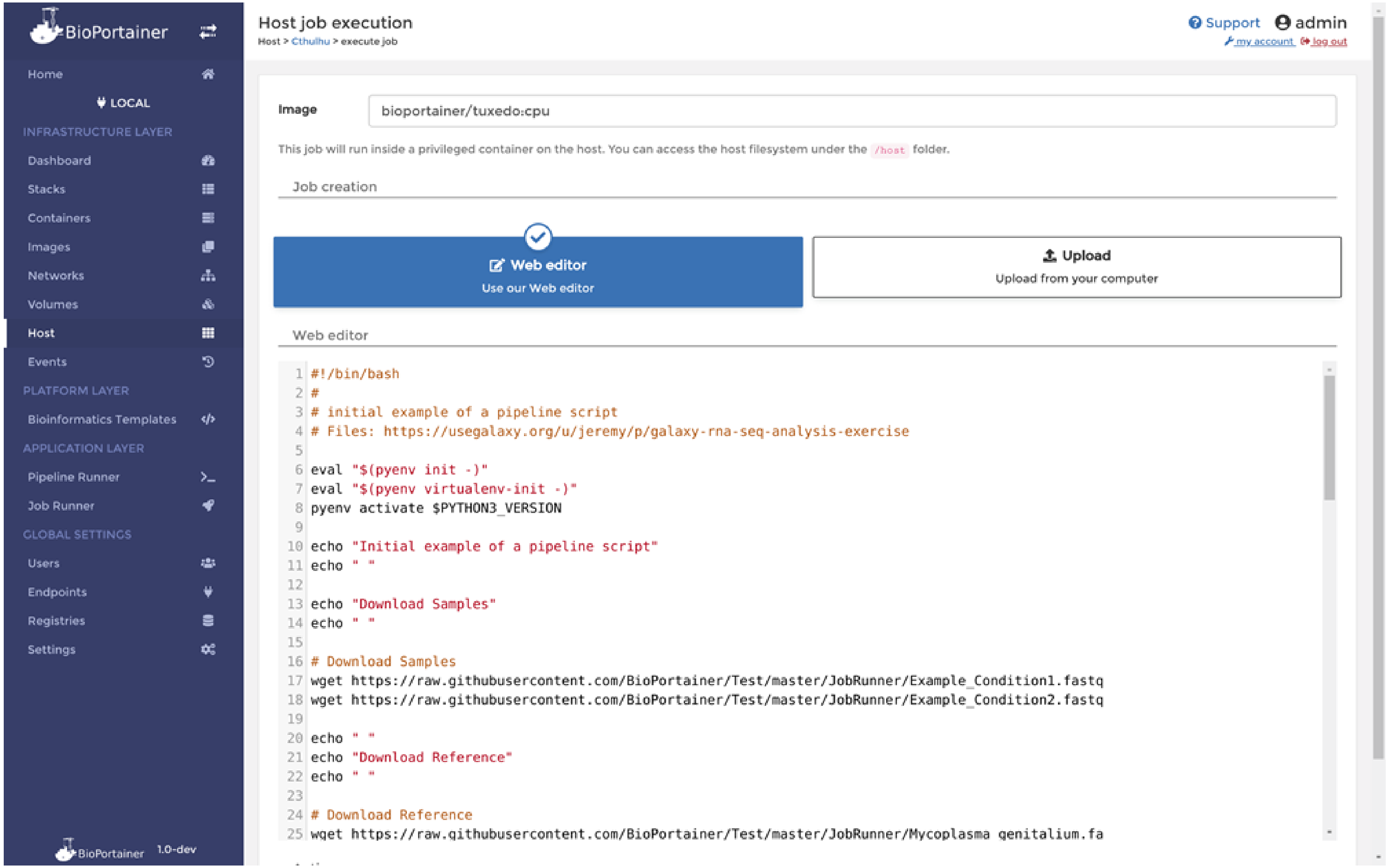

Figure S12. The Job Runner Execution Form. Through an easy-to-use form, users can use a predefined Docker image and a script to execute local processes in the host machine, which will be encapsulated in a Docker container and later converted into an image that can be easily exported to and shared with other researchers.

Scenario 3: Launching Bioinformatics Applications with the BioPortainer GUI-Runner

In this scenario, we demonstrate how the **BioPortainer GUI-Runner** tool can be used to bioinformatics data analysis. The **BioPortainer GUI-Runner** runs in a Docker container, carrying a web interface (built in Python) based on preconfigured JSON files. Each bioinformatics software available from the **BioPortainer Gui-Runner** has been specifically developed from a basic JSON model (available here) to allow users to interact with all the main parameters of their respective software, through a web-based GUI. Further details on eachtools and their parameters are available in **Table S2** (available at the end of this manual), as well in specific documentation at the BioPortainer Workbench homepage. We currently have 22 bioinformatics categories of tools at the BioPortainer Workbench repository, with more than 100 bioinformatics tools fully adapted to be used with the **BioPortainer GUI-Runner**. For the construction of a new **GUI-Runner** container, users should use the Docker image **bioportainer/gui-runner** as Dockerfile's FROM and perform the installation of the desired tools through the Conda manager. After installation, a JSON file should be created based on our basic JSON model and made available in the **/BioGUI/conf/runners** folder. Users can also provide a script file in the **/BioGUI/conf/scripts** folder to expedite futher analyses. The Dockerfile of the FASTQC tool can be used as a model for building new **GUI-Runner** tools.

As mentioned above, we will use the FASTQC tool for testing this scenario with an NGS dataset. Details regarding the entire execution process are provided in the next section, along with a corresponding video file, available here. The exact time at which each action is displayed in the video is also provided (in parentheses) next to the instructions below.

After deploy and configuration **BioPortainer Panel**, the following steps must be performed for the analysis:

1) Visit the **Bioinformatics Templates** option in the **BioPortainer Panel** main menu to see the available templates. *[00:02]*

2) Select the **Show container templates** option.

3) Select **BioPortainer GUI-Runner.** *[00:06]*

4) Select **BioPortainer GUI-Runner - FASTQC** to configure the installation form. *[00:14]*

5) Enter a name for the container (for example: FASTQC). *[00:17]*

6) Select **Show Advanced Options.** *[00:22]*

7) Create a local directory for mapping to the container directory. *[00:30]*

8) Map container volume to a folder created on the host. *[00:45]*

9) Create the container by clicking the **Deploy Container** button. *[00:59]*

10) After creating the **GUI-Runner** container, go to the **Containers** option in the main menu. *[01:05]*

11) In **Published Ports** option, click on the container 5000 port mapping (If access is not by localhost, replace IP 0.0.0.0 with the IP or domain of the remote server). *[01:12]*

NOTE: The GUI-Runner menu provides some common options for all configured tools:

(i) Clone GIT Repository: allows cloning of GIT repositories in workdir;
(ii) Workdir Activate: allows access to the directory of a cloned GIT repository;
(iii) GIT Local Repository Remove: allows you to remove a cloned GIT repository.

12) In **GUI-Runner** interface, select the **FASTQC** option from the menu. *[01:27]*

13) The run form provides fields corresponding to the default FASTQC parameters available from the command line. For more information on each of the parameters, simply enable the Help check box *[01:38]* or place the mouse over the desired field *[02:06]*.

14) In the **Input Files** field, select the FASTQ file for analysis. A test file is available here. *[02:20]*

15) Select the output folder in the **Output Dir** field. The current folder will be the output directory if nothing is changed. *[02:22]*

NOTE: All other fields are optional.

16) Click on the **Execute** button to run FASTQC. *[02:24]*

After the protocol runs (Figure S13), the output files are available in the working directory. The user can interact with the files through the console itself or through a file manager by navigating to the folder that was mapped in **step 8** in **scenario 3** of **Part 3** *[02:36].*

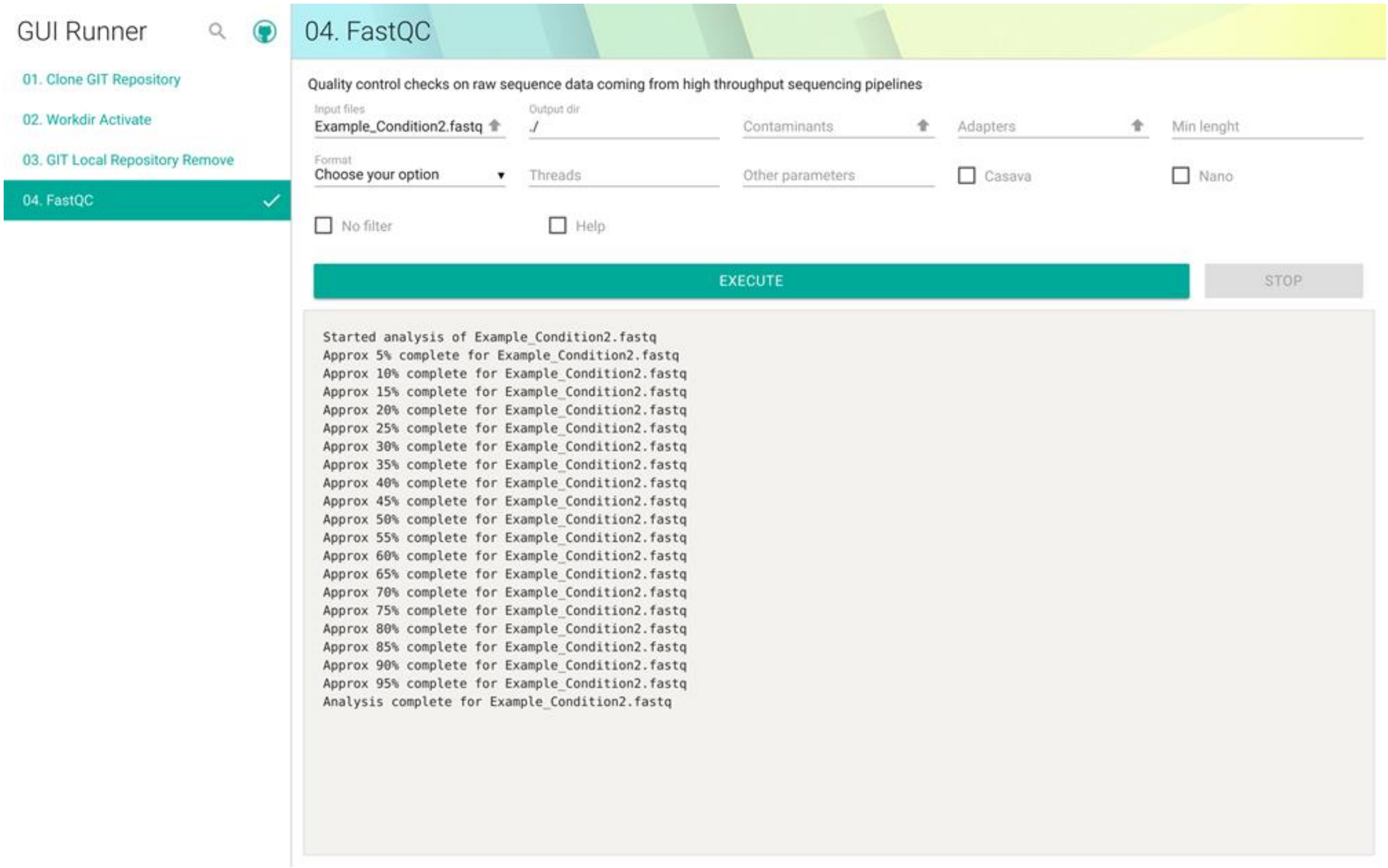

Figure S13. FASTQC analysis using GUI Runner. Here the FASTQC tool was implemented in a web interface in order to facilitate its use: there is no need to type all the commands, but users can click on a parameter to turn it on/off, select a parameter's option from a list, and even click on a parameter to upload an input file, for example.

Scenario 4: Launching Bioinformatics Applications with the BioPortainer Pipeline Runner

Bioinformatics applications are often highly complex and require the use of multiple analytical steps, executed by a variety of software, which must be coordinately executed through a series of commands with the aid of workflows and pipelines. Given the inherent complexity of pipeline commands, users not fully familiarized with Command Line-based Interfaces (CLIs) often face great difficulties to implement analyses of this sort. Thus, the **BioPortainer Pipeline Runner** has been developed to provide less experienced users with a friendlier alternative to conduct data analyses using pipelines and workflows.

Although pipelines from different sources may function well, when executed with the aid of the **BioPortainer Pipeline Runner**, this tool has been majorly developed to run pipelines developed by the NextFlow and NF-core projects, given their adequate integration with Docker environments and standardized structure, which makes them easier to edit/adapt to the specific needs of different users (see below).

Thus, in this scenario, we demonstrate how the **BioPortainer Pipeline Runner** tool can be used in bioinformatics data analysis, **by demonstrating the launching of two different pipelines** (one designed to execute **RNA-seq analysis** and another designed to execute **Variant Call analysis**). Both pipelines have been obtained from the NextFlow repository and, as such, are pre-configured to execute their analyses with test datasets. Instructions on how to run NextFlow pipelines with alternative datasets (user´s, for example) are provided at the end of this manual (see below).

Execution of both pipelines is demonstrated in two stages: (i) using the **BioPortainer Pipeline Runner GUI** (Figures S14 and S15) and (ii) using the Jupyter Notebook (Figure S1).

4.1: Running NextFlow pipelines with the aid of the BioPortainer Pipeline Runner GUI

One of the ways to run NextFlow pipelines through the BioPortainer Pipeline Runner is through its web GUI, available on port 5000. After deploying and configuring **BPWB**, the following steps must be performed to launch the analysis. Details regarding the entire execution process are provided in the next section, along with a corresponding video file, available here. The exact time at which each action is displayed in the video is also provided (in parentheses) next to the instructions below:

1) Access to the **BioPortainer Pipeline Runner** can be accomplished in different ways:

(i) by the address **http://localhost:5000** (or replacing localhost by the domain or IP of the remote server); *[00:06]*
(ii) through the **Containers** option of the **BioPortainer Panel** main menu and clicking on the **5000:5000** port available in the **Published Ports** option; *[00:17]* or
(iii) by clicking on the **Pipeline Runner** option in the **BioPortainer Panel** main menu and following the described steps. *[00:27]*

2) After accessing the web GUI of **Pipeline Runner** a few options are presented in the main menu *[00:32]* :

(i) Clone GIT Repository: allows copying of GIT repositories into workdir; *[00:34]*
(ii) GIT Local Repository Activate: allows access to the directory of a cloned GIT repository; *[00:58]*
(iii) GIT Local Repository Remove: allows to remove a cloned GIT repository; *[01:02]*
(iv) GIT Local Repository Update: updates a cloned GIT repository; *[01:03]*
(v) NextFlow Pipeline Runner: Execute a pipeline project; *[01:05]*
(vi) NextFlow Pipeline Logs: Print executions log and runtime info; *[01:32]*
(vii) NextFlow Pipeline Clean: Clean up project cache and work directories; *[01:34]*
(viii) NextFlow Pipeline Config: Print a project configuration; *[01:36]*
(ix) NextFlow Pipeline Info: Print project and system runtime information; *[01:37]*
(x) NextFlow Pipeline List: List all downloaded projects; *[01:39]*
(xi) NextFlow Pipeline View: View project script file(s); *[01:40]*
(xii) NextFlow Pipeline Pull: Download or update a project; *[01:42]*
(xiii) NextFlow Pipeline Drop: Delete the local copy of a project. *[01:43]*

NOTE: All menu options named NextFlow correspond to the standard tool commands ported to the **Pipeline Runner** GUI web. More details on each of their attributes can be obtained in the extensive documentation of NextFlow, or through the **Help** check box.

3) Select a **GIT Clone Repository** *[01:45]* and download the two pipelines used for our test:

(i) Simple RNA-seq pipeline using Samtools and Tuxedo; and
(ii) Consensus assembly and variant calling workflow.

The two pipelines are available at the Official Repository of the **NextFlow Project**, along with other pipelines for different bioinformatics analysis.

4) Enter the repository address in the **GIT Repository** field and click the **Execute** button (the other parameters are optional and, in most cases, unnecessary). *[02:00]*

5) Repeat the process for the two pipelines. *[02:25]*

6) Click the **GIT Local Repository Activate** menu option and select the RNA-Seq pipeline (rnatoy) by clicking **Execute**. *[02:38]*

7) Click the **NextFlow Pipeline Runner** menu option to run the RNA-Seq pipeline (rnatoy). *[02:43]*

NOTE: All forms options correspond to the NextFlow parameters available in your command line interface. Details about each field can be obtained through the documentation of NextFlow or through the **Help** check box.

8) Fill in the **NextFlow Project** field with the name of the **.NF file** available in the pipeline used. By default, the run file will be **main.nf**. Details about which file to use should be obtained from the pipeline documentation chosen. In our example, we will use the **main.nf** file. *[02:46]*

9) Define a folder that will receive the files generated during execution of the pipeline through the **Nextflow WorkDir** field. If the field is not changed, a directory named **work** will be created. *[02:49]*

NOTE: All other fields are optional. The DAG Enable, HTML Report, Timeline Report, and Tracing Report fields are enabled by default. Details on these fields can be obtained here.

10) Click the **Execute** button to run the pipeline. *[02:56]*

11) After running the RNA-Seq pipeline, click again on the **GIT Local Repository Activate** option from the main menu, select the Call Variants pipeline (nmdp-flow) and click **Execute**. *[03:27]*

12) Click the **NextFlow Pipeline Runner** menu option to run the Call Variants pipeline (nmdp-flow). *[03:33]*

13) Set the **NextFlow Project** field to **main.nf** and do not change the other fields. *[03:35]*

14) Click the **Execute** button to run the pipeline. *[03:39]*

After the protocol runs, the output files are available in the **bioportainer_workdir** directory. The user can interact with the files through the console itself or through a file manager by navigating to the folder **bioportainer_workdir**. *[04:06]*

- 4.2: Running NextFlow pipelines through the Pipeline Runner with the aid of the Jupyter Notebook

One of the ways to run Nextflow pipelines through the **BioPortainer Pipeline Runner** is through its **Jupyter Notebook** available on port 8888. After deploying and configuring **BPWB**, the following steps must be performed for the analysis. Details regarding the entire execution process are provided in the next section, along with a corresponding video file, available here. The exact time at which each action is displayed in the video is also provided (in parentheses) next to the instructions below:

1) Access to the **BioPortainer Pipeline Runner** – **Jupyter Notebook** can be accomplished in different ways:

(i) by the address **http://localhost:8888** (or replacing localhost by the domain or IP of the remote server); *[00:03]*
(ii) through the **Containers** option of the **BioPortainer Panel** main menu and clicking on the **8888:8888** port available in the **Published Ports** option; *[00:12]* or
***(iii)** by clicking on the **Pipeline Runner** option in the **BioPortainer Panel** main menu and following the described steps. [00:21]*

2) After accessing the **Jupyter Notebook** interface, an access token is required for authentication. To get the access token follow the steps below:

(i) Access the **BioPortainer Panel** *[00:28]*
(ii) Click the **Containers** option in the main menu *[00:29]*
(iii) Click on the **Console (>_)** button corresponding to **Pipeline Runner** container; *[00:33]*
(iv) After connecting to console, enter the command: *jupyter-notebook list [00:45]*
(v) Copy the full link of Jupyter as well as the first access token. *[00:47]*

3) After access with the token, the Jupyter interface will be available for the creation or use of notebooks. *[00:49]*

4) For this scenario, we have provided an example notebook containing the pipelines used in **Scenario 4.1** *[01:08]*. The Jupyter service that ships with the **BioPortainer Pipeline Runner** contains the **bash kernel**, allowing you to run any NextFlow command directly from the created notebook.

5) In the Jupyter interface, click on the **Upload** button, at the top-right and select the downloaded file. Then press the blue **Upload** button. Now, click again on the uploaded notebook to open another browser tab to run it. *[01:23]*

6) The example notebook is divided in **Download Workflows** and **Execute Workflows**. *[01:39]*

7) Click on **Cell**, at the top menu, then click on **Run All**, to run all the commands at once. *[01:47]*

After the protocol runs, the output files are available in the **bioportainer_workdir** directory *[03:04]*. The user can interact with the files through the container's own console, or through an operating system file manager. In addition, Jupyter provides a native manager that allows interaction with the created files *[03:34]*.

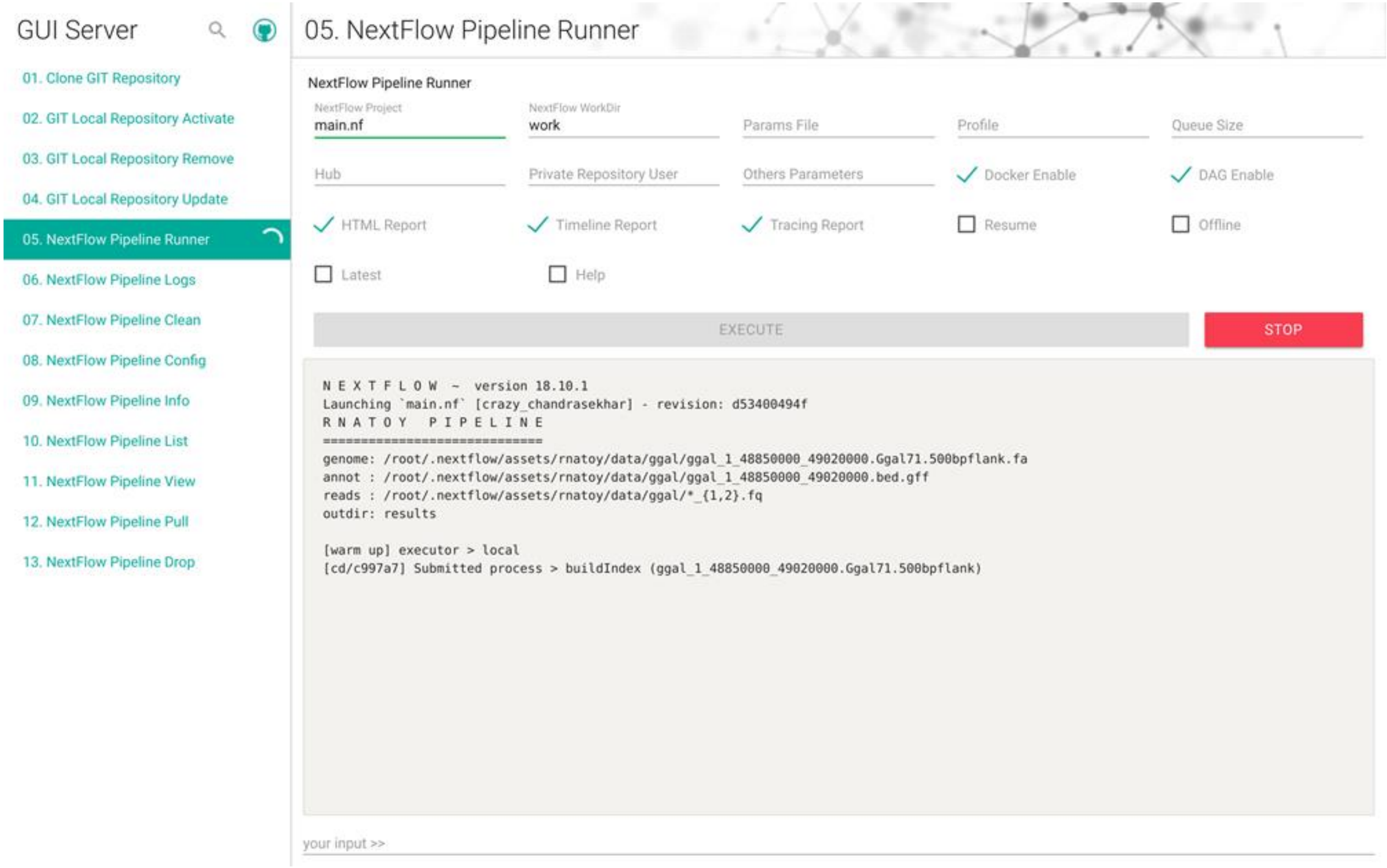

Figure S14. BioPortainer Pipeline Runner GUI. An overview of the NextFlow Pipeline Runner form, shown while executing the RNA-Seq pipeline nextflow-io/rnatoy

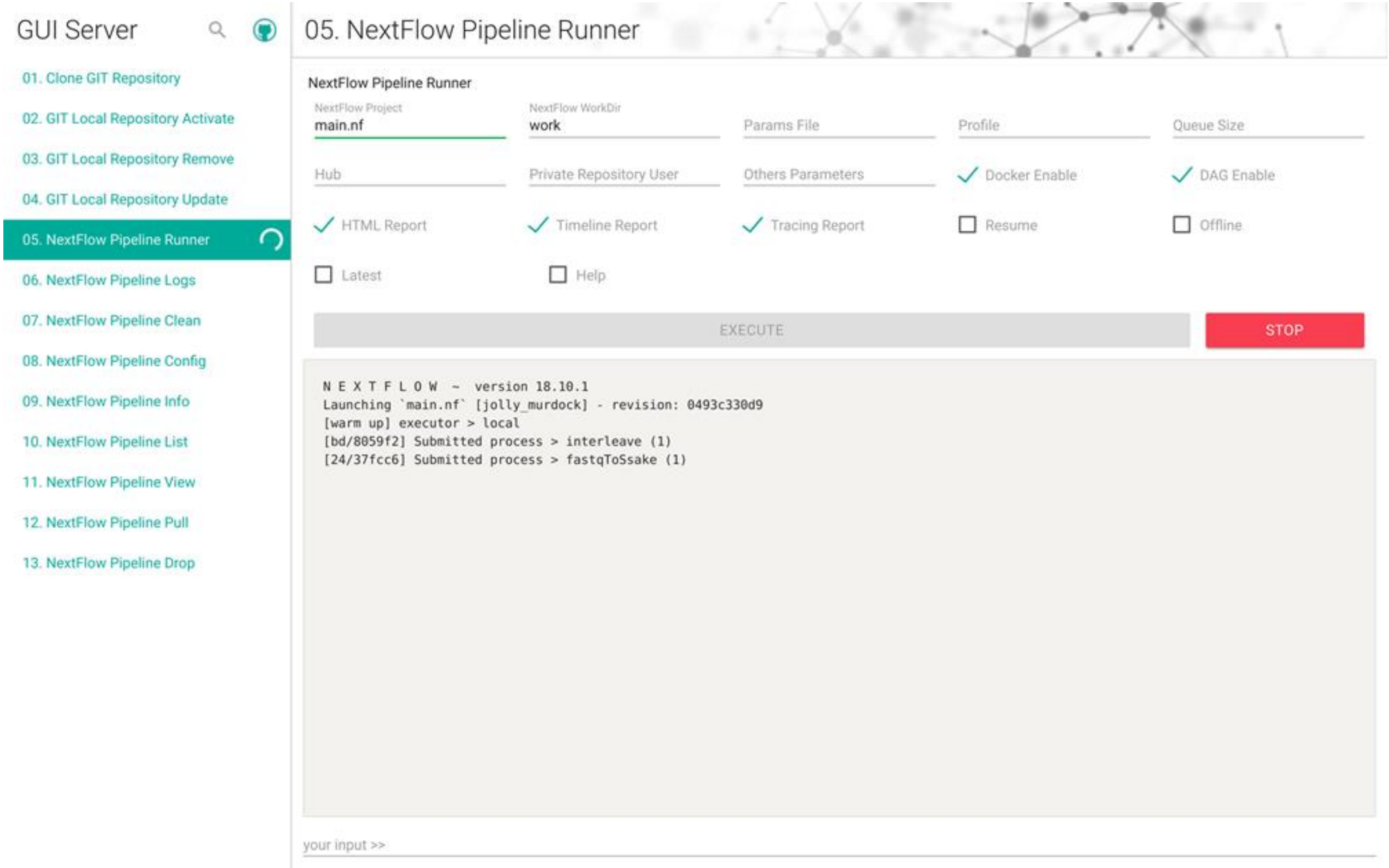

Figure S15. BioPortainer Pipeline Runner GUI. An overview of the NextFlow Pipeline Runner form, shown while executing the Variant Call pipeline nextflow-io/flow

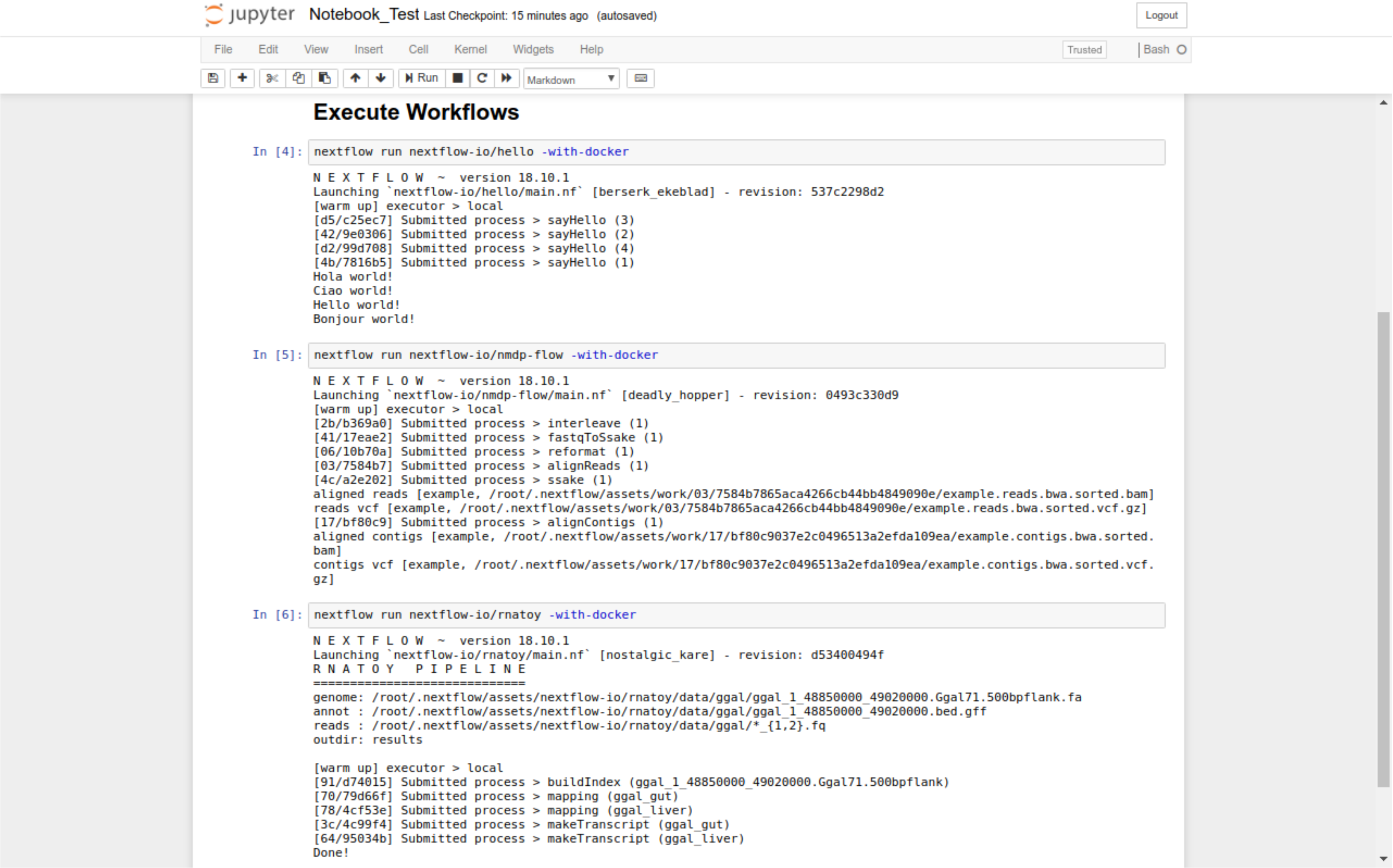

Figure S16. Download and Execution (through a Jupyter notebook file) of the official NextFlow pipelines for analysis of RNA-Seq (nextflow-io/rnatoy) and Variant Call (nextflow-io/flow)

4.3: Analyzing proprietary data with NextFlow Pipelines

As mentioned above, pipelines developed by the NextFlow and NF-core projects are made available with a pre-defined configuration to run specific test data, so users can perform simulations and verify their effectiveness under different installation environments.

However, to use such pipelines to run their own data, users must promote a few modifications in the pipelines´ parameters. Fortunately, NextFlow pipelines are written under a highly standardized structure, so it is not difficult to promote the necessary changes using any text editor available. In fact, extensive documentation, available at the NextFlow webpage provide simple or detailed examples on how users can modify, adapt and even create new pipelines to run their own proprietary data.

Thus, the final part of this manual will be dedicated to demonstrate the adaptation of the nextflow/rnatoy pipeline (an RNA-Seq-dedicated workflow, available from the NextFlow Project repository) to perform differential gene expression analyses using RNA-seq libraries obtained from *Drosophyla melanogaster* flies, carrying two distinct backgrounds. The NGS data have is provided by Melbourne Bioinformatics and is used in conjunction with annotation data obtained from Ensembl. All data used herein are available at the BioPortainer Workbench Project homepage.

1) Access to the **BioPortainer Pipeline Runner**.

2) Select a **GIT Clone Repository** and download the **nextflow/rnatoy**, as described in **Scenario 4.1**.

3) Access the **bioportainer_workdir** directory through your file manager and navigate to the root of the pipeline folder.

4) Change permission of the folder, so your user can save the files. Run on Linux bash:

$ sudo chown -R $USER:$USER bioportainer_workdir

5) Open the **main.nf** file with a text editor of your choice.

6) Look for the lines describing the pipeline´s parameters. Given the standardized structure of NextFlow pipelines, these lines are easily found, following the initial header. In the case of the nextflow/rnatoy pipeline, this section is shown below and labeled in magenta:

```
/*
 * Defines some parameters in order to specify the refence genomes
 * and read pairs by using the command line options
 */
params.reads = "$baseDir/data/ggal/*_{1,2}.fq"
params.annot = "$baseDir/data/ggal/ggal_1_48850000_49020000.bed.gff"
params.genome = "$baseDir/data/ggal/ggal_1_48850000_49020000.Ggal71.500bpflank.fa"
params.outdir = 'results'
```

These parameters represent the following:

(i) params.reads: defines the folder where the sample files are located, along with their names and formats (**_1,2} .fq*). Note that the variable has Paired-End files (1,2).
(ii) params.annot: defines the location, name and format of the GFF annotation file.

(iii) params.genome: defines the location, name and format (FASTA) of the reference genome.
(iv) params.outdir: defines the folder that will store the output files.

NOTE: Other pipelines may have different parameter lines, when compared to the ones described herein. Information regarding which parameters must be changed to adapt a given pipeline to run your proprietary data can be found at the pipeline´s specific page, at the NextFlow and Nf-Core repositories.

7) Click the **GIT Local Repository Activate** menu option and select the RNA-Seq pipeline (rnatoy).

8) Select a **GIT Clone Repository** and download the repository:

https://github.com/BioPortainer/Test

9) Access the **bioportainer_workdir** directory through your file manager and copy the folder **Test/Pipeline-Runner/samples** to **bioportainer_workdir/rnatoy**

10) Edit the **main.nf** file of the **nextflow/rnatoy** pipeline and change the parameters as follows:

```
/*
 * Defines some parameters in order to specify the refence genomes
 * and read pairs by using the command line options
 */
params.reads = "$baseDir/data/samples/*_{R1,R2}.fastq"
params.annot = "$baseDir/data/samples/ensembl.chr4.gtf"
params.genome = "$baseDir/data/samples/ensembl.chr4.fa"
params.outdir = 'results'
```

11) Access to the **BioPortainer Pipeline Runner**.

12) Click the **NextFlow Pipeline Runner** menu option to run the RNA-Seq pipeline (rnatoy).

13) Set the **Project Name** field to **main.nf** and do not change the other fields.

14) Click the **Execute** button to run the pipeline.

After the protocol runs, the output files are available in the **bioportainer_workdir** directory. Note that the GTF files named **KO_01** (for *knockout* flies) and **WT_01** (for *wild-type* flies) were created within the results folder, demonstrating that the analysis was performed with the new files provided with the new parameters settings.

Table S1 – Platforms and Basic tools available through the BioPortainer Workbench platform options

Plarforms	Template category / tool	Description
BioPortainer CPU	BioPortainer Pipeline Runner	BioPortainer Image of Pipeline Runner platfom
BioPortainer CPU	BioPortainer Base - CPU	BioPortainer Base image of CPU platform
BioPortainer CPU	BioPortainer SciEnv - CPU	BioPortainer Image of SciEnv for CPU
BioPortainer CPU	BioPortainer PyEnv - CPU	BioPortainer Image of PyEnv for CPU
BioPortainer CPU	BioPortainer SupervisorD - CPU	BioPortainer Image of SupervisorD for CPU
BioPortainer GPU	BioPortainer Base - GPU	BioPortainer Base image of GPU platform
BioPortainer GPU	BioPortainer SciEnv - GPU	BioPortainer Image of SciEnv for GPU
BioPortainer GPU	BioPortainer PyEnv - GPU	BioPortainer Image of PyEnv for GPU
BioPortainer GPU	BioPortainer SupervisorD - GPU	BioPortainer Image of SupervisorD for GPU
BioPortainer GUI-Runner	BioPortainer GUI-Runner - CPU	Main template of BioPortainer GUI-Runner
BioPortainer GUI-Runner	BioPortainer GUI-Runner - FASTQC	BioPortainer container of GUI-Runner implemented with the tool FASTQC
BioPortainer GUI-Runner	BioPortainer GUI-Runner - TOPHAT	BioPortainer container of GUI-Runner implemented with the tool TOPHAT
BioPortainer GUI-Runner	BioPortainer GUI-Runner - BWA	BioPortainer container of GUI-Runner implemented with the tool BWA
BioPortainer GUI-Runner	BioPortainer GUI-Runner - CDHIT	BioPortainer container of GUI-Runner implemented with the tool CDHIT
BioPortainer GUI-Runner	BioPortainer GUI-Runner - Clustal-O	BioPortainer container of GUI-Runner implemented with the tool Clustal-O
BioPortainer GUI-Runner	BioPortainer GUI-Runner - Cufflinks	BioPortainer container of GUI-Runner implemented with the tool Cufflinks
BioPortainer GUI-Runner	BioPortainer GUI-Runner - Deeptools	BioPortainer container of GUI-Runner implemented with the tool Deeptools
BioPortainer GUI-Runner	BioPortainer GUI-Runner - Fasta-Splitter	BioPortainer container of GUI-Runner implemented with the tool Fasta-Splitter
BioPortainer GUI-Runner	BioPortainer GUI-Runner - Fastq-join	BioPortainer container of GUI-Runner implemented with the tool Fastq-join

BioPortainer GUI-Runner	BioPortainer GUI-Runner - Fastq-tools	BioPortainer container of GUI-Runner implemented with the tool Fastq-tools
BioPortainer GUI-Runner	BioPortainer GUI-Runner - Fastx-Toolkit	BioPortainer container of GUI-Runner implemented with the tool Fastx-Toolkit
BioPortainer GUI-Runner	BioPortainer GUI-Runner - Flux-simulator	BioPortainer container of GUI-Runner implemented with the tool Flux-simulator
BioPortainer GUI-Runner	BioPortainer GUI-Runner - Gatk	BioPortainer container of GUI-Runner implemented with the tool Gatk
BioPortainer GUI-Runner	BioPortainer GUI-Runner - GlimmerHMM	BioPortainer container of GUI-Runner implemented with the tool GlimmerHMM
BioPortainer GUI-Runner	BioPortainer GUI-Runner - Gmap-fusion	BioPortainer container of GUI-Runner implemented with the tool Gmap-fusion
BioPortainer GUI-Runner	BioPortainer GUI-Runner - Humann2	BioPortainer container of GUI-Runner implemented with the tool Humann2
BioPortainer GUI-Runner	BioPortainer GUI-Runner - Kallisto	BioPortainer container of GUI-Runner implemented with the tool Kallisto
BioPortainer GUI-Runner	BioPortainer GUI-Runner - Khmer	BioPortainer container of GUI-Runner implemented with the tool Khmer
BioPortainer GUI-Runner	BioPortainer GUI-Runner - miRDeep2	BioPortainer container of GUI-Runner implemented with the tool miRDeep2
BioPortainer GUI-Runner	BioPortainer GUI-Runner - Trim-galore	BioPortainer container of GUI-Runner implemented with the tool Trim-galore
Dugong Platform	DugongCMD	Desktop-as-a-Service, providing access to software from BioConda, LinuxBrew and Jupyter Notebook, among other features
Dugong Platform	DugongGUI XFCE4	Desktop-as-a-Service, providing access to software from BioConda, LinuxBrew and Jupyter Notebook, among other features
Dugong Platform	DugongGUI IceWM	Desktop-as-a-Service, providing access to software from BioConda, LinuxBrew and Jupyter Notebook, among other features
Dugong Platform	DugongClean CMD	Desktop-as-a-Service, providing access to software from BioConda, LinuxBrew and Jupyter Notebook, among other features
Dugong Platform	DugongClean XFCE4	Desktop-as-a-Service, providing access to software from BioConda, LinuxBrew and Jupyter Notebook, among other features

Dugong Platform	DugongClean IceWM	Desktop-as-a-Service, providing access to software from BioConda, LinuxBrew and Jupyter Notebook, among other features
Galaxy Platform	Galaxy Stable	Galaxy Docker Image Stable.
Galaxy Platform	Galaxy: UseGalaxy.org	Galaxy Docker repository with tools from usegalaxy.org (UseGalaxy.org Platform).
Galaxy Platform	Galaxy: Workflow4Metabolomics	Galaxy Docker Image Workflow4Metabolomics Platform.
Galaxy Platform	Galaxy: RNA workbench	Galaxy Docker Image RNA workbench Platform.
Galaxy Platform	Galaxy: NCBI-Blast	Galaxy Docker Image NCBI-Blast Platform.
Galaxy Platform	Galaxy: Ballaxy	Galaxy Docker Image Ballaxy Platform.
Galaxy Platform	Galaxy: DeepTools	Galaxy Docker Image DeepTools Platform.
Galaxy Platform	Galaxy: ChIP-exo Workbench	Galaxy Docker Image ChIP-exo Workbench Platform.
Galaxy Platform	Galaxy: Proteomics	Galaxy Docker Image Proteomics Workbench Platform.
Galaxy Platform	Galaxy: NGS Preprocessing	Galaxy Docker Image NGS Preprocessing Platform.
Galaxy Platform	Galaxy: MPI for Evolutionary Biology	Galaxy Docker Image MPI for Evolutionary Biology Platform.
Galaxy Platform	Galaxy: Cheminformatics workbench	Galaxy Docker Image Cheminformatics workbench Platform.
Galaxy Platform	Galaxy: Epigenetics workbench	Galaxy Docker Image Epigenetics workbench Platform.
Galaxy Platform	Galaxy: Machine Learning	Galaxy Docker Image Machine Learning Platform.
Galaxy Platform	Galaxy: Molecular Phylogenetics	Galaxy Docker Image Molecular Phylogenetics Platform.
Galaxy Platform	Galaxy: Protease Predictions	Galaxy Docker Image Protease Predictions Platform.
Galaxy Platform	Galaxy: Metagenomics Workbench	Galaxy Docker Image Metagenomics Workbench Platform.
Galaxy Platform	Galaxy: DESeq2 Workbench	Galaxy Docker Image DESeq2 Workbench Platform.
Galaxy Platform	Galaxy: Sequence Tools	Galaxy Docker Image Sequence Tools Platform.
Galaxy Platform	Galaxy: Exome Seq	Galaxy Docker Image Exome Seq Platform.
Galaxy Platform	Galaxy: RNA Structural Analysis	Galaxy Docker Image RNA Structural Analysis Platform.
Galaxy Platform	Galaxy: Genome Annotation	Galaxy Docker Image Genome Annotation Platform.
Galaxy Platform	Galaxy: Image Analysis	Galaxy Docker Image Imaging Analysis Platform.
Galaxy Platform	Galaxy: MUMmer 3	Galaxy Docker Image MUMmer 3 Platform.
Galaxy Platform	Galaxy: RNA-Seq	Galaxy Docker Image RNA-Seq Platform.
Galaxy Tools	Galaxy Tools: IPython Notebook	Docker IPython Container
Galaxy Tools	Galaxy Tools: Jupyter Notebook	Docker Jupyter Container

Galaxy Tools	Galaxy Tools: Planemo	Docker Planemo Container
Jupyter Notebook	Jupyter Notebook: Base	Small base image for Jupyter Notebook.
Jupyter Notebook	Jupyter Notebook: JupyterHub	JupyterHub: A multi-user server for Jupyter notebooks.
Jupyter Notebook	Jupyter Notebook: SingleUser	JupyterHub: A single-user server for Jupyter notebooks.
Jupyter Notebook	Jupyter Notebook: Minimal	Minimal Jupyter Notebook.
Jupyter Notebook	Jupyter Notebook: Scientific Python	Jupyter Notebook Scientific Python Stack.
Jupyter Notebook	Jupyter Notebook: All Spark	Jupyter Notebook Python, Scala, R, Spark, Mesos Stack.
Jupyter Notebook	Jupyter Notebook: Data Science	Jupyter Notebook Data Science.
Jupyter Notebook	Jupyter Notebook: Tensorflow	Jupyter Notebook Scientific Python Stack Tensorflow.
Jupyter Notebook	Jupyter Notebook: Viewer	Jupyter Notebook Viewer.
GUIdock Platform	GUIdock	Delivering GUI Display from Docker.
GUIdock Platform	GUIdock: noVNC	Delivering GUI Display from Docker.
R Platform	R: Base	R is a system for statistical computation and graphics.
R Platform	R: RStudio	RStudio Server image.
R Platform	R: Shiny	Shiny Server image.
Bioconductor Platform	Bioconductor: Base	Bioconductor: Base image.
Bioconductor Platform	Bioconductor: Core	Bioconductor: Core image.
Bioconductor Platform	Bioconductor: Flow Cytometry	Bioconductor: Flow Cytometry image.
Bioconductor Platform	Bioconductor: Microarray Packages	Bioconductor: Microarray Packages image.
Bioconductor Platform	Bioconductor: Sequencing Packages	Bioconductor: Sequencing Packages image.
Bioconductor Platform	Bioconductor: Proteomics Packages	Bioconductor: Proteomics Packages image.
Bioconductor Platform	Bioconductor: Metabolomics Packages	Bioconductor: Metabolomics Packages image.
BioContainer Platform	BioContainer: Base	An open-source and community-driven framework for software standardization.

Table S2 – Tools available for launching bioinformatics applications with the aid of the BioPortainer GUI Runner

Category	Tool	Description	Source
GIT	PACK	Git is a fast, scalable, distributed revision control system with an unusually rich command set that provides both high-level operations and full access to internals	https://git-scm.com/
GIT	Activate Local Repository	Choose your working repository	https://git-scm.com/
GIT	Clone Repository	Clone a repository into a new directory	https://git-scm.com/
GIT	Remove Local Repository	Remove files from the working tree and from the index	https://git-scm.com/
GIT	Update Local Repository	Fetch from and integrate with another repository or a local branch	https://git-scm.com/
Bowtie1	bowtie1	Ultrafast, memory-efficient short read aligner geared toward quickly aligning large sets of short DNA sequences (reads) to large genomes. It aligns 35-base-pair reads to the human genome at a rate of 25 million reads per hour on a typical workstation	http://bowtie-bio.sourceforge.net/index.shtml
Bowtie2	bowtie2	Ultrafast and memory-efficient tool for aligning sequencing reads to long reference sequences. It is particularly good at aligning reads of about 50 up to 100s or 1,000s of characters, and particularly good at aligning to relatively long (e.g. mammalian) genomes	http://bowtie-bio.sourceforge.net/bowtie2/index.shtml
Bwa tools	PACK	BWA is a software package for mapping low-divergent sequences against a large reference genome, such as the human genome. It consists of three algorithms: BWA-backtrack, BWA-SW and BWA-MEM. The first algorithm is designed for Illumina sequence reads up to 100bp, while the rest two for longer sequences ranged from 70bp to 1Mbp. BWA-MEM and BWA-SW share similar features such as long-read support and split alignment, but BWA-MEM, which is the latest, is generally recommended for high-quality queries as it is faster and more accurate. BWA-MEM also has better performance than BWA-backtrack for 70-100bp Illumina reads	http://bio-bwa.sourceforge.net/
Bwa tools	bwa_aln	Find the SA coordinates of the input reads	http://bio-bwa.sourceforge.net/
Bwa tools	bwa_bwasw	Align query sequences in the in.fq file. When mate.fq is present, perform paired-end alignment. The paired-end mode only works for reads Illumina short-insert libraries. In the paired-end mode, BWA-SW may still output split alignments but they are all marked as not properly paired; the mate positions will not be written if the mate has multiple local hit	http://bio-bwa.sourceforge.net/
Bwa tools	bwa_bwt2sa	Generates sa files from bwt and Occ files	http://bio-bwa.sourceforge.net/
Bwa tools	bwa_bwtupdate	Update .bwt to the new format	http://bio-bwa.sourceforge.net/
Bwa tools	bwa_fa2pac	Convert FASTA to PAC format	http://bio-bwa.sourceforge.net/
Bwa tools	bwa_fastmap	Identify super-maximal exact matches	http://bio-bwa.sourceforge.net/
Bwa tools	bwa_index	Index sequences in the FASTA format	http://bio-bwa.sourceforge.net/
Bwa tools	bwa_mem	Align 70bp-1Mbp query sequences with the BWA-MEM algorithm. Briefly, the algorithm works by seeding alignments with maximal exact matches (MEMs) and then extending seeds with the affine-gap Smith-Waterman algorithm (SW)	http://bio-bwa.sourceforge.net/

Bwa tools	bwa_pac2bwt	Generate BWT from PAC	http://bio-bwa.sourceforge.net/
Bwa tools	bwa_pac2bwtgen	Alternative algorithm for generating BWT	http://bio-bwa.sourceforge.net/
Bwa tools	bwa_pemerge	Merge overlapping paired ends (EXPERIMENTAL)	http://bio-bwa.sourceforge.net/
Bwa tools	bwa_sampe	Generate alignment (paired ended)	http://bio-bwa.sourceforge.net/
Bwa tools	bwa_samse	Generate alignment (single ended)	http://bio-bwa.sourceforge.net/
Bwa tools	bwa_shm	BWA-SW for long queries	http://bio-bwa.sourceforge.net/
CD-hit	CD-hit	Very widely used program for clustering and comparing protein or nucleotide sequences	http://weizhongli-lab.org/cd-hit/
Clustalo	Clustalo	Multiple alignment of nucleic acid and protein sequences	http://www.clustal.org/omega/
Cufflinks	Cufflinks	Assembles transcripts, estimates their abundances, and tests for differential expression and regulation in RNA-Seq samples	http://cole-trapnell-lab.github.io/cufflinks/
Cutadapt	Cutadapt	Removes adapter sequences from high-throughput sequencing reads	https://cutadapt.readthedocs.io/en/stable/index.html
Deeptools	PACK	deepTools is a suite of python tools particularly developed for the efficient analysis of high-throughput sequencing data, such as ChIP-seq, RNA-seq or MNase-seq.	https://deeptools.readthedocs.io/en/develop/index.html
Deeptools	alignmentSieve	Filters alignments in a BAM/CRAM file according the the specified parameters. It can optionally output to BEDPE format, possibly with the fragment ends shifted in a custom manner	https://deeptools.readthedocs.io/en/develop/index.html
Deeptools	bamCompare	Can be used to generate a bigWig or bedGraph file based on two BAM files that are compared to each other while being simultaneously normalized for sequencing depte	https://deeptools.readthedocs.io/en/develop/index.html
Deeptools	bamCoverage	Takes an alignment of reads or fragments as input (BAM file) and generates a coverage track (bigWig or bedGraph) as output. The coverage is calculated as the number of reads per bin, where bins are short consecutive counting windows of a defined size. It is possible to extended the length of the reads to better reflect the actual fragment length. bamCoverage offers normalization by scaling factor, Reads Per Kilobase per Million mapped reads (RPKM), and 1x depth (reads per genome coverage, RPGCe	https://deeptools.readthedocs.io/en/develop/index.html
Deeptools	bamPEFragmentSize	This tool calculates the fragment sizes for read pairs given a BAM file from paired-end sequencing.	https://deeptools.readthedocs.io/en/develop/index.html
Deeptools	bigwigCompare	Normalizes and compares two bigWig files to obtain the ratio, log2ratio or difference between them	https://deeptools.readthedocs.io/en/develop/index.html
Deeptools	computeGCBias	Computes the GC-bias using Benjamini's method	https://deeptools.readthedocs.io/en/develop/index.html
Deeptools	computeMatrix reference-point	For computing the signal distribution relative to a point (reference-point), e.g., the beginning or end of each genomic region	https://deeptools.readthedocs.io/en/develop/index.html
Deeptools	computeMatrix scale-regions	For computing the signal over a set of regions (scale-regions) where all regions are scaled to the same size	https://deeptools.readthedocs.io/en/develop/index.html
Deeptools	correctGCBias	Corrects the GC-bias using the method proposed by [Benjamini & Speed (2012)	https://deeptools.readthedocs.io/en/develop/index.html

Deeptools	estimateReadFiltering	Estimates the number of reads that would be filtered given a set of settings and prints this to the terminal. Further, it tracks the number of singleton reads	https://deeptools.readthedocs.io/en/develop/index.html
Deeptools	multiBamSummary	Computes the read coverages for genomic regions for typically two or more BAM files	https://deeptools.readthedocs.io/en/develop/index.html
Deeptools	multiBigwigSummary	Given typically two or more bigWig files, computes the average scores for each of the files in every genomic region	https://deeptools.readthedocs.io/en/develop/index.html
Deeptools	plotCorrelation	Tool for the analysis and visualization of sample correlations based on the output of multiBamSummary or multiBigwigSummary	https://deeptools.readthedocs.io/en/develop/index.html
Deeptools	plotCoverage	Generates a panel of two plots. The first one simply represents the frequencies of the found read coverages, which helps you judge how relevant the mean coverage value (printed next to the sample name) is. If the distribution of read coverages is more or less homoskedatic and, ideally, normally distributed (most likely it won't be), then the mean is a very appropriate proxy for sequencing depth	https://deeptools.readthedocs.io/en/develop/index.html
Deeptools	plotEnrichment	Tool for calculating and plotting the signal enrichment in either regions in BED format or feature types (column 3) in GTF format	https://deeptools.readthedocs.io/en/develop/index.html
Deeptools	plotFingerprint	This quality control will most likely be of interest for you if you are dealing with ChIP-seq samples as a pressing question in ChIP-seq experiments is "Did my ChIP work?", i.e. did the antibody-treatment enrich sufficiently so that the ChIP signal can be separated from the background signal? (After all, around 90% of all DNA fragments in a ChIP experiment will represent the genomic background)	https://deeptools.readthedocs.io/en/develop/index.html
Deeptools	plotHeatmap	This tool creates a heatmap for scores associated with genomic regions. The program requires a matrix file generated by the tool computeMatrix	https://deeptools.readthedocs.io/en/develop/index.html
Deeptools	plotPCA	Tool for generating a principal component analysis (PCA) plot from multiBamSummary or multiBigwigSummary output. By default, the loadings for each sample in each principal component is plotted. If the data is transposed, the projections of each sample on the requested principal components is plotted instead	https://deeptools.readthedocs.io/en/develop/index.html
Deeptools	plotProfile	This tool creates a profile plot for scores over sets of genomic regions. Typically, these regions are genes, but any other regions defined in BED will work. A matrix generated by computeMatrix is required	https://deeptools.readthedocs.io/en/develop/index.html
Fasta-splitter	fasta-splitter	Divides a large FASTA file into a set of smaller, approximately equally sized files. It works with whole sequences, never dividing a sequence in the middle	http://kirill-kryukov.com/study/tools/fasta-splitter/
Fastqc	fastqc	Quality control checks on raw sequence data coming from high throughput sequencing pipelines	https://www.bioinformatics.babraham.ac.uk/projects/fastqc/
Fastq-join	fastq-join	Joins two paired-end reads on the overlapping ends	https://github.com/brwnj/fastq-join
Fastq-tools	PACK	A collection of small and efficient programs for performing some common and uncommon tasks with FASTQ files.	https://homes.cs.washington.edu/~dcjones/fastq-tools/
Fastq-tools	fastq-grep	Find reads matching a regular-expressionp	https://homes.cs.washington.edu/~dcjones/fastq-tools/

Fastq-tools	fastq-kmers	Print kmer counts for the given kmer size.Output is in two tab-seperated columns for kmer and frequency	https://homes.cs.washington.edu/~dcjones/fastq-tools/
Fastq-tools	fastq-match	Perform Smith-Waterman local alignment of a query sequence against each sequence in a fastq file	https://homes.cs.washington.edu/~dcjones/fastq-tools/
Fastq-tools	fastq-sample	Sample random reads from a FASTQ file.	https://homes.cs.washington.edu/~dcjones/fastq-tools/
Fastq-tools	fastq-sort	Concatenate and sort FASTQ files and write to standard output	https://homes.cs.washington.edu/~dcjones/fastq-tools/
Fastq-tools	fastq-uniq	Output a non-redundant FASTQ file, in which there are no duplicate reads. (Warning: this program can be somewhat memory intensive)	https://homes.cs.washington.edu/~dcjones/fastq-tools/
Fastx tool kit	PACK	The FASTX-Toolkit is a collection of command line tools for Short-Reads FASTA/FASTQ files preprocessing	http://hannonlab.cshl.edu/fastx_toolkit/
Fastx tool kit	FASTA Clipping Histogram	Create a Linker Clipping Information Histogram	http://hannonlab.cshl.edu/fastx_toolkit/
Fastx tool kit	FASTA Formatter	Changes the width of sequences line in a FASTA filer	http://hannonlab.cshl.edu/fastx_toolkit/
Fastx tool kit	FASTA nucleotides changer	Convets FASTA sequences from/to RNA/DNA	http://hannonlab.cshl.edu/fastx_toolkit/
Fastx tool kit	FASTQ Quality chart	Generates a solexa quality score box-plot graph	http://hannonlab.cshl.edu/fastx_toolkit/
Fastx tool kit	FASTQ Quality Filter	Filters sequences based on quality	http://hannonlab.cshl.edu/fastx_toolkit/
Fastx tool kit	FASTQ/A Artifacts Filter	Remove artifacts in FAST Q/A	http://hannonlab.cshl.edu/fastx_toolkit/
Fastx tool kit	FASTQ/A Clipper	Remove sequencing adapters/linkers	http://hannonlab.cshl.edu/fastx_toolkit/
Fastx tool kit	FASTQ/A Collapser	Collapse identical sequences in a FASTQ/A file into a single sequence (while maintaining reads counts)	http://hannonlab.cshl.edu/fastx_toolkit/
Fastx tool kit	FASTQ/A Nucleotide Distribution chart	FASTA-Q Nucleotide Distribution Plotter	http://hannonlab.cshl.edu/fastx_toolkit/
Fastx tool kit	FASTQ/A Quality Statistics	Output quality statistics from FASTQ file in tab delimited text	http://hannonlab.cshl.edu/fastx_toolkit/
Fastx tool kit	FASTQ/A Renamer	Renames the sequence identifiers in FASTQ/A file	http://hannonlab.cshl.edu/fastx_toolkit/
Fastx tool kit	FASTQ/A Reverse Complement	Produce the reverse-complement of each sequence in a FASTQ/FASTA file	http://hannonlab.cshl.edu/fastx_toolkit/
Fastx tool kit	FASTQ/A Trimmer	Shortening reads in a FASTQ or FASTQ files (removing barcodes or noise)	http://hannonlab.cshl.edu/fastx_toolkit/
Fastx tool kit	FASTQ-to-FASTA	Convert FASTQ files to FASTA files	http://hannonlab.cshl.edu/fastx_toolkit/
Fastx tool kit	FASTX Barcode Splitter	This program reads FASTA/FASTQ file and splits it into several smaller file, based on barcode matching	http://hannonlab.cshl.edu/fastx_toolkit/
Flux-simulator	flux-simulator	Modeling RNA-Seq experiments in silico: sequencing reads are produced from a reference genome according annotated transcripts.	http://sammeth.net/confluence/display/SIM/Home
Gatk	gatk	Toolkit for variant discovery and genotyping	https://software.broadinstitute.org/gatk/
Glimmerhmm	glimmerhmm	Gene finder based on a Generalized Hidden Markov Model (GHMM)	https://ccb.jhu.edu/software/glimmerhmm/
Gmap-fusion	gmap-fusion	Utility for identifying candidate fusion transcripts based on transcript sequences reconstructed via RNA-Seq de novo transcriptome assembly.	https://github.com/GMAP-fusion
Humann2	humann2	HUMAnN2: The HMP Unified Metabolic Analysis Network 2	http://huttenhower.sph.harvard.edu/humann2
Kallisto	PACK	Program for quantifying abundances of transcripts from RNA-Seq data, or more generally of target sequences using high-throughput sequencing reads. It is based on the novel idea of pseudoalignment for rapidly determining the compatibility of reads with targets, without the need for alignment	https://pachterlab.github.io/kallisto/

Kallisto	kallisto h5dump	Converts HDF5-formatted results to plaintext	https://pachterlab.github.io/kallisto/
Kallisto	kallisto index	Kallisto index builds an index from a FASTA formatted file of target sequences	https://pachterlab.github.io/kallisto/
Kallisto	kallisto inspect	Inspects and gives information about an index	https://pachterlab.github.io/kallisto/
Kallisto	kallisto pseudo	Computes equivalence classes for reads and quantifies abundances	https://pachterlab.github.io/kallisto/
Kallisto	kallisto quant	Computes equivalence classes for reads and quantifies abundances	https://pachterlab.github.io/kallisto/
Khmer	PACK	Library and suite of command line tools for working with DNA sequence. It is primarily aimed at short-read sequencing data such as that produced by the Illumina platform. khmer takes a k-mer-centric approach to sequence analysis, hence the name.	https://khmer.readthedocs.io/en/v1.1/index.html
Khmer	abundance-dist-single.py	Calculate the abundance distribution of k-mers from a single sequence file	https://khmer.readthedocs.io/en/v1.1/index.html
Khmer	abundance-dist.py	Calculate abundance distribution of the k-mers in the sequence file using a pre-made k-mer countgraph.	https://khmer.readthedocs.io/en/v1.1/index.html
Khmer	annotate-partitions.py	Annotate sequences with partition IDs.	https://khmer.readthedocs.io/en/v1.1/index.html
Khmer	Count-median.py	Count k-mers summary stats for sequences	https://khmer.readthedocs.io/en/v1.1/index.html
Khmer	Count-overlap.py	Count the overlap k-mers which are the k-mers appearing in two sequence datasets	https://khmer.readthedocs.io/en/v1.1/index.html
Khmer	Do-partition.py	Load, partition, and annotate FAST[AQ] sequences	https://khmer.readthedocs.io/en/v1.1/index.html
Khmer	Extract-long-sequences.py	Extract FASTQ or FASTA sequences longer than specified length (default: 200 bp)	https://khmer.readthedocs.io/en/v1.1/index.html
Khmer	Extract-paired-reads.py	Take a mixture of reads and split into pairs and orphans	https://khmer.readthedocs.io/en/v1.1/index.html
Khmer	Extract-partitions.py	Separate sequences that are annotated with partitions into grouped files	https://khmer.readthedocs.io/en/v1.1/index.html
Khmer	Fastq-to-fasta.py	Converts FASTQ format (.fq) files to FASTA format (.fa)	https://khmer.readthedocs.io/en/v1.1/index.html
Khmer	Filter-abund.py	Trim sequences at a minimum k-mer abundance	https://khmer.readthedocs.io/en/v1.1/index.html
Khmer	Filter-abund-single.py	Trims sequences at a minimum k-mer abundance (in memory version)	https://khmer.readthedocs.io/en/v1.1/index.html
Khmer	Filter-stoptags.py	Trim sequences at stoptags	https://khmer.readthedocs.io/en/v1.1/index.html
Khmer	Find-knots.py	Find all highly connected k-mers	https://khmer.readthedocs.io/en/v1.1/index.html
Khmer	Interleave-reads.py	Produce interleaved files from R1/R2 paired files	https://khmer.readthedocs.io/en/v1.1/index.html
Khmer	load-graph.py	Load sequences into the compressible graph format plus optional tagset	https://khmer.readthedocs.io/en/v1.1/index.html
Khmer	load-into-counting.py	Build a k-mer countgraph from the given sequences	https://khmer.readthedocs.io/en/v1.1/index.html
Khmer	make-initial-stoptags.py	Find an initial set of highly connected k-mers	https://khmer.readthedocs.io/en/v1.1/index.html
Khmer	merge-partitions.py	Merge partition map '.pmap' files	https://khmer.readthedocs.io/en/v1.1/index.html
Khmer	normalize-by-median.py	Do digital normalization (remove mostly redundant sequences)	https://khmer.readthedocs.io/en/v1.1/index.html
Khmer	partition-graph.py	Partition a sequence graph based upon waypoint connectivity	https://khmer.readthedocs.io/en/v1.1/index.html
Khmer	readstats.py	Display summary statistics for one or more FASTA/FASTQ files	https://khmer.readthedocs.io/en/v1.1/index.html
Khmer	sample-reads-randomly.py	Uniformly subsample sequences from a collection of files	https://khmer.readthedocs.io/en/v1.1/index.html
Khmer	split-paired-reads.py	Split interleaved reads into two files, left and right	https://khmer.readthedocs.io/en/v1.1/index.html
Khmer	trim-low-abund.py	Trim low-abundance k-mers using a streaming algorithm	https://khmer.readthedocs.io/en/v1.1/index.html

miRDeep2 kit	PACK	Discovers active known or novel miRNAs from deep sequencing data	https://github.com/rajewsky-lab/mirdeep2
miRDeep2 kit	collapse_reads	Collapses reads in the fasta file to ensure that each sequence only occurs once. To indicate how many times reads the sequence represents, a suffix is added to each fasta identifier. E.g. a sequence that represents ten reads in the data will have the '_x10' suffix added to the identifier.	https://github.com/rajewsky-lab/mirdeep2
miRDeep2 kit	excise_precursors_iterative	Collapses reads in the fasta file to ensure that each sequence only occurs once. To indicate how many times reads the sequence represents, a suffix is added to each fasta identifier. E.g. a sequence that represents ten reads in the data will have the '_x10' suffix added to the identifier.	https://github.com/rajewsky-lab/mirdeep2
miRDeep2 kit	mapper	Processes reads and/or maps them to the reference genome	https://github.com/rajewsky-lab/mirdeep2
miRDeep2 kit	mirdeep2	Wrapper function for the miRDeep2.pl program package. The script runs all necessary scripts of the miRDeep2 package to perform a microRNA detection deep sequencing data anlysis.	https://github.com/rajewsky-lab/mirdeep2
miRDeep2 kit	quantifier	Maps deep sequencing reads to predefined miRNA precursors and determines by that the expression of the corresponding miRNAs. First, the predefined mature miRNA sequences are mapped to the predefined precursors. Optionally, predefined star sequences can be mapped to the precursors too. By that the mature and star sequence in the precursors are determined. Second, the deep sequencing reads are mapped to the precursors. The number of reads falling into an interval 2nt upstream and 5nt downstream of the mature/star sequence is determined.	https://github.com/rajewsky-lab/mirdeep2
Tophat	tophat	Aligns RNA-Seq reads to a genome in order to identify exon-exon splice junctions. It is built on the ultrafast short read mapping program Bowtie.	https://ccb.jhu.edu/software/tophat/index.shtml
Trim-galore	trim-galore	Wrapper script to automate quality and adapter trimming as well as quality control, with some added functionality to remove biased methylation positions for RRBS sequence files (for directional, non-directional (or paired-end) sequencing).	https://www.bioinformatics.babraham.ac.uk/projects/trim_galore/
