# Supplementary material for: Bioportainer Workbench: a versatile and user-friendly system that integrates implementation, management, and use of bioinformatics resources in Docker environments
Source: Gigascience. 2019 Apr 25;8(4):giz041. doi: 10.1093/gigascience/giz041 (PMC6482343; doi:10.1093/gigascience/giz041)
Supplement: Response_to_Reviewer_Comments_Original_Submission.pdf [file giz041_response_to_reviewer_comments_original_submission.pdf]

Author's Response To Reviewer Comments

Close

Answers to Reviewers:

Reviewer #1: The authors have implemented a customized version of Portainers, targeted to bioinformatics. This provides a tool for easy deployment of Docker containers that are aggregated from a range of bioinformatics container repositories.
A key theme in this paper is that BioPortainers enables easy access and usability of bioinformatics tools and pipelines. The first part regarding easy access is covered by the seamless installation of BioPortainers (a single command on a Linux terminal, and Linux as an operating system can be installed very easily by non-experts), and that it allows researchers to easily deploy containers with pre-configured bioinformatics tools. However, more data are requested by the authors regarding the second aspect, on how BioPortainers helps increasing the usability of bioinformatics tools as claimed in the paper.
Specifically, the authors should discuss how after deploying a bioinformatics tool in a Docker container through BioPortainers, how do the users actually run the tool - do the BioPortainers offer an option to connect to the command line of the container running the tool, or users have to do that manually (and how)? Furthermore, how would users feed input data from the tools running inside the container started through the BioPortainers graphical interface ? For this, does BioPortainers offer the option through graphical interface operations for "docker mount" (with the user's specified directory) or attaching Docker volumes to a container ?
One example of a container run with bioinformatics pipeline (which consists of a set of tools) from beginning to end, is requested by the authors. Alternatively the authors could demonstrate runs of a few containers, each containing a single tool. In either case, it should be shown in the manuscript results or in supplementary information, how the container was started, how the users gave the input data to the tool or pipeline running in the container, how they run the tool in the container and how the got the output data.

Answer: Dear Reviewer. Thank you for your comments and suggestions, which we have considered very carefully. Thus, we took upon ourselves the task of further developing the original BioPortainer image and software, so as to further extend its capabilities beyond those already established by the Portainer project. As a result, this new version of BioPortainer (now called BioPortainer Workbench) is no longer restricted to work solely as an infrastructure interface, providing convenience to systems administrators, for installing and managing Docker containers. Now, the BioPortainer Workbench also provides users with a series of specifically developed tools (based on both CLI and GUI-dependent interfaces) to facilitate the launching of (simple and complex) bioinformatics applications and analyses (see details in the manuscript). Moreover, this new version of the manuscript brings detailed information describing the launching of several bioinformatics applications (from data input to collection of the results), using each one of these tools. The information in the paper is also complemented by tutorials and video files, available at the project´s website and in the Supplementary Materials that accompany the manuscript (The BioPortainer Workbench User Manual).

The example should be focused on a bioinformatics application, from the large range of bioinformatics Docker repositories listed in the paper as the one BioPortainers integrates with. For example, running a Docker container with a pipeline for SNP calling using NGS sequencing data (the input data are freely available from public repositories such as NCBI or EMBL) would suffice. This is key in order to demonstrate that BioPortainers provides a benefit for end-users and researchers by increasing the usability of bioinformatics tools, and it is not merely an interface providing convenience to systems administrators for installing Docker containers.

Answer: Dear Reviewer. As mentioned above, demonstration examples are shown for several applications, using alternative tools currently integrated into the BioPortainer Workbench. Among these examples, we included the execution of a Variant Calling analysis, performed with NGS sequencing data. This analysis was performed with the aid of a NextFlow pipeline (the CRG-CNAG/CalliNGS-NF pipeline), which was uploaded and launched through a new GUI-based tool, especially developed for such

purposes: the BioPortainer Pipeline Runner (see manuscript for details).

Reviewer #2: The authors developed Portainer-based bioinformatics tools platfrom called BioPortainer,that provides a graphic orchestrator of Docker environments and running them.
After try to use these functions, I have the following commented that the authors need to address:
- BioPortainer project in addition to adding some docker tools, needs to be compare BioPortainer with Portainer in detail.

Answer: Dear Reviewer. Thank you for your comments and suggestions, which we have considered very carefully. Thus, we took upon ourselves the task of further developing the original BioPortainer image and software, so as to further extend its capabilities beyond those already established by the Portainer project. As a result, this new version of BioPortainer (now called BioPortainer Workbench) is no longer restricted to work solely as an infrastructure interface, providing convenience to systems administrators, for installing and managing Docker containers. Now, the BioPortainer Workbench also provides users with a series of specifically developed tools (not originally present in Portainer, and based on both CLI and GUI-dependent interfaces) to facilitate the launching of (simple and complex) bioinformatics applications and analyses (see details in the manuscript). Moreover, the BioPortainer Workbench presents a series of unique computational resources, when compared to Portainer, such as the possibility of running GPU-accelerated applications (with the aid of a NVIDIA-Docker plug-in) and the implementation of Docker-in-Docker (DinD) environments, allowing additional containerization of processes, thus improving safety and management of resources. Finally, the BioPortainer Workbench also offers unique resources that help to ensure replicability and reproducibility of data analysis (a major concern in bioinformatics research), by allowing the exchange of detailed protocols and executions (with the aid of the Juyter Notebook, or by employing the unique BioPortainer Job Runner tool). All these aspects, which clearly differentiate the BioPortainer Workbench from the original Portainer project, are thoroughly described and discussed throughout this new version of the manuscript (see manuscript for details).

- It is more flexible and practical loading JSON file in 'docker run ...'commands.
Answer: Dear Reviewer. As mentioned above, the BioPortainer Workbench has been thoroughly revised since our original submission, incorporating a series of modifications in the original source codes, to implement several new tools. During the implementation of such modifications, the JSON files have also been incorporated into the program´s source code, so it is no longer necessary to load JSON files through Docker commands.
- In the'images' interface, after'pull the image'start up, 'deployment in progress'how to stop or cancel, it is essential add a progress bar.
Answer: Dear Reviewer. As we are sure you are aware, the full installation of Docker images/containers is not a continuous process. In fact, such installations occur in multiple computational layers and involve several processes that are executed in parallel, making it difficult to provide a reliable estimate for the whole installation progress. Nonetheless, to provide users with real-time data regarding command execution, the software is now equipped with a loading spinner, which shows up whenever users click on command buttons (such as “Deploy Container”, for example). The spinner will disappear only after the command is fully executed.

Reviewer #3: The authors present BioPortainer that includes a graphical user interface to Docker orchestration tools and is designed for bioinformatics applications. The manuscript is well written and the tool is technically sound.
The authors did a great job citing existing efforts in Docker based tools for bioinformatics applications. The abstract mentioned that BioPortainer is "a Portainer fork specifically designed for bioinformatics related Docker applications". Portainer is also cited in the text as reference [21] and the "Portainer UI" was also mentioned on page 2. However, it is unclear what are the specific technical advances that BioPortainer has achieved beyond Portainer, other than bioinformatics applications in the template catalog. Please elaborate the technical advances in the manuscript.

Answer: Dear Reviewer. Thank you for your comments and suggestions, which we have considered very carefully. Thus, we took upon ourselves the task of further developing the original BioPortainer image and software, so as to further extend its capabilities beyond those already established by the Portainer project. As a result, this new version of BioPortainer (now called BioPortainer Workbench) is no longer restricted to work solely as an infrastructure interface, providing convenience to systems administrators, for installing and managing Docker containers. Now, the BioPortainer Workbench also provides users with a series of specifically developed tools (based on both CLI and GUI-dependent interfaces) to

facilitate launching of (simple and complex) bioinformatics applications and analyses (see details in the manuscript). Moreover, the BioPortainer Workbench presents a series of unique computational resources, when compared to Portainer, such as the possibility of running GPU-accelerated applications (with the aid of a NVIDIA-Docker plug-in) and the implementation of Docker-in-Docker (DinD) environments, allowing additional containerization of processes, thus improving safety and management of resources. Finally, the BioPortainer Workbench also offers unique resources that help to ensure replicability and reproducibility of data analysis (a major concern in bioinformatics research), by allowing the exchange of detailed protocols and executions (with the aid of the Juyter Notebook, or by employing the unique BioPortainer Job Runner tool). All these aspects, which clearly differentiate the BioPortainer Workbench from the original Portainer project, are thoroughly described and discussed throughout this new version of the manuscript (see manuscript for details).

The project web site at bioportainer.ml is comprehensive, containing user documentation, video and screenshots. The reviewer would like to request a couple of case studies in the manuscript using biomedical data that show case how the 60 bioinformatics tools in the BioPortainer template catalog can be used to address biological problems.

Answer: Dear Reviewer. This new version of the manuscript shows demonstration examples for several case studies, employing the analysis of different types of biological data. These analyses are performed by a variety of bioinformatics software and employ all the alternative tools currently integrated into the BioPortainer Workbench (see manuscript for details). We trust that this new version of the manuscript brings detailed information describing the launching of these many bioinformatics applications (from data input to collection of results), using each one of these tools. The information in the paper is also complemented by tutorials and video files, available at the project´s website and in the Supplementary Materials that accompany the manuscript (The BioPortainer Workbench User Manual).

Close
