# Supplementary material for: Bioportainer Workbench: a versatile and user-friendly system that integrates implementation, management, and use of bioinformatics resources in Docker environments
Source: Gigascience. 2019 Apr 25;8(4):giz041. doi: 10.1093/gigascience/giz041 (PMC6482343; doi:10.1093/gigascience/giz041)
Supplement: Response_to_Reviewer_Comments_Revision_1.pdf [file giz041_response_to_reviewer_comments_revision_1.pdf]

Author's Response To Reviewer Comments

Close

Response to Reviewers
Reviewer reports:
Reviewer #1: The authors have addressed all the comments by the reviewer satisfactorily, and the manuscript is recommended for acceptance publication.

Answer: Dear Reviewer. Thank you for your comments and suggestions, during the first round of peer review, which helped us to significantly improve the manuscript.

Reviewer #2: By trying out the new version of BioPortainer (now called BioPortainer Workbench),it is achieve these goals that easy to use Docker platform focused on bioinformatics, and allowing running program in any type of computing ecosystem,that help to ensure replicability and reproducibility of data analysis to researchers.Moreover, the BioPortainer Workbench also presents a series of unique computational resources,such as the possibility of running GPU-accelerated applications, the function of Docker-in-Docker environments and allowing the exchange of detailed protocols and executions with the aid of the Juyter Notebook, or unique BioPortainer Job Runner tool.
It's better to be consistent installation method of instructions document of
https://hub.docker.com/r/bioportainer/bioportainer-pipeline-runner,
https://github.com/BioPortainer/BioPortaine and BioPortainer Workbench User Manual。

Answer: Dear Reviewer. We standardized the instructions for software installation described both in the main manuscript, as well as in the current version of the BioPortainer Workbench User Manual (which accompanies the manuscript as supplementary material).
Installation instructions contained at the following URLs have also been corrected and formatted to the same standard:
https://hub.docker.com/r/bioportainer/bioportainer-pipeline-runner
https://github.com/BioPortainer/BioPortainer
https://bioportainer.github.io/BioPortainer
We appreciate your careful and thorough revision.

Close
