# Supplementary material for: Bioportainer Workbench: a versatile and user-friendly system that integrates implementation, management, and use of bioinformatics resources in Docker environments
Source: Gigascience. 2019 Apr 25;8(4):giz041. doi: 10.1093/gigascience/giz041 (PMC6482343; doi:10.1093/gigascience/giz041)
Supplement: Reviewer_1_Report_Original_Submission -- Konstantinos Krampis, PhD — 7/29/2018 Reviewed [file giz041_reviewer_1_report_original_submission.pdf]

Reviewer Report

Title: BIOPORTAINER WORKBENCH: A VERSATILE AND USER-FRIENDLY SYSTEM THAT INTEGRATES IMPLEMENTATION, MANAGEMENT AND USE OF BIOINFORMATICS RESOURCES IN DOCKER ENVIRONMENTS

Version: Original Submission **Date:** 7/29/2018

Reviewer name: Konstantinos Krampis, PhD

Reviewer Comments to Author:

The authors have implemented a customized version of Portainers, targeted to bioinformatics. This provides a tool for easy deployment of Docker containers that are aggregated from a range of bioinformatics container repositories.
A key theme in this paper is that BioPortainers enables easy access and usability of bioinformatics tools and pipelines. The first part regarding easy access is covered by the seamless installation of BioPortainers (a single command on a Linux terminal, and Linux as an operating system can be installed very easily by non-experts), and that it allows researchers to easily deploy containers with pre-configured bioinformatics tools. However, more data are requested by the authors regarding the second aspect, on how BioPortainers helps increasing the usability of bioinformatics tools as claimed in the paper.
Specifically, the authors should discuss how after deploying a bioinformatics tool in a Docker container through BioPortainers, how do the users actually run the tool - do the BioPortainers offer an option to connect to the command line of the container running the tool, or users have to do that manually (and how) ? Furthermore, how would users feed input data from the tools running inside the container started through the BioPortainers graphical interface ? For this, does BioPortainers offer the option through graphical interface operations for "docker mount" (with the user's specified directory) or attaching Docker volumes to a container ?
One example of a container run with bioinformatics pipeline (which consists of a set of tools) from beginning to end, is requested by the authors. Alternatively the authors could demonstrate runs of a few containers, each containing a single tool. In either case, it should be shown in the manuscript results or in supplementary information, how the container was started, how the users gave the input data to the tool or pipeline running in the container, how they run the tool in the container and how the got the output data.
The example should be focused on a bioinformatics application, from the large range of bioinformatics Docker repositories listed in the paper as the one BioPortainers integrates with. For example running a Docker container with a pipeline for SNP calling using NGS sequencing data (the input data are freely available from public repositories such as NCBI or EMBL) would suffice. This is key in order to demonstrate that BioPortainers provides a benefit for end-users and researchers by increasing the usability of bioinformatics tools, and it is not merely an interface providing convenience to systems administrators for installing Docker containers.

Level of Interest

Please indicate how interesting you found the manuscript: Choose an item.

Quality of Written English

Please indicate the quality of language in the manuscript: Choose an item.

Declaration of Competing Interests

Please complete a declaration of competing interests, considering the following questions:

- Have you in the past five years received reimbursements, fees, funding, or salary from an organisation that may in any way gain or lose financially from the publication of this manuscript, either now or in the future?
- Do you hold any stocks or shares in an organisation that may in any way gain or lose financially from the publication of this manuscript, either now or in the future?
- Do you hold or are you currently applying for any patents relating to the content of the manuscript?
- Have you received reimbursements, fees, funding, or salary from an organization that holds or has applied for patents relating to the content of the manuscript?
- Do you have any other financial competing interests?
- Do you have any non-financial competing interests in relation to this paper?

If you can answer no to all of the above, write 'I declare that I have no competing interests' below. If your reply is yes to any, please give details below.

No competing interests for questions 1-6.

I agree to the open peer review policy of the journal. I understand that my name will be included on my report to the authors and, if the manuscript is accepted for publication, my named report including any attachments I upload will be posted on the website along with the authors' responses. I agree for my report to be made available under an Open Access Creative Commons CC-BY license (http://creativecommons.org/licenses/by/4.0/). I understand that any comments which I do not wish to be included in my named report can be included as confidential comments to the editors, which will not be published.

I agree to the open peer review policy of the journal

To further support our reviewers, we have joined with Publons, where you can gain additional credit to further highlight your hard work (see: https://publons.com/journal/530/gigascience). On publication of this paper, your review will be automatically added to Publons, you can then choose whether or not to claim your Publons credit. I understand this statement.

Yes Choose an item.
