# Supplementary material for: Bioportainer Workbench: a versatile and user-friendly system that integrates implementation, management, and use of bioinformatics resources in Docker environments
Source: Gigascience. 2019 Apr 25;8(4):giz041. doi: 10.1093/gigascience/giz041 (PMC6482343; doi:10.1093/gigascience/giz041)
Supplement: Reviewer_3_Report_Original_Submission -- Ka Yee Yeung, Ph.D. — 8/18/2018 Reviewed [file giz041_reviewer_3_report_original_submission.pdf]

Reviewer Report

Title: BIOPORTAINER WORKBENCH: A VERSATILE AND USER-FRIENDLY SYSTEM THAT INTEGRATES IMPLEMENTATION, MANAGEMENT AND USE OF BIOINFORMATICS RESOURCES IN DOCKER ENVIRONMENTS

Version: Original Submission **Date:** 8/18/2018

Reviewer name: Ka Yee Yeung, Ph.D.

Reviewer Comments to Author:

The authors present BioPortainer that includes a graphical user interface to Docker orchestration tools and is designed for bioinformatics applications. The manuscript is well written and the tool is technically sound.
The authors did a great job citing existing efforts in Docker based tools for bioinformatics applications. The abstract mentioned that BioPortainer is "a Portainer fork specifically designed for bioinformatics related Docker applications". Portainer is also cited in the text as reference [21] and the "Portainer UI" was also mentioned on page 2. However, it is unclear what are the specific technical advances that BioPortainer has achieved beyond Portainer, other than bioinformatics applications in the template catalog. Please elaborate the technical advances in the manuscript.
The project web site at bioportainer.ml is comprehensive, containing user documentation, video and screenshots. The reviewer would like to request a couple of case studies in the manuscript using biomedical data that show case how the 60 bioinformatics tools in the BioPortainer template catalog can be used to address biological problems.

Level of Interest

Please indicate how interesting you found the manuscript: Choose an item.

Quality of Written English

Please indicate the quality of language in the manuscript: Choose an item.

Declaration of Competing Interests

Please complete a declaration of competing interests, considering the following questions:

- Have you in the past five years received reimbursements, fees, funding, or salary from an organisation that may in any way gain or lose financially from the publication of this manuscript, either now or in the future?
- Do you hold any stocks or shares in an organisation that may in any way gain or lose financially from the publication of this manuscript, either now or in the future?

- Do you hold or are you currently applying for any patents relating to the content of the manuscript?
- Have you received reimbursements, fees, funding, or salary from an organization that holds or has applied for patents relating to the content of the manuscript?
- Do you have any other financial competing interests?
- Do you have any non-financial competing interests in relation to this paper?

If you can answer no to all of the above, write 'I declare that I have no competing interests' below. If your reply is yes to any, please give details below.

I declare that I have no competing interests.

I agree to the open peer review policy of the journal. I understand that my name will be included on my report to the authors and, if the manuscript is accepted for publication, my named report including any attachments I upload will be posted on the website along with the authors' responses. I agree for my report to be made available under an Open Access Creative Commons CC-BY license (http://creativecommons.org/licenses/by/4.0/). I understand that any comments which I do not wish to be included in my named report can be included as confidential comments to the editors, which will not be published.

I agree to the open peer review policy of the journal

To further support our reviewers, we have joined with Publons, where you can gain additional credit to further highlight your hard work (see: https://publons.com/journal/530/gigascience). On publication of this paper, your review will be automatically added to Publons, you can then choose whether or not to claim your Publons credit. I understand this statement.

Yes Choose an item.
